# A Review of Fault Diagnosis and Intelligent Operations and Maintenance for Agricultural Machinery: Fault Mechanisms, Key Technologies, and Practical Recommendations

**DOI:** 10.3390/s26175679

**Published:** 2026-09-07

**Authors:** Yu Zhang, Xingzhu Qian, Ruifan Tang, Chenyu Xi, Tingrui Cui, Zhong Tang

**Affiliations:** School of Agricultural Engineering, Jiangsu University, Zhenjiang 212013, China; 3240306082@stmail.ujs.edu.cn (Y.Z.);

**Keywords:** agricultural machinery, fault mechanisms, trustworthy diagnosis, fault prognostics, remaining useful life, intelligent operations and maintenance, closed-loop repair workflow, maturity assessment

## Abstract

Agricultural machinery operates under variable loads, impacts, dust, and changing soil–crop interactions, allowing faults to propagate through energy, material, information, and control pathways. This qualitative review synthesizes 195 research publications across fault formation, trustworthy diagnosis, prognostics and proactive risk control, maintenance and recovery, and case-based evidence assessment. Empirical findings are reported separately from review-derived recommendations, the authors’ conceptual requirements, and mandatory provisions of applicable standards or law. The literature most consistently supports controlled-fault identification, selected single-machine field monitoring, and localized operational compensation. Evidence is weaker for transfer across machines and seasons, calibrated prognostics, safety authorization, post-repair verification, and fleet-scale deployment. On this basis, the review recommends mission profile-specific fault boundaries, traceable diagnostic outputs, explicit uncertainty and abstention, and risk decisions linked to remaining work and resources. It also proposes a diagnostic passport, risk evolution trajectory, repair-effectiveness label, case-evidence matrix, and closed-loop repair workflow as review-level organizing constructs. No included study continuously followed the same machine or fleet through diagnosis, prognosis, authorization, maintenance, acceptance, and feedback; the framework therefore connects evidence-supported stages theoretically rather than claiming end-to-end validation.

## 1. Introduction

### 1.1. Background and Problem Statement

Agricultural machinery comprises complex electromechanical systems that connect power supply, task execution, and agronomic outcomes [1]. Tractors, combine harvesters, seeders, sprayers, and unmanned platforms are exposed to variable speed and load, impact, and harsh environments. They also operate across regions, are used intensively during short seasons, frequently exchange implements, and interact with changing working materials [2,3,4,5,6]. Fault effects can propagate through power, material, information, and control pathways, reducing output capacity and operational quality while increasing energy use, safety risks, and farming delay losses. Because sowing, crop protection, and harvesting have narrow seasonal windows, even brief downtime can cause missed operations, delayed harvests, and yield losses [7]. Agricultural machinery reliability is therefore not solely a mechanical design problem; it also depends on condition assessment, risk judgment, and organizational execution.

Conventional maintenance relies primarily on operator experience, scheduled inspection, and post-fault disassembly. These practices remain useful for structurally simple equipment operating under stable conditions, but they are insufficient for large, intelligent, and electrified machines. Mechanical, hydraulic, electrical, control, and implement systems are coupled, and the same symptom may arise from different upstream causes. A single threshold therefore cannot reliably separate normal load variation from physical damage. Real-world faults are infrequent, develop over long periods, and depend strongly on context; bench tests, artificially introduced faults, and randomly sliced samples do not fully represent natural degradation in the field [8]. Many studies report detection accuracy without jointly addressing fault authenticity, generalization across operating conditions, probability calibration, operational quality, safety response, and post-repair recovery. Diagnosis, prognostics, and repair consequently remain weakly connected by auditable evidence.

The literature therefore supports a transition from predominantly corrective maintenance toward condition- and risk-informed operations and maintenance for agricultural machinery. In this review, intelligence denotes cross-checking fault mechanisms, condition evidence, prognostic uncertainty, personnel authority, resource availability, and mission objectives within a traceable workflow; it does not denote algorithmic complexity alone. The resulting closed loop is an organizing proposal spanning the physical system, information system, and repair organization.

### 1.2. Review Objectives, Research Questions, and Organizing Framework

This review neither proposes a new diagnostic algorithm nor ranks individual models. Instead, it addresses five questions: How do loads and damage processes generate and propagate faults in agricultural machinery? How can a current abnormality be diagnosed with sufficient trustworthiness? How should future risk be expressed under uncertainty? How should safe repair and recovery proceed after a fault? Which parts of this chain are supported by existing cases? The review therefore examines fault mechanisms and classification, current state diagnosis, health prognostics and proactive control, post-fault repair decision-making and recovery, and the maturity of the available case evidence.

**Figure 1:**
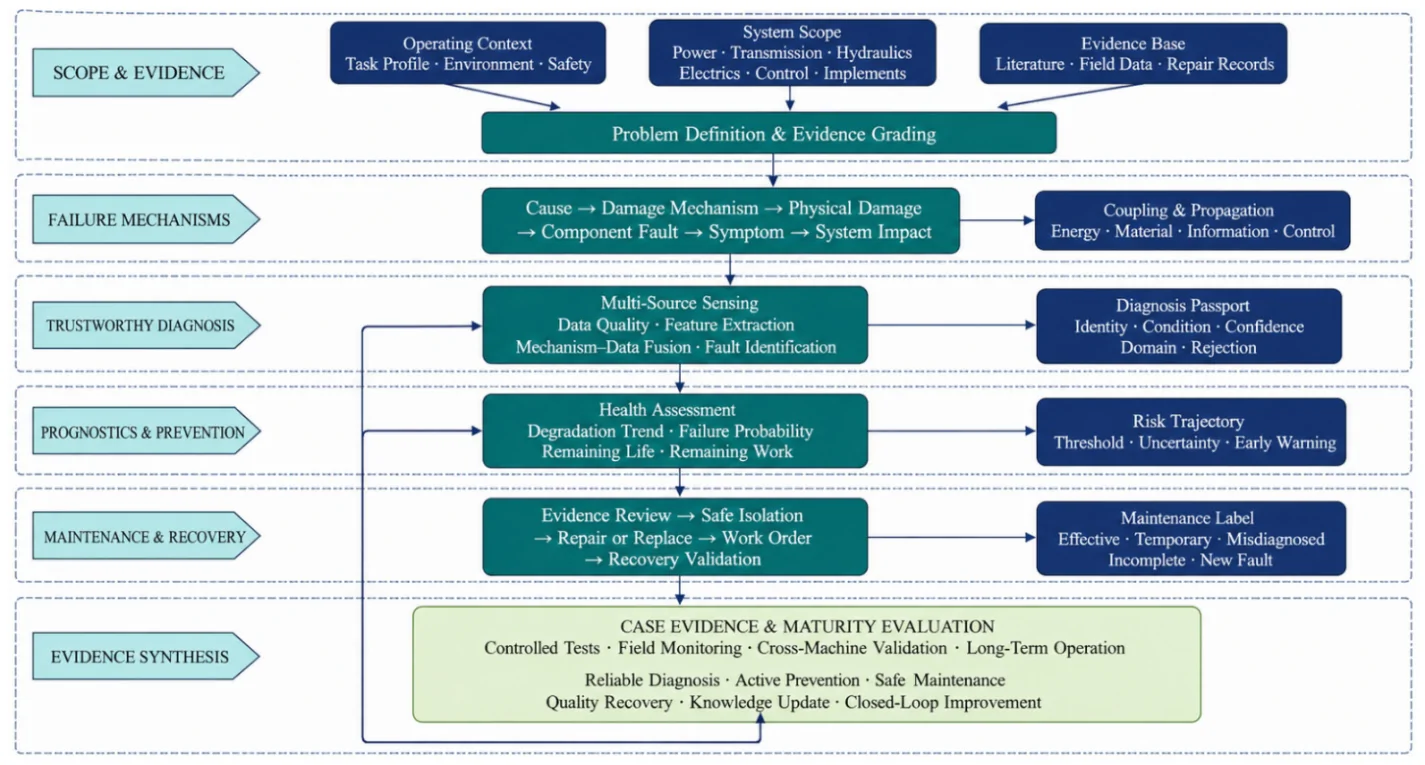
Overall logical framework of agricultural machinery fault diagnosis and intelligent operations and maintenance.

The main contribution is a unified analytical framework that connects fault diagnosis, prognostics, maintenance decisions, repair engineering, and agricultural operational quality assessment through explicit evidence and responsibility interfaces. It does not claim a new diagnostic algorithm. The framework introduces five review-level constructs: a diagnostic passport for auditable delivery of current state evidence; a risk evolution trajectory for linking time-varying health, exposure, consequence, and action; a repair-effectiveness label for preserving verified post-repair outcomes; a case-evidence matrix for separating observations from author reconstruction and missing links; and a closed-loop repair workflow for routing evidence through isolation, restoration or replacement, resource execution, acceptance, and feedback. These constructs draw on PHM, predictive and condition-based maintenance, digital twin and digital thread concepts, self-healing, and maintenance decision research, but adapt and recombine them for agricultural mission profiles, implements, farming windows, operational quality, and human authorization. They remain conceptual proposals unless identified otherwise and have not been validated together on one machine. Figure 1 summarizes the organizing framework, and Table 1 states the incremental contribution and validation boundary of each construct.

### 1.3. Core Concepts and Terminology

To maintain consistent state semantics throughout the review, normal condition, degradation, fault, and failure are treated as continuous but not evenly spaced states. A machine is normal when it satisfies power, operational quality, energy use, and safety requirements under a specified mission profile. Degradation denotes a departure of a physical or performance variable from its healthy baseline while the required function and safety constraints remain satisfied. A fault is a confirmed abnormality in a component, interface, or control function that has reduced functionality or materially increased the risk of failure. Failure occurs when the machine can no longer perform its required function, meet the minimum safety requirement, or achieve the required operational quality. A fault does not necessarily require immediate shutdown, and degradation should not be classified as a fault without correcting for operating conditions.

Condition monitoring observes operating conditions continuously or in response to events. Fault detection determines whether the current state is abnormal. Fault diagnosis identifies the type, location, cause, and severity of a current fault and reports the supporting evidence and confidence. Health assessment describes the current health level and degradation stage, whereas fault prognostics estimates future health trajectories, failure probabilities, remaining operational capacity, or remaining useful life. In Section 6, entry into the repair process is not defined simply by the occurrence of a fault. It occurs when the current state diagnosis triggers safe isolation, functional restoration, or manual intervention, or when future risk can no longer be controlled through proactive measures.

The review covers tractors, combine harvesters, seeders, crop protection equipment, electrified components, agricultural robots, and unmanned aerial vehicles, together with their power, transmission, running gear, hydraulic, electrical, electronic, control, communication, and working systems. In this review, the diagnostic passport is an evidence delivery record for a current diagnosis; the risk evolution trajectory is a time-indexed record of health, exposure, consequence, uncertainty, and candidate action; the repair-effectiveness label records whether recovery is true, temporary, failed, misdirected, or repair-induced and under what verification conditions; the case-evidence matrix records which workflow stages are observed, inferred, or absent; and the closed-loop repair workflow specifies how evidence is reviewed, authorized, executed, verified, and fed back. These are theoretical organizational tools synthesized for agricultural machinery, not established standards or proof of end-to-end field validation.

### 1.4. Limitations of Existing Reviews and Incremental Contribution

Existing reviews commonly organize the field by subsystem, sensor, algorithm family, or maintenance technology. PHM, predictive and condition-based maintenance explain state assessment and intervention timing; digital twins and digital threads emphasize lifecycle state continuity; self-healing emphasizes local automatic recovery; and maintenance decision frameworks optimize actions and resources. Their scopes are complementary, but they do not usually connect agricultural mission boundaries, machine implement identity, abstention, human authorization, post-repair operational quality acceptance, and evidence maturity in one traceable chain. The incremental contribution of this review is therefore a transparent agricultural synthesis and a set of interfaces, not a claim that the underlying PHM, prognostic, digital, or maintenance principles originated here.

## 2. Literature Search Strategy and Review Methods

This article is a qualitative review (review), rather than a systematic review or a scoping review. PRISMA-ScR is used only as a reporting reference to improve the transparency of literature identification, screening, data extraction, and evidence mapping; its use does not change the methodological classification of the article. The review aims to integrate and critically interpret heterogeneous evidence spanning fault mechanisms, diagnosis, prognostics, maintenance, and post-repair verification rather than to estimate a pooled intervention effect. Because the included studies differ substantially in equipment architecture, fault definitions, units of analysis, validation designs, and reported outcomes, no meta-analysis or formal pooled risk-of-bias synthesis was undertaken. Nevertheless, a predefined search and screening procedure, independent screening, standardized extraction fields, and an auditable evidence ledger were used to improve reproducibility.

### 2.1. Literature Sources and Scope

The Web of Science Core Collection, China National Knowledge Infrastructure (CNKI), and Google Scholar were used as literature sources. Web of Science provided English-language journal research in agricultural engineering, mechanical engineering, reliability, automation, and intelligent manufacturing. CNKI supplemented Chinese-language studies on fault mechanisms, condition monitoring, diagnosis, repair, and field applications. Google Scholar was used to identify interdisciplinary studies not adequately indexed by the other databases and to support backward and forward citation searching. Records were exported or saved to Zotero using Zotero Connector. Database name, search date, query, and source-level count were recorded in the search log before deduplication; duplicate candidates were then checked using DOI, title, author, publication year, and article content.

The search covered June 2016 to June 2026 and was completed on 8 July 2026. Eligible publication types were primarily original journal articles and reviews. Other important publications were considered when they provided sufficiently complete technical information on machinery structure, fault data, field experiments, repair procedures, or regulatory constraints. Conference abstracts, news reports, promotional material, patent specifications, duplicate publications, and records presenting only a research concept without substantive technical content were excluded from the final synthesis.

No geographic restriction was imposed, but each publication had to inform at least one stage of the agricultural machinery fault management pathway: fault mechanisms, current state diagnosis, risk prognostics, preventive control, or post-fault repair. Cross-industry evidence was retained only when direct agricultural evidence was insufficient and at least one of five transferability criteria was satisfied: similarity of the physical failure mechanism; comparability of signals and sensing arrangements; overlap of loads and operating regimes; equivalence of the diagnostic, prognostic, or maintenance task; and comparability of decision, safety, or recovery outcomes. Such publications were labeled as cross-domain methodological evidence, and their reported performance or economic benefit was not extrapolated directly to agricultural machinery.

### 2.2. Search Terms

To balance search coverage and topical relevance, keywords were organized into four groups: equipment, fault phenomena, technical tasks, and operation and maintenance activities. Equipment terms included agricultural machinery, tractors, combine harvesters, seeders, seed meters, sprayers, crop protection unmanned aerial vehicles (UAVs), agricultural robots, and autonomous agricultural vehicles. Fault-related terms included wear, fatigue, corrosion, cracks, leakage, and faults involving gears, bearings, hydraulics, motors, sensors, actuators, control systems, or communications. Technical task terms included condition monitoring, anomaly detection, fault localization, fault diagnosis, severity estimation, health assessment, fault prognostics, early warning, remaining useful life, and uncertainty quantification. Operation and maintenance terms included preventive maintenance, maintenance decisions, safe isolation, repair, replacement, remanufacturing, spare parts scheduling, repair acceptance, and digital twins.

One comprehensive query and four task-specific queries were used to cover fault mechanisms, current state diagnosis, prognostics and early warning, and repair and recovery without forcing every term into one expression. The complete Web of Science and CNKI strings, search fields, dates, and source counts are provided in Supplementary S1; the searches covered June 2016 to June 2026 and were completed on 8 July 2026.

CNKI was searched in the Subject (SU) field using five query groups covering the comprehensive search, fault mechanisms, current state diagnosis, prognostics and early warning, and maintenance and recovery. Each group combined agricultural machinery or equipment terms with the corresponding fault, detection, prediction, maintenance, and repair terms. The complete reproducible strings, search date, and source count are reported in Supplementary S1. The search covered June 2016 to June 2026, was run on 8 July 2026, and yielded 1635 records imported into Zotero.

Google Scholar was searched on 8 July 2026 with the short English and Chinese phrase combinations listed in Supplementary S1. The platform displayed an estimated 18,508 results, but this display estimate was recorded only as context. The stopping point was page 100, and 2000 records were actually reviewed and saved through Zotero Connector; only those records entered identification and deduplication. Database-specific downstream counts could not be reconstructed because source tags were not retained in the merged library.

### 2.3. Supplementary Literature Identification

The comprehensive queries could miss studies limited to a particular component, operation, or repair process. Supplementary searches were therefore conducted for the technical domains covered in Section 3, Section 4, Section 5, Section 6, Section 7 and Section 8. Terms added for fault mechanisms included bearing contact fatigue, gear wear, corrosive wear, hydraulic contamination, lubrication failure, multiple fault coupling, and cascading propagation. Terms added for current state diagnosis included multisource sensing, sensor placement, data quality, order tracking, time-frequency analysis, knowledge graphs, domain adaptation, and physics-guided learning. Terms added for prognostics and early warning included health indicators, degradation stages, probabilistic prediction, prediction intervals, remaining useful life, small samples, and transfer across operating conditions. Terms added for repair and recovery included safe isolation, fault verification, weld overlay repair, laser cladding, additive repair, spare parts scheduling, remote repair, recovery acceptance, and repair feedback.

Object-specific supplementary searches were also conducted for seeding, crop protection, and autonomous equipment. Searches for seeding equipment used terms related to missed and duplicate seeding, seed-metering performance, compensatory seeding, and operational quality. Searches for crop protection equipment used terms related to spray flow, droplet quality, pump faults, soft sensing, and abnormal application rates. Searches for agricultural robots and UAVs used terms related to perception and positioning failures, actuator anomalies, communication interruptions, fault-tolerant control, degraded operation, human takeover, and safe stopping. These searches were intended to capture studies of individual implements that did not use the broader term “agricultural machinery fault diagnosis.”

For eligible reviews and representative studies, reference lists and citing publications were also examined. Publications identified through citation chasing were recorded as supplementary sources and subjected to the same inclusion and exclusion criteria as database records. When a traced publication and an included study used the same data and equipment and reached the same conclusion, only the version with more complete technical information or independent validation was retained, preventing duplicate evidence from being counted more than once.

### 2.4. Literature Selection

Study selection comprised record identification, deduplication, title and abstract screening, full-text eligibility assessment, and final inclusion. The searches produced 16,732 Web of Science records, 1635 CNKI records, and 2000 Google Scholar records actually saved to Zotero. Thus, 20,367 records entered the reference-management database; the 18,508 results displayed by Google Scholar were not added to this total.

A total of 2815 duplicate records were removed through automated matching and manual verification of titles, authors, publication years, DOIs, and article content, leaving 17,552 records for title and abstract screening. Records were assessed for relevance to agricultural machinery or transferable mechanical equipment, the presence of an explicit fault or degradation phenomenon, and their relation to diagnosis, prognostics, maintenance, or repair. After 16,833 topically irrelevant records had been excluded, 719 publications proceeded to full-text eligibility assessment.

**Figure 2:**
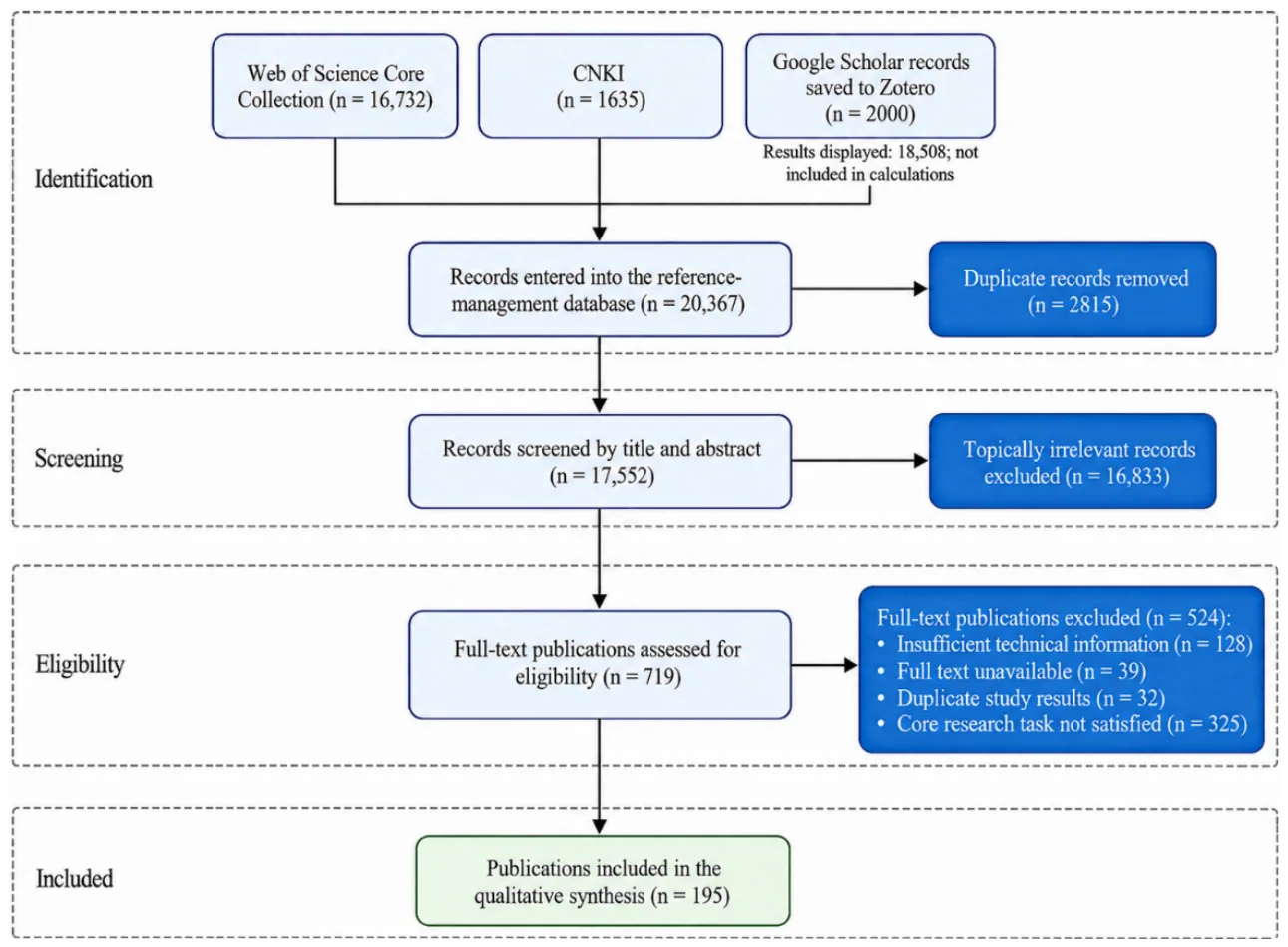
Literature screening process.

Full texts were assessed for the study object, fault-generation process, data source, technical task, validation conditions, and scope of the conclusions. We excluded 128 publications with insufficient technical information, 39 for which the full text was unavailable, 32 reporting duplicate study results, and 325 that did not satisfy a core research task. The 524 full-text exclusions left 195 publications in the final qualitative synthesis. Figure 2 reports the reconciled selection pathway.

The numerical audit was 20,367 − 2815 = 17,552 records screened; 17,552 − 16,833 = 719 full texts assessed; and 719 − 524 = 195 publications included. The Google Scholar display estimate of 18,508 is reported separately and is not used in any of these calculations.

The first and second authors independently screened titles and abstracts and independently assessed full texts against the predefined eligibility criteria. Each reviewer recorded an inclusion or exclusion decision and the applicable reason in the screening log. Disagreements were discussed by the two reviewers; cases that remained unresolved were adjudicated by the fifth author, and the consensus decision was retained in the final log.

Supplementary S1–S4 contain the database-specific queries, search dates, source counts, screening and exclusion log, final included publication list, standardized extraction fields, study-level evidence ledger, and evidence distribution summary. These materials preserve both included and excluded decisions required to audit the review process.

### 2.5. Evidence Organization and Qualitative Synthesis

Data were independently extracted by the third and fourth authors using standardized fields: publication year, equipment type, component or subsystem, fault phenomenon and formation process, data source, operating conditions, sampling unit, training–test split, technical task, output, evaluation metric, field validation, prognostic horizon, intervention, repair outcome, and study limitation. Methodological quality fields additionally recorded fault authenticity, sample independence, leakage risk, external validation, uncertainty reporting, intervention status, and post-repair follow-up. Extraction disagreements were resolved by discussion, with unresolved items referred to the fifth author; original units and statistical definitions were retained.

The third and fourth authors independently assigned all 195 included publications to one of four evidence levels. Level 1 comprised simulation, controlled laboratory or bench evidence, artificially introduced faults, fixed operating conditions, and public benchmark data. Level 2 comprised single-machine or single-site field or in-service evidence without independent machine or extended operation validation. Level 3 comprised evidence across independent machines, plots, sites, regions, companies, or seasons. Level 4 comprised end-to-end operational evidence in which a defined diagnostic, maintenance, or refurbishment method was applied in a real operating or maintenance context, followed by an actual inspection, repair, replacement, or other intervention and verification through post-intervention performance, repair time, cost, availability, or operational outcomes. Cross-domain status was retained as a separate transferability label.

The study-level evidence ledger reports equipment or domain, primary stage, validation setting, fault authenticity, independent machine information, intervention, follow-up, evidence level, and classification confidence for all 195 publications. After full-text review, 141 publications were classified as Level 1, 41 as Level 2, 9 as Level 3, and 4 as Level 4. Evidence level and methodological quality were treated as separate dimensions: a study could have mature field validation but incomplete reporting, or rigorous laboratory methods but only Level 1 external validity.

A qualitative synthesis was performed rather than a pooled effect-size analysis. This choice is consistent with the article’s positioning as a qualitative review: the selected studies differed substantially in equipment architecture, fault type, sampling unit, data partitioning, operating conditions, failure definitions, and evaluation metrics, so pooling mean accuracy or remaining useful life error would be misleading. The synthesis first identified findings supported across studies, then examined differences attributable to equipment, operating conditions, and validation design, and finally specified the strength and limits of the supporting evidence.

For fault diagnosis studies, the review emphasized fault authenticity, sample independence, validation across operating conditions, probability calibration, and the capacity to abstain when evidence was insufficient. Prognostic studies were additionally assessed for the prediction origin, future horizon, failure threshold, warning lead time, and interval coverage. Studies of prevention and repair were assessed for the basis of action triggers, personnel authorization, resource constraints, recovery acceptance, and the duration for which effectiveness was maintained. Fault classification without a prognostic horizon was not treated as evidence of remaining useful life, and a diagnostic case without documented intervention and recovery verification was not treated as evidence of a complete closed-loop repair workflow. These methods provide a common basis for comparing fault mechanisms, detection and diagnosis, prognostics and early warning, and repair and recovery in the sections that follow.

**Figure 3:**
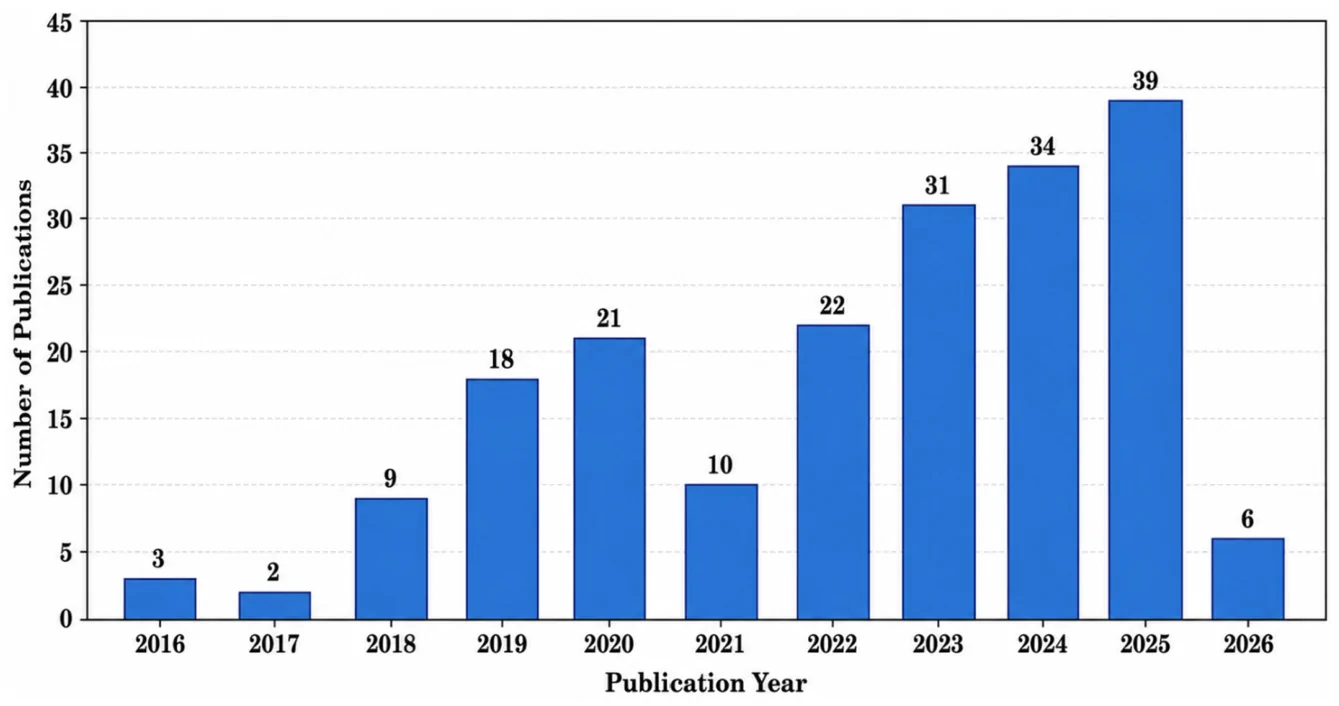
Distribution of included publications by publication year.

The annual distribution of the 195 included publications is provided in Figure 3. The figure was compiled from the publication years in the reference list, including publications from 2026.

Official statutes and regulations cited later to define compliance boundaries were treated as normative contextual sources rather than empirical studies. They were not screened as research publications and are excluded from the 195-publication evidence ledger and the evidence distribution counts in Table 2 and Table 3.

### 2.6. Cross-Industry Evidence Admission, Transfer Boundaries, and Distribution

Cross-industry evidence was screened with the explicit criteria in Table 2. The rule is deliberately asymmetric: similarity can justify examining a method, but every mismatch narrows the permitted conclusion. A study failing the mechanism-or-task requirement was excluded from framework support even if it reported high performance. Studies that passed were used only for methodological architecture, representation, validation design, uncertainty treatment, or decision interface design until agricultural validation became available.

The 195 included publications were assigned to one mutually exclusive primary framework link to prevent double counting. Table 3 separates fault mechanisms, current state diagnosis, prognostics, risk control, maintenance and repair, post-repair verification, and illustrative case mappings. The prognostics and risk-control rows subdivide the former combined category; post-repair verification is separated from maintenance and recovery. Direct agricultural and cross-industry methodological totals remain 97 and 98, respectively, and the row total remains 195.

## 3. Common Fault Modes and Formation Mechanisms in Agricultural Machinery

### 3.1. System Functions and Failure Boundaries Under Mission Profiles

**Figure 4:**
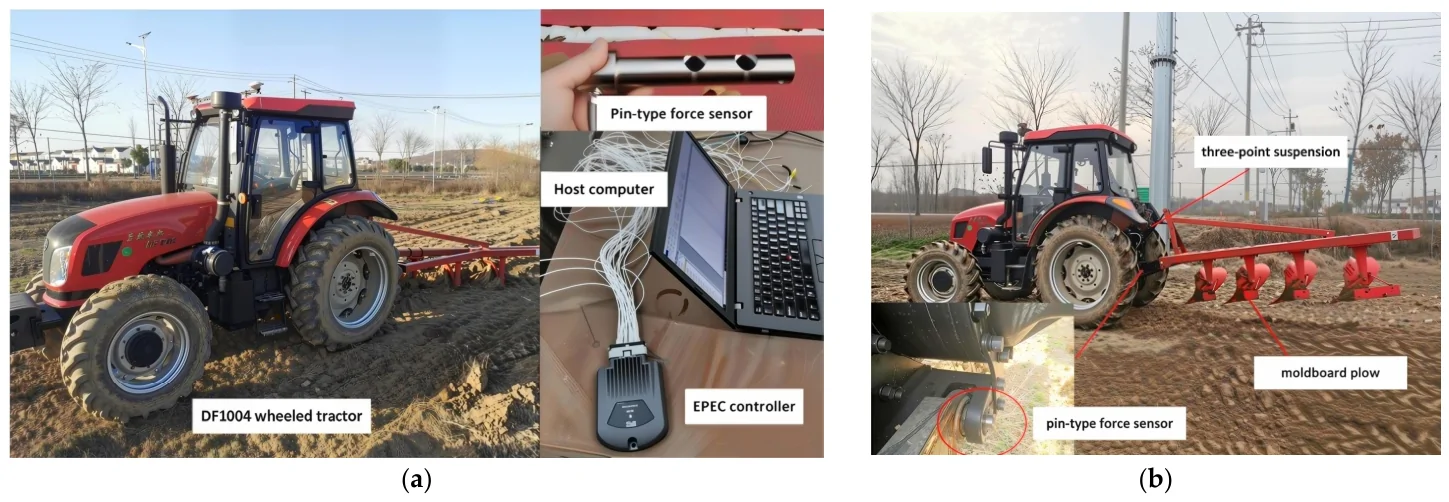
Tractor plowing operation scene and three-point suspension load sensing arrangement. (**a**) Tractor plowing resistance test scenario. (**b**) Three-point suspension structure and sensor arrangement. Source: Reproduced without modification from Yang et al. [13] under CC BY 4.0.

The system boundary of agricultural machinery should encompass energy and material inputs, power transmission, mobility and structural support, task execution, and constraints on operational output [9]. Normal operation, degradation, a fault, and failure must be defined relative to the mission profile. Degradation is present when a performance measure departs from its healthy baseline, but the specified function remains achievable. A fault exists when an abnormal component or interface has impaired function or increased the risk of failure. Failure occurs when a power, safety, or operational quality boundary is breached. Continued operation therefore does not imply the absence of a fault, and low energy consumption does not guarantee acceptable operational quality. Machine state is jointly shaped by the overall architecture, the attached implement, and the external environment [10,11]. During tractor plowing, for example, soil resistance is transmitted through the plow and three-point hitch to the tractor; the hitch interface is therefore both a critical path for power and load transfer and an informative location for measuring operational load [12]. Figure 4 shows a representative tractor-plowing test and the arrangement of sensors on the three-point hitch.

The mission profile establishes the relevant baselines and limits. Transport emphasizes speed, stability, and braking; light field work emphasizes operational continuity and energy use; heavy plowing emphasizes tractive output and structural capacity; and traction impacts increase both peak and cyclic loading. Soil resistance, gradients, surface unevenness, and changing outdoor conditions impose additional load exposure [14]. Li and Yuan reported that prolonged full-load or overload operation on complex roads increases the risk of structural failure in heavy-duty tractors. In their 720 s load history comprising nine adverse road surfaces, three critical locations determined system reliability in series, making system reliability lower than that of any single location [15]. Ignoring correlations among load channels underestimated structural reliability, whereas ignoring prestress overestimated it [16]. Thus, even at the same output, the load path and the responses of multiple locations can alter the reliability boundary.

Yang et al. separated tractor plowing into entry, acceleration, uniform-speed, deceleration, and excavation stages. Although deceleration lasted only 1.5 s, it had the largest equivalent load amplitude, 3.016759 kN [13]. The finding shows that reliability depends on load amplitude, occurrence, and sequence; this review therefore recommends retaining the staged load structure rather than representing the cycle by its mean alone.

Failure criteria can be organized into three boundary layers: performance, operational quality, and safety. The performance layer determines whether power, energy consumption, and availability remain within the range permitted by the task. The operational quality layer determines whether tractive or implement output meets the specified task requirements. The safety layer determines whether stability, protection, or control conditions could cause injury. In a synthesis of 79 studies, Fargnoli and Lombardi reported that agricultural mortality was approximately three times the industry average, that overturning was the leading cause of death among farmers, and that about 800,000 tractors in Italy had entered service before 1996 [17]. The fact that a machine remains operational therefore cannot override safety constraints.

The three boundaries are not concentric: performance degradation may precede operational quality failure, and operational quality may be unacceptable even when power output is normal. This review recommends adapting performance and quality limits to the mission profile. Safety limits, however, are governed by the applicable risk assessment, machinery-safety requirements, and legal obligations and cannot be relaxed merely because the task is time-critical.

### 3.2. Wear, Fatigue, Corrosion, and Damage Evolution

**Figure 5:**
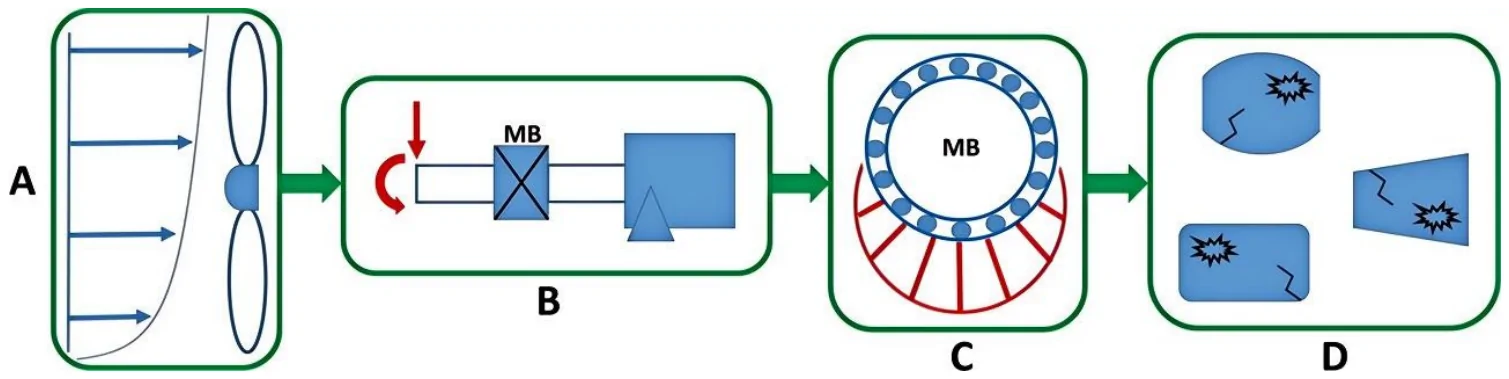
The transfer relationship between external load, internal contact load and damage failure. (**A**) wind- and control-induced rotor loads; (**B**) forces and moments reacted by the main bearing (MB); (**C**) internal roller–raceway contact-load distribution; and (**D**) resulting damage and failure. Green arrows show the cause-and-effect progression, blue arrows and shapes denote external loads and mechanical components, blue circles represent rolling elements, red arrows and radial lines indicate applied loads and internal contact loads, and black crack/star symbols indicate damage. Source: Reproduced without modification from Hart et al. [19] under CC BY 4.0.

Damage should be separated into initiating conditions, mechanisms, physical states, component fault modes, symptoms, and consequences [18]. A symptom such as vibration or temperature rise cannot by itself establish a unique cause. Figure 5 organizes the evidence as external task load, structural transmission, internal contact conditions, accumulated damage, and functional consequence; Hart et al. further show that load paths, lubrication, alignment, and contamination jointly shape this progression [19,20,21].

Bearing contact fatigue originates in repeated contact stress between rolling elements and raceways [22]. Inclusions, surface defects, eccentric loading, and excessive preload concentrate stress; intersecting cracks then produce pitting and spalling. Detached particles scratch the raceway and reduce its effective contact area, reinforcing a crack–spall–abrasive wear cycle. Insufficient lubrication promotes micropitting, adhesion, and frictional heating; transient overload changes surface geometry and clearance; and corrosion pits create new crack origins. Xu et al. attributed 48.94% of bearing failures to incorrect assembly, use, or maintenance and a further 19.15% to multiple causes, indicating that wear and fatigue seldom occur in isolation [23].

Soil-contact parts are exposed to abrasion, impact, adhesion, and corrosion, with material properties and particle shape influencing damage rate. Wang et al. reported that failures associated with key wear-resistant parts exceeded 50% of agricultural machinery failures, while domestic combine harvesters commonly operated only 20–30 h without failure and rotary tillers about 80 h [24]. These source-specific figures illustrate the importance of environmental exposure and should not be treated as universal service-life limits.

Large-main-bearing studies likewise show that design, operating, and service conditions jointly shape damage paths. Hart et al. reported that wind-turbine main-bearing failure within 20 years may reach 30%. Structural loads create internal contact loads, while lubrication, clearance, alignment, inclusions, and contamination govern damage; micropitting may develop into spalling, whose particles further accelerate abrasive wear [19]. Early rough-surface contact and microcracking may be inconspicuous; intermediate pitting, spalling, and clearance growth may coincide with vibration, noise, or heating; and advanced cracking, plastic deformation, or seizure causes clear failure. Because similar symptoms may result from imbalance, looseness, or impact—and low-speed, high-load bearings may fail without strong vibration—symptoms must be interpreted cautiously.

Transitions between damage stages should therefore retain uncertainty and be described on two axes: physical damage severity and functional consequence. Identical damage may cause only local degradation in a noncritical component but severe consequences in a load-bearing bearing, brake, or critical working edge. This two-axis description provides fault entities and severity labels for Section 4 without predetermining sensor locations or diagnostic algorithms.

### 3.3. Common Faults in Power, Transmission, Running Gear, and Hydraulic Subsystems

Agricultural machinery power and task execution can be read as one energy chain: engine generation, transmission, running gear support, and hydraulic actuation [25,26]. Interruption, attenuation, fluctuation, leakage, and incorrect transmission provide a common fault vocabulary. Xiao et al. monitored eight variables under five conditions on a tractor diesel engine; the reported ranges describe that dataset, not recommended operating limits [27]. Speed, temperature, and intake-pressure changes should therefore be interpreted against downstream transmission and hydraulic load [28].

Transmission and running gear faults arise from overload, impact, lubrication loss, contamination, clearance change, and misalignment. Hosseinpour-Zarnaq, Omid, and Biabani-Aghdam tested tractor gearbox faults at 600, 1350, and 2000 r/min and found stronger impacts and uneven rotation with mesh damage [29,30]. Gear, shaft, bearing, tire, track, and connector effects should be localized through torque transfer, traction stability, slip, drift, and implement response rather than by vibration alone [31].

Hydraulic faults similarly appear as flow or pressure interruption, attenuation, fluctuation, leakage, sticking, cavitation, or impact [32]. Gupta et al. reported a 10 μm filter, β10 of about 75%, 60 L/min rated flow, and 200 bar pressure; bypass after blockage allowed contaminants to reach pumps, valves, and transmission elements [33]. Diagnosis should compare pump and valve inlet–outlet states, actuator response, and downstream load. Across the powertrain, mismatches between engine output, transmission demand, and hydraulic load are treated as interface faults.

The hydraulic subsystem converts mechanical energy from the engine or transmission into pressure and flow and uses pumps, valves, cylinders, motors, and lines to drive working implements. Dai et al. classified hydraulic faults as stagnation, impact, cavitation, sticking, internal leakage, external leakage, or composite faults; reported causes included fluid contamination, seal degradation, pump and valve wear, inadequate suction, and temperature variation [32]. Pump wear attenuates flow, valve sticking disrupts pressure regulation, cavitation causes local impact and pressure pulsation, and actuator leakage reduces effective output force. In an onboard hydraulic system for agricultural machinery, Gupta et al. reported a filter rating of 10 μm, filtration efficiency β10 of approximately 75%, rated flow of 60 L/min, and working pressure of 200 bar. They estimated that about one million particles larger than 1 μm could enter the system each minute; after filter blockage opened the bypass valve, contaminants entered pumps, valves, and transmission components with the fluid and accelerated tribological damage [33]. Such symptoms must be interpreted together with inlet and outlet conditions at pumps and valves, actuator response, and downstream load.

These subsystems share causes such as overload, inadequate lubrication, clearance change, contamination, and thermal effects and may become coupled through their interfaces. Insufficient engine output imposes low-speed, high-load operation on the gearbox; transmission losses reduce hydraulic pump speed; and pump leakage or actuator seizure raises engine load, reducing speed and increasing temperature. Mismatches among the power source, transmission, and hydraulic load should therefore be classified as interface faults. The fault vocabulary should identify the energy-flow anomaly, component mode, initiating condition, symptom, and downstream consequence so that later chapters can define measurement variables and diagnostic targets consistently.

### 3.4. Common Faults in Electrical, Electronic, Control, Communication, and Working Systems

Electrical and electronic faults are organized along a sensing–communication–control–actuation–feedback chain [34,35,36,37]. The useful distinction is between information anomaly, control deviation, abnormal physical response, and loss of operational quality, because the same electronic defect can ultimately alter sowing depth, application rate, implement motion, or vehicle trajectory.

Sensor evidence spans pressure, temperature, position, inertial, vision, and crop condition measurements [38,39,40,41,42]. Zou et al. associated agricultural IoT sensor faults with aging, power, mounting, environment, and operator factors and classified constant output, bias, drift, noise, abrupt change, disconnection, and missing data [43]. Stale or desynchronized values should be recorded separately from real operating condition changes because they can distort load, attitude, and task state estimates.

**Figure 6:**
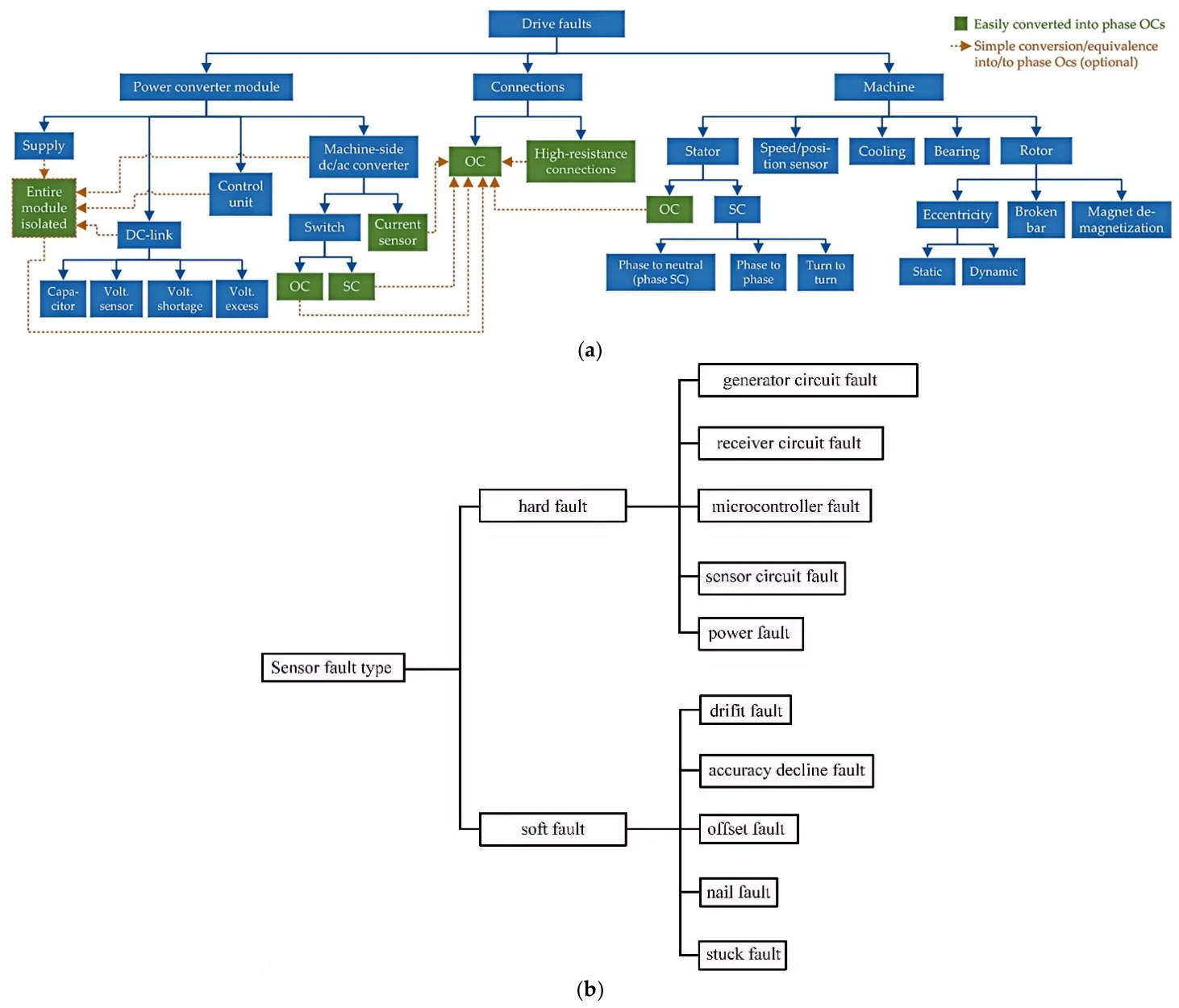
Typical fault types of agricultural machinery electric drive and sensor systems. (**a**) AC drive system fault classification. (**b**) Agricultural sensor system fault classification. Sources: (**a**) Reproduced without modification from Yepes et al. [48] under CC BY 4.0. (**b**) Reproduced without modification from Zou et al. [43] under CC BY 4.0.

Electrified powertrains add storage, converters, motors, insulation, connectors, and energy-management faults [44,45,46,47]. Scolaro et al. and Chen et al. linked winding, demagnetization, eccentricity, bearing, inverter, and thermal faults to current, back-electromotive-force, torque, and speed changes; phase redundancy may temporarily preserve function [48]. Controller resets, mode or parameter errors, latency, and command-response inconsistency further propagate through autonomous systems [49,50,51], while failed motors, valves, clutches, steering, seeding, fertilizing, and spraying mechanisms prevent commanded motion or flow [52,53,54,55,56,57,58,59,60,61,62,63,64]. Figure 6 summarizes these fault classes; the diagnostic object remains the affected interface and task consequence.

The control layer comprises the embedded controller, task logic, parameter tables, and actuation interface. Hardware faults include processor resets, input–output channel failures, and protective activation of power modules; software and logic faults include incorrect mode switching, incorrect parameter calls, failure of limit enforcement, and reversed command direction [49]. In their review of autonomous all-terrain vehicles for agriculture, Etezadi and Eshkabilov treated controllers, sensors, actuators, and communication devices as four interdependent elements and noted that complex terrain and weather increase uncertainty in state estimation and rapid decision-making [50]. A delayed control cycle, stale data, or inconsistency between commands and actuator responses can produce oscillation, overshoot, delayed action, and path deviation [51]. Communication interruption deprives the controller of updated information, while latency allows an obsolete state to continue influencing decisions. Existing evidence supports architectural faults involving disconnection, latency, and desynchronization, but is insufficient by itself to establish mechanisms of bus congestion or cyberattack; those mechanisms are beyond the scope of this section.

The actuation layer includes motors, proportional and hydraulic valves, clutches, steering mechanisms, and seeding, fertilizing, and spraying mechanisms [52,53,54,55,56,57,58,59,60,61,62,63]. Burned actuator coils, overheated drives, stuck valve spools, blocked mechanisms, loose linkages, and failed feedback elements prevent commands from being converted into the specified displacement, speed, or flow [64]. The propagation chain can be expressed as an information anomaly that creates a control deviation, followed by an abnormal physical response and ultimately a loss of operational quality or a safety risk. Position-data drift, for example, may cause a seeder to depart continuously from its target depth; distorted flow feedback may produce spatially nonuniform fertilizer application; and steering actuator lag may cause an autonomous vehicle to leave its planned path. The fault ontology should therefore record the fault layer, affected interface, temporal consistency, physical response, and task consequence, providing a common boundary for the diagnostic objects and labels used below.

### 3.5. Multiple Fault Coupling, Cascading Propagation, and Consequence Classification

Agricultural machine faults can be represented as task-dependent graphs whose nodes are components and whose edges are energy, material, information, or control dependencies. Implementation, material, and task changes alter the graph, so normal dependencies, fault states, temporal order, and triggering conditions should be distinguished; simultaneous abnormalities alone do not establish a cascade.

Propagation analysis separates common cause, statistical association, functional dependence, and true cascade. A cascade requires an initiating event, transmission medium, trigger, and temporal order [65,66,67]. Meango and Ouali showed that multicomponent faults may share causes or interact through degradation [68], while Xia et al. and Lin et al. modeled mechanical, electrical, and information coupling and path-dependent reliability [69,70]. These studies support tracing engine–gearbox–running gear, contamination–hydraulic actuator, and sensor–controller–actuator paths rather than inferring propagation from co-occurrence.

A typical energy chain is torque attenuation caused by engine combustion or abnormal oil supply. Gear meshing impact occurs after the gearbox switches to a low-speed and high-load state. The impact expands the bearing gap, eventually causing walking traction fluctuations. The hydraulic chain can start from oil contamination or pump wear and develop through flow attenuation and pressure pulsation into valve sticking and actuator lag, eventually causing the tool’s operating depth or application rate to deviate. The information chain may start from sensor drift, loss of connection, or time desynchronization, and may be misjudged by the controller to form a command deviation, which may then be transformed into inconsistent actuator response and work trajectory deviation. Xia et al. summarized the propagation of complex electromechanical systems into three types of coupling: mechanical, electrical, and information, and predicted the failure probability [69] between components based on this. Lin et al. evaluate system reliability based on changes in propagation paths and node effectiveness, emphasizing that system consequences depend on fault expansion rather than the status of a single component [70].

Coupled faults may amplify or suppress symptoms: lubrication loss can raise wear and temperature, while cooling loss can accelerate electrical degradation. Consequences should be rated separately for safety, operational quality, downtime, and repair accessibility. Vibration, temperature, pressure, and speed remain symptoms; criticality depends on the propagation path, consequence, and detectability summarized in Table 4.

System consequences should be evaluated along four dimensions: safety, operational quality, downtime, and repair accessibility, rather than treated as four successive severity levels. Safety concerns include steering, braking, load bearing, and emergency control capability; operational quality concerns sowing depth, application rate, coverage, and path accuracy; downtime concerns task interruption and farming window losses; and repair accessibility reflects fault concealment, isolation difficulty, and resource requirements. Critical nodes can be identified jointly from propagation likelihood, consequence severity, and detection difficulty. If observability is retained as an input, its inverse must be used so that easily observed faults do not receive a higher risk score. Table 4 consolidates the preceding fault entities, propagation relationships, and task-failure boundaries by ontology level, representative object, fault state, formation condition, observable evidence, and task consequence.

### 3.6. Other Fault Types

Other faults include loosened or deformed frames and connectors, abnormal sealing or lubrication and cooling, failures of auxiliary or protective devices, and secondary faults caused by incorrect assembly or maintenance. Although these objects are diverse, their formation can usually be traced through load, wear, contamination, thermal, or control pathways. Records should therefore distinguish initiating conditions, physical damage or abnormal state, component or interface failure, observable symptoms, and mission consequences, then assess performance, operational quality, and safety boundaries.

The ontology and boundary definitions in Table 4, together with Section 3.6, provide a consistent basis for defining diagnostic tasks, configuring sensor locations, and interpreting evidence in the following chapters.

## 4. Agricultural Machinery Fault Detection, Localization, Identification, and Trustworthy Diagnosis

### 4.1. Diagnostic Tasks, Sensor Placement, and Multisource Data Collection

Data collection should work backward from the task. Anomaly detection, localization, type identification, and severity estimation require different variables, coverage, bandwidth, synchronization, and labels [71]. Sensor placement should therefore map each fault to sensitive variables, source, location, sampling, synchronization, and data fields, with upstream–downstream and counterevidence coverage across powertrain, hydraulic, control, and implement systems [72,73,74,75].

Sensor placement can be mapped as “Fault–Sensitive Variable–Sensor Source–Installation Location–Sampling and Synchronization–Data Field.” Powertrain faults require correlated measurements of speed, torque, temperature, pressure, and load at upstream and downstream locations; hydraulic faults require simultaneous pressure, flow, oil temperature, and actuation measurements across pumps and valves; electronic control faults require sensor readings, commands, actuator feedback, and controller logs; and implement faults also require implement position, trajectory, and working material state [72,73,74,75]. The aim is not to maximize the number of measurements but to obtain supporting evidence, upstream–downstream comparisons, and plausible counterevidence for each fault hypothesis.

Onboard signals, controller logs, environment, remote sensing, UAV information, and maintenance records provide complementary evidence [76,77,78,79,80,81,82,83]. Barrile et al. used satellite, UAV, navigation, autonomous vehicle, and geographic information to characterize crop context [78], while Rijanto et al. acquired four tractor temperature channels through CAN, stored them locally, uploaded them remotely, and reported no transmission errors at 100 ms [84]. Context data explain field and task variation but do not replace component evidence; acquisition records should retain channel, controller, time, communication, storage, alarm, and mode-change information.

Rijanto et al. developed an embedded monitoring system for a sugarcane-harvesting tractor, treating the machinery as an electromechanical system comprising the tractor, an electrohydraulic actuator, and several electronic control units. The prototype acquired four channels of temperature data through a controller area network, wrote the data to a Secure Digital memory card, and simultaneously uploaded them to an Internet of Things server. No transmission errors occurred at an update interval of 100 ms [84]. This example shows that field acquisition should retain not only measured values but also channel identifiers, source controllers, timestamps, communication status, and storage locations, allowing the same data to be traced across the controller area network, local files, and remote platform. Event logs should likewise record alarms, mode changes, command changes, and communication interruptions so that continuous physical measurements can be aligned with discrete control events.

Multisource records should share asset identity and time base [85]. Zhang et al. linked agricultural digital twins to physical equipment, sensing, models, and virtual representations [86]. This review therefore recommends a compact data contract covering machine, component, implement, task, condition, maintenance version, time, unit, sampling, source, label basis, and quality flags. Low-frequency baselines may be supplemented by event-triggered high-frequency windows, with triggers, synchronization, and fallback behavior specified in Table 5.

Existing collection studies support a strategy of low-frequency baseline monitoring with event-triggered high-frequency sampling. Baseline data describe long-term operating conditions; changes in load, alarms, or command–response inconsistencies trigger faster sampling and retention of pre- and post-event data. Implementation should specify trigger variables and thresholds, capture windows, clock synchronization, and fallback behavior. The resulting data contract should document the diagnostic object, variables, sensor locations, time base, device identity, operating condition tags, quality flags, and traceability. Table 5 maps these requirements to different diagnostic tasks.

### 4.2. Data Quality Control, Preprocessing, and Representation

Diagnostic credibility begins with completeness, synchronization, noise, drift, outliers, and independence between training and test units [87,88,89,90,91]. Lei et al. described diagnosis as an evidence chain from acquisition and feature construction to health state identification [92]. Preprocessing should address a documented defect or signal mechanism: filtering, resampling, alignment, and imputation flags are useful only if impact peaks, modulation, and observation provenance remain intact [93].

Lei et al. described intelligent fault diagnosis as a continuous evidence chain linking data acquisition, engineered feature extraction and selection, health state identification, and the later transition to end-to-end and transfer learning [92]. Preprocessing should therefore respond to specific data defects and signal-generation mechanisms rather than follow a fixed pipeline [93]. Strong random noise may justify filtering; uneven sampling or inconsistent source frequencies require resampling and temporal alignment. For missing data segments, both missing positions and imputation flags should be retained so that estimates are not mistaken for observations. Denoising must also be checked for attenuation of impact peaks and fault modulation; a smoother signal may contain less diagnostic evidence.

**Figure 7:**
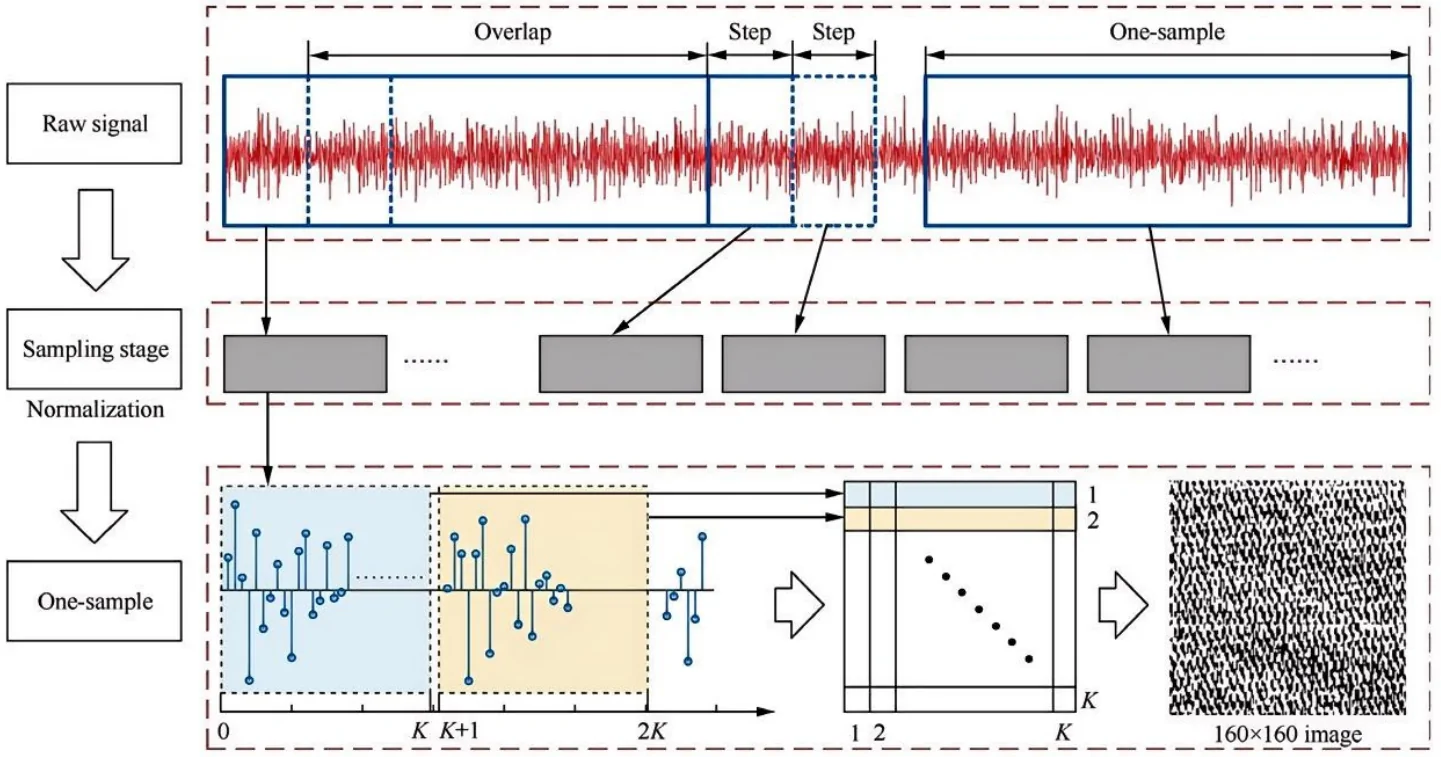
The conversion process of time series signals to two-dimensional images. Source: Reproduced without modification from Tang et al. [95] under CC BY 4.0.

Variable speed moves fault frequencies and can broaden fixed-frequency spectra. Liu, Cui, and Wang reviewed order tracking, cyclic correlation, generalized demodulation, and frequency–frequency analysis; order representations are useful when speed is reliable, whereas rapid or uncertain speed changes favor time-frequency analysis [94]. Tang, Yuan, and Zhu reviewed Fourier, wavelet, S-transform, cyclic-spectrum, and augmentation inputs [95]. Figure 7 illustrates two-dimensional mapping, whose length, overlap, normalization, and order should preserve temporal and physical relations.

A representation should balance separability, operating condition robustness, physical meaning, and deployment cost. Validation should be separated by machine, field, task, or season. Residuals estimated from healthy response and load variables may be useful, but completeness, synchronization, signal-to-noise ratio, drift, outliers, and composite score weights require target machine calibration rather than universal thresholds.

### 4.3. Fault Reasoning Based on Mechanisms, Expert Knowledge, and Knowledge Graphs

Knowledge-driven diagnosis defines objects, semantics, causal constraints, and reasoning boundaries. Machine, implement, task, component, fault, symptom, and cause can be linked through structural, causal, manifestation, and exclusion relations. Expert and mechanistic rules constrain candidate faults—for example, by transmission path or pressure-flow response—but missing premises should return candidates or abstention rather than a forced class [96].

Expert knowledge can express stable causal constraints. Gear mesh faults should be consistent with the transmission path; hydraulic internal leakage usually reduces both pressure and actuation capability; and an isolated temperature rise does not identify a unique fault [96]. Mechanistic models estimate normal responses from mass, energy, or motion relationships, and deviations between observations and model output provide diagnostic evidence. Rules may map the residual direction, operating conditions, and symptom combinations to candidate faults, but their validity depends on complete premises. When key evidence is missing, the system should return an unknown state or a candidate set rather than force a unique class.

Ontologies unify concepts; knowledge graphs instantiate equipment, faults, and evidence. Xu et al. combined a loader ontology, case reasoning, and semantic rules [97]. Cai et al. used layered structure and relation paths in 24 steel-motor cases and reported 91.1% accuracy with missing inputs [98]. The result supports reasoning with incomplete observations but is not an agricultural performance estimate; machine-specific structures and criteria must remain explicit.

Rule reasoning leads to conclusions along predefined conditions. The advantage is that the path is clear, but the limitation is insufficient coverage when facing missing symptoms and complex relationships. Relational path reasoning searches candidate chains along “symptom–component–fault–cause” or “upstream fault–interface–downstream symptom” and uses multiple relationships to jointly constrain the results. Cai et al. established a multilayer equipment fault knowledge graph, organized characteristics, system status, and equipment structure into different levels, and then used relationship paths to locate the fault source and infer the cause. Their study set up 24 fault cases with complete symptoms and achieved a diagnosis accuracy of 91.1% under the condition of missing input features, indicating that the structural relationship in the graph can supplement incomplete observations [98]. However, this value comes from the steel plant motor object and cannot be directly regarded as the expected performance on agricultural machinery.

Wang, Taal, and Fink combined expert fault impact rules, synthetic bearing signals, and domain adaptation across laboratory and wind-turbine bearings [99]. For agricultural transfer, knowledge should constrain acceptable fault form and migration direction, while model, speed, load, implement, data quality, conflicts, counterevidence, and rule version remain traceable. Confidence updating and abstention still require target machine calibration.

Because evidence sources may conflict, inference output should preserve each source, its direction of support, and its reliability. If model residuals support a fault that expert rules reject, the applicable operating conditions and rule versions should be checked; if sensor evidence conflicts, its weight should reflect the data quality fields in Section 4.2. The proposed output includes candidate faults, locations, supporting paths, conflicting evidence, and a minimum counterevidence set. Reliable counterevidence should inform the confidence update and abstention process in Section 4.5. This is a traceable reasoning framework; its confidence update function still requires calibration on the target machine.

### 4.4. Data-Driven and Mechanism–Data Fusion Diagnosis

**Figure 8:**
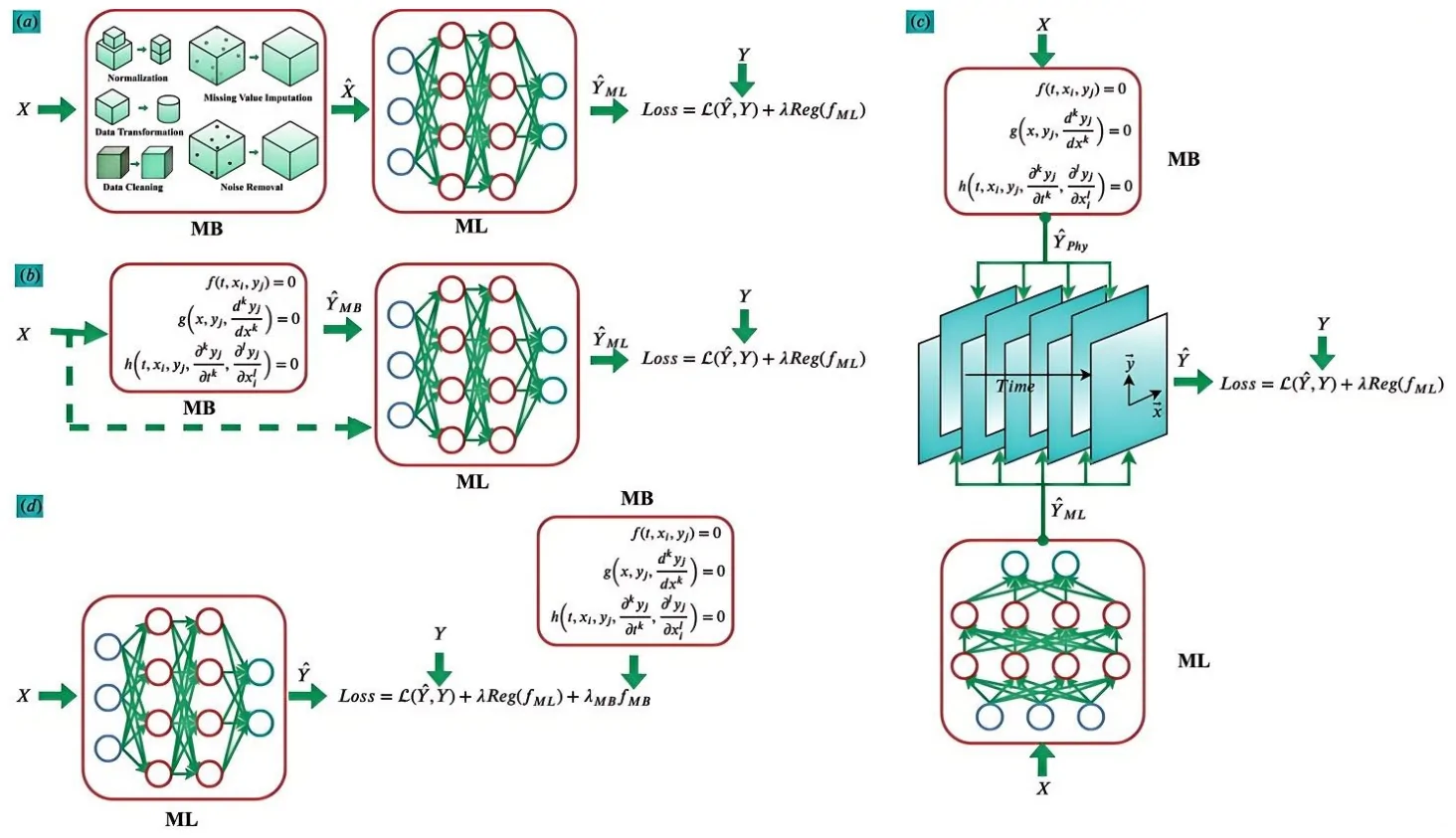
Types of hybrid physics-guided machine learning models: (**a**) physics-based preprocessing; (**b**) physics-based network architectures; (**c**) spatiotemporal physics-based network integration; and (**d**) physics-based regularization. Source: Reproduced without modification from Rai and Sahu [104] under CC BY 4.0.

Method choice depends on task, independent data, mechanism availability, operating condition coverage, and resource budget. Engineered features provide interpretable baselines; deep networks learn complex representations; and hybrid methods introduce physics through inputs, features, architecture, losses, residuals, or decisions [100,101,102,103,104,105]. Tama et al.’s review of 59 vibration-based studies highlighted label scarcity, transfer, interpretability, and field drift [102]; Rai and Sahu further recommended joint evaluation of accuracy, cost, interpretability, and generalizability [104]. Figure 8 summarizes the fusion routes, but inaccurate constraints can cause negative transfer.

Tama et al. reviewed 59 vibration-based deep learning studies and identified persistent limitations in labels, operating condition transfer, interpretability, and field distribution shift [102]. Rai and Sahu placed hybrid cyber–physical models on a continuum from model-driven to coupled learning and recommended evaluating accuracy, computational cost, interpretability, and generalizability together [104]. For agricultural machinery, the reviewed evidence supports mechanism-guided input or feature coupling when reliable variables exist, and constraint or decision fusion when governing relations and applicability conditions can be audited; inaccurate constraints may otherwise cause negative transfer. Figure 8 summarizes these routes.

González-Baldizón et al.’s control-aware LQC-CLSTM-AE converts command-response inconsistency into a conditional reconstruction residual and retains adaptive-threshold states [105]. For the diagnostic passport, the useful contribution is an auditable tuple of command, response, residual, threshold version, signature, timestamp, confidence, conditions, and conflicts. Fusion is valuable when it improves both performance and traceability; disagreement between data and mechanism models should be reported with uncertainty rather than forced into one class.

Fusion output should report fault type, location, severity, applicable operating conditions, data sources, mechanism constraints, computational cost, uncertainty, and abstention criteria. Drawing on the auditable output structure reported by González-Baldizón et al. [105], controller command, monitored response, conditional residual, threshold version and state, residual signature, and detection timestamp should be retained together for control-aware residual diagnosis. Conflicts between data and mechanism models should be returned with their uncertainty rather than resolved by forced classification. Data-driven components supply flexible representations, while mechanistic knowledge supplies boundary constraints; fusion is useful when both diagnostic performance and evidential traceability improve.

Table 6 maps diagnostic tasks to system links, detection methods, data processing, and fault representations. The mapping emphasizes that tasks require different evidence and outputs: anomaly detection does not replace fault localization or type identification, and a fault label alone cannot support an operations-and-maintenance decision without operating conditions, credibility, and an applicability domain.

### 4.5. Evaluation of Diagnostic Performance, Trustworthiness, and Deployment Cost

Current state diagnosis evaluation should separate anomaly detection, localization, type, and severity and assign asymmetric costs to false negatives, false positives, and abstention. Salinas-Camus, Goebel, and Eleftheroglou organized evaluation around uncertainty, robustness, interpretability, and feasibility [106]. Testing by new machine, field, season, and unknown conditions should accompany sensitivity, specificity, macro-recall, calibration error, and intervals [107], especially for safety-critical faults.

Trustworthiness requires calibrated probabilities and explicit abstention. Data quality, case similarity, mechanism residuals, and model disagreement may update confidence; missingness, drift, or out-of-domain input should lower it, while credible safety evidence remains visible for review. Robustness tests should cover load, impact, dust noise, channel loss, desynchronization, and machine replacement [108,109,110]. Xu et al.’s physics-informed probabilistic network supports joint consistency and uncertainty checks [111] without establishing agricultural field performance.

Trustworthiness also requires calibrated probabilities. Because high-confidence errors are more dangerous than low-confidence errors, output probabilities should be evaluated with reliability diagrams, expected calibration error, and confidence interval coverage. Data quality scores, case similarity, mechanism residuals, and model disagreement may jointly update confidence. Extensive missing observations, clear drift, or an input outside the training domain should reduce confidence and trigger abstention; strong physical evidence of a safety-critical fault should remain flagged for manual review even when classification probability is modest. Abstention is an explicit recognition of evidential limits, not a model failure.

Interpretability should identify evidence, its physical referent, applicable conditions, alternatives, and counterevidence. Moosavi et al. distinguished transparent, post hoc, and interactive explanations [112]. These requirements, together with latency, deployment cost, applicability, and abstention, feed the diagnostic passport in Table 7; they are proposed delivery fields rather than proof that the schema has been validated end-to-end.

**Figure 9:**
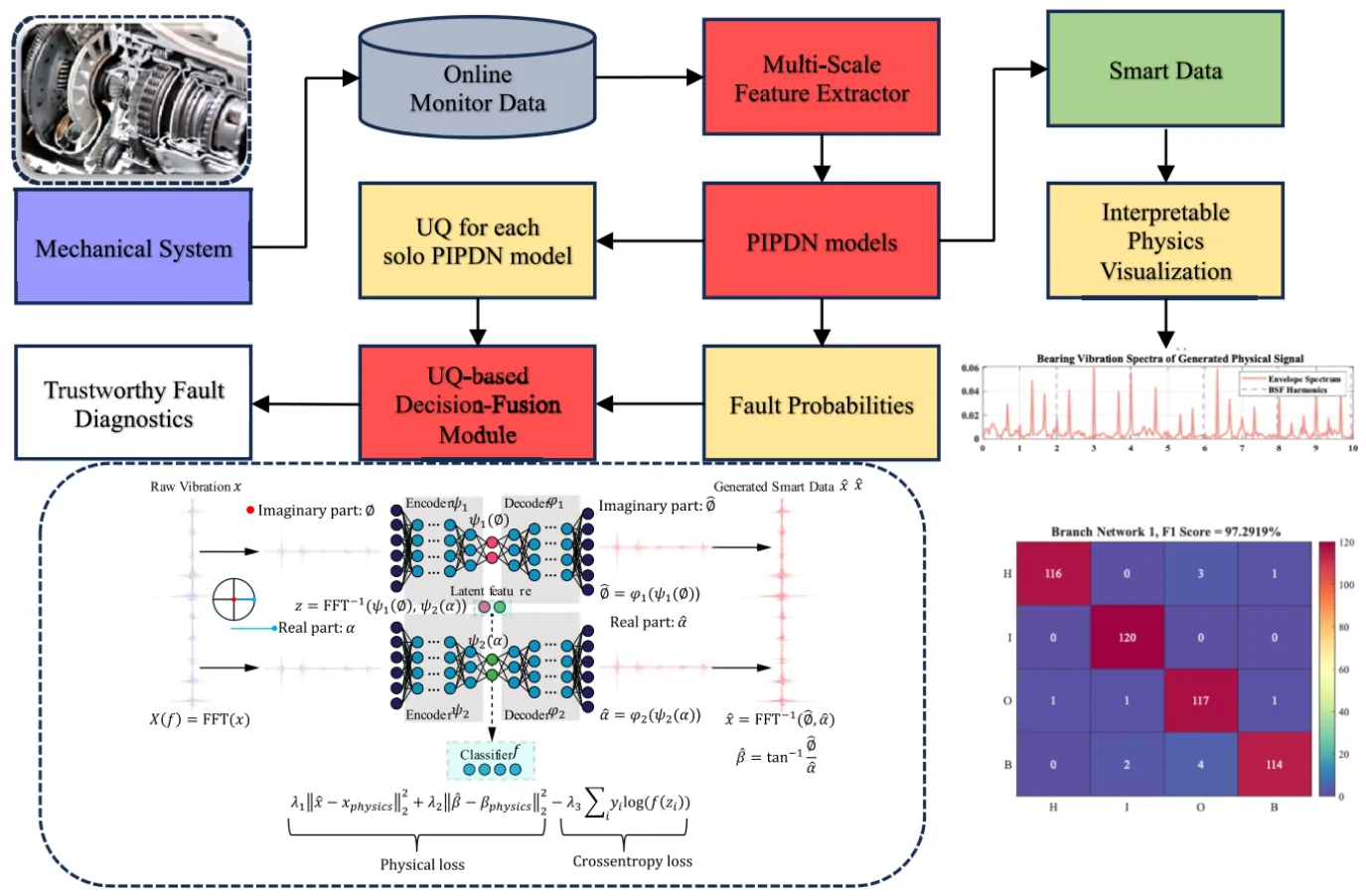
Fault diagnosis framework based on uncertainty quantification and decision fusion. Source: Reproduced without modification from Xu et al. [111] under CC BY 4.0.

Figure 9 shows the relationship between multi-scale feature extraction, probabilistic diagnosis, uncertainty quantification, and decision fusion. For agricultural machinery, the key to this framework is to use uncertainty and physical consistency checks to determine fusion, review, or abstention, rather than simply adding diagnostic branches.

Interpretability evaluation should go beyond displaying important features. Moosavi et al. classified explainable artificial intelligence for industrial cyber–physical systems into model transparency, post hoc explanations, and user-oriented interactive explanations, emphasizing the evidence used, its physical referent, and the conditions under which a conclusion holds [112]. Each diagnostic result should therefore report evidence sources, key variables, fault location, reasoning path, counterconditions, and applicable operating conditions. The explanation should also identify plausible alternatives—for example, whether high load or ambient temperature accounts for an observed temperature rise.

This review organizes diagnostic output into a theoretical diagnostic passport containing identity, operating conditions, fault type and location, severity, probability or interval, calibration, evidence strength, applicability domain, latency, resource budget, conflicts, and abstention reasons. The passport inherits traceability and uncertainty reporting from PHM and condition-based maintenance and adapts them to machine–implement–task identity; control-aware diagnosis can additionally retain synchronized commands, responses, residuals, threshold history, signatures, timestamps, and confidence [105]. The proposed field set is not an industry standard and has not been validated as a complete schema. Table 7 therefore labels fields as proposed minima or recommendations and states review or abstention conditions rather than universal commands.

The diagnostic passport defines the applicability and delivery boundaries of current state conclusions. Section 5 extends this evidence to fault evolution, prognostics, warning, and proactive risk control.

## 5. Agricultural Machinery Fault Prognostics, Early Warning, and Proactive Risk Control

### 5.1. Health Indices, Degradation Stages, and Early Warning

**Figure 10:**
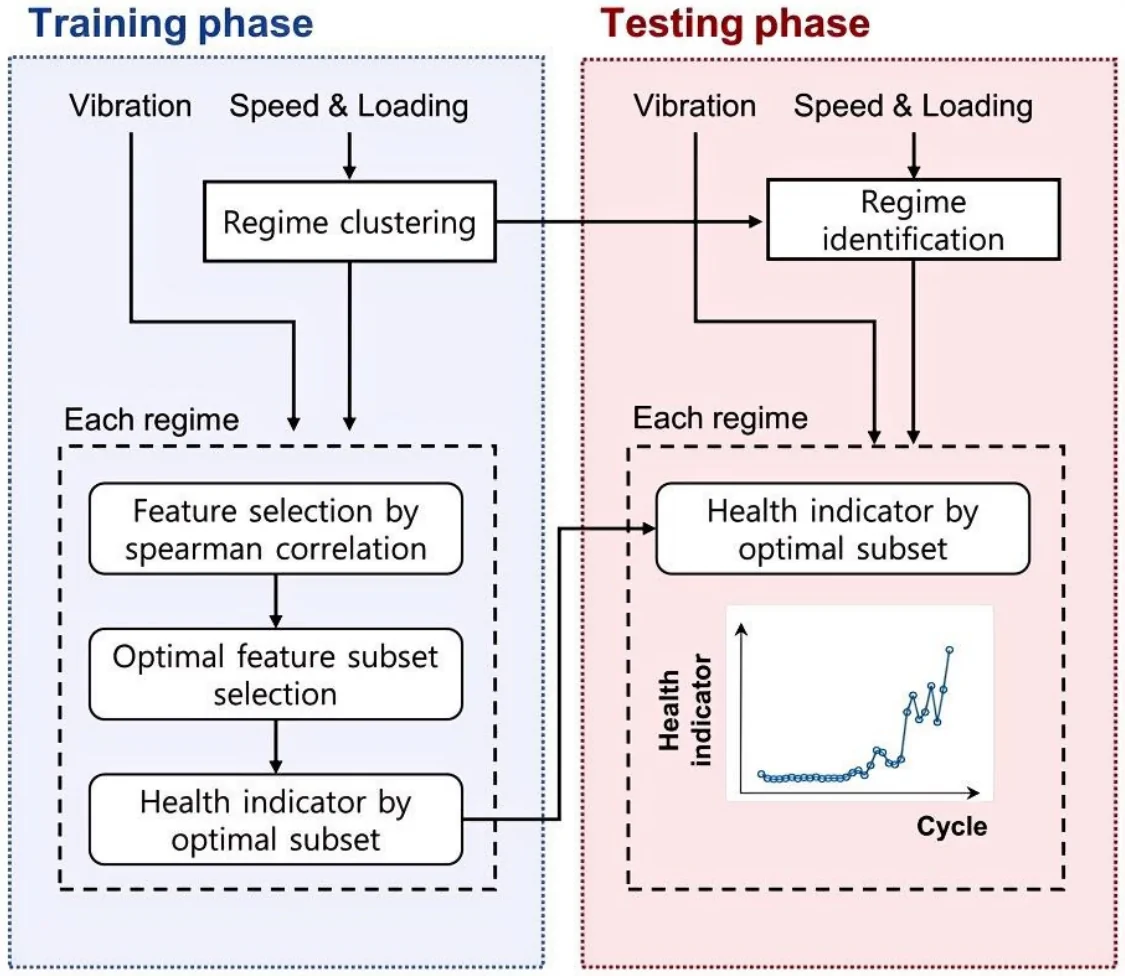
Construction and application process of health indicators under variable operating conditions. Source: Reproduced without modification from Kim et al. [116] under CC BY 4.0.

A health index should separate equipment health from operational quality and remain interpretable under changing tasks. Wang, Tsui, and Miao classified signal-processing, mechanism, and learning indices [113,114,115]; Kim et al. first identified the operating state and then applied state-specific features and indices [116]. Speed, load, pressure, vibration, energy, and task quality variables may be corrected within comparable task profiles, while component contributions remain visible and thresholds are calibrated on the target machine. The process is shown in Figure 10.

Healthy and failure anchors should follow the performance, quality, and safety boundaries in Section 3. Stage changes require threshold magnitude, duration, and multivariable consistency. Increased speed, traction, or hydraulic load can otherwise create pseudo-degradation; condition partitioning and within-profile comparison are therefore preferable to one static threshold [116].

Changing operating conditions are the most likely cause of pseudo-degradation in a health index. Increased rotational speed, increased traction resistance, or sudden changes in hydraulic load will simultaneously change vibration, temperature, and pressure, and the uncorrected index may rise even if the components are not damaged. For variable speed and variable load bearings, Kim et al. proposed to first divide the operating condition area, and then select features in each area and use Mahalanobis distance to construct a health index to reduce the interference of operating condition changes on health evaluation [116]. This approach is directly relevant to agricultural machinery: task profiles such as idle speed, transportation, light load, heavy load, and impact operation should be identified first, and then the degree of deviation within the same profile should be compared, instead of using a static threshold to cover the entire operation process.

With few failures, normal sequences can learn a baseline. de Pater and Mitici reported a 97% increase in health index monotonicity after adding operating conditions in a turbofan study [117]. This supports using load, speed, slope, soil resistance, and machine state as context, not transferring its thresholds to agriculture. Early warnings should use hysteresis and persistence, update baselines only in confirmed normal windows, and separate current stage warnings from probabilistic life prediction.

Early warning should adopt dynamic criteria with hysteresis. When the index exceeds the early degradation boundary for the first time, only a candidate early warning can be triggered. The early warning level will only be upgraded when the boundary remains crossed for a specified duration, and the equipment health component is in the same direction as the operational quality component. If the index drops quickly after load switching, it should be judged as an operating condition disturbance. Individual baselines can be updated online from healthy segments of the same device, but this review recommends limiting updates to a confirmed normal window to avoid absorbing true degradation into the new baseline. Cross-task profile verification should check whether the warning precedes functional failure, is triggered repeatedly due to frequent gear changes and implement hookups, and whether the index is comparable between different equipment. This section only outputs the current health level, degradation stage, and early warning signals with persistent conditions, leaving probabilistic life prediction to 5.2.

### 5.2. Degradation Forecasting and Remaining Useful Life Estimation

Prognostic studies should define the object, prediction origin, task profile, failure threshold, update frequency, and future load assumption. Remaining useful life is the distribution of first passage across a functional, safety, or quality boundary, not an isolated value [118,119]. Elattar, Elminir, and Riad grouped methods as physics-based, statistical, and data-driven [120]; their comparability depends on common origins, thresholds, conditions, and horizons.

Elattar, Elminir, and Riad described prognostics as condition monitoring, degradation characterization, future state inference, and remaining useful life estimation and grouped methods as physics-based, statistical, or data-driven [120]. Physics-based methods infer future states from fatigue, wear, crack-growth, or capacity-loss laws and are interpretable when material properties and load histories are reliable; changing soil resistance, impact, and implements often make these boundary conditions incomplete. Statistical methods use stochastic processes, state-space models, or survival distributions to represent individual variation and censored data but depend on distributional assumptions. Deep sequence models capture nonlinear and multistage degradation but require representative full-life data.

Models should allow stage transitions, re-estimate parameters after change, and treat preventive replacement or incomplete observation as right censoring [121]. Zhang et al. linked multimodal data to health representation and remaining useful life regression but emphasized data volume, labels, and condition coverage [122]. Agricultural validation should distinguish load switching from degradation and separate machines, fields, and seasons.

Zhang et al. reported that deep learning can link vibration, images, time series, and structured data to health representation and remaining useful life regression, but its performance depends on data volume, label quality, and coverage of operating conditions [122]. Agricultural machinery inputs should include speed, load, environment, machine state, and operational events as well as the health index. Models must distinguish ordinary load switching from changes in degradation rate and should be validated on independent machines, fields, and seasons. Deep, mechanistic, and statistical models should be compared at the same prediction origin, failure threshold, and assumed future conditions rather than by one average error alone.

Forecasts should separate process randomness from model uncertainty and report interval coverage, width, lead time, and early or late error. Liu, Shang, Ouyang, and Widanage combined decomposition, Gaussian processes, and long short-term memory to produce battery trends and uncertainty intervals [123]; this supports the architecture, not the transfer of battery degradation to agricultural life. Updates require quality and applicability checks, reset affected-component baselines after repair, and retain history. Results should report both remaining time and remaining area, cycles, or task batches under stated loads.

Online updates should follow new diagnostic evidence, material changes in operating conditions, or maintenance events. New observations should first pass data quality and applicability checks before updating health, degradation rate, and the posterior life distribution; changes in interval width and risk probability should be recorded as information gain. Part replacement or machine adjustment breaks the original trajectory: the affected component baseline should be reset while system history remains traceable. Evaluation should report early and late prediction error, interval coverage and width, lead time, and out-of-domain abstention in addition to root-mean-square error, so that average performance does not conceal dangerous errors near failure.

This review recommends reporting both remaining effective operating hours and the workload that can be completed under the current load. The latter may be expressed as remaining area, cycles, or task batches and should be tied to current load and seasonal conditions. Each estimate should state the prediction origin, failure definition, confidence interval, applicable operating conditions, and update rule so that maintenance timing and preventive actions follow an explicit future-risk distribution.

### 5.3. Credibility of Small-Sample, Real-World-Fault, and Prognostic Evidence

Credible prognosis depends on time-extrapolative data, including equipment source, fault generation, conditions, labels, collection time, and sample relationships [124]. Neupane and Seok summarized the CWRU benchmark: 12/48 kHz sampling, 0–3 hp, and 1720–1797 r/min [125]. It supports controlled method comparison, not complete machine prediction under mud, impact, implement changes, or natural degradation.

Neupane and Seok reported that the Case Western Reserve University bearing dataset has become an important comparison benchmark for deep learning fault diagnosis because of its openness, clear structure, and wide use. The data include the vibration of the drive end, fan end, and part of the base. The sampling frequency is 12 kHz and 48 kHz. The motor load covers 0 to 3 horsepower, and the rotation speed range is 1720 to 1797 r/min [125]. These data are useful for comparing methods, but their fixed test rigs, limited loads, and preset bearing failures are not representative of the muddy water contamination, traction shock, implement replacement, and long-term natural degradation found in agricultural machinery. The results thus obtained can only prove the feasibility of the model on a controlled bearing basis and cannot directly infer the field prediction capabilities of the complete machine, hydraulic system, or working implement.

**Figure 11:**
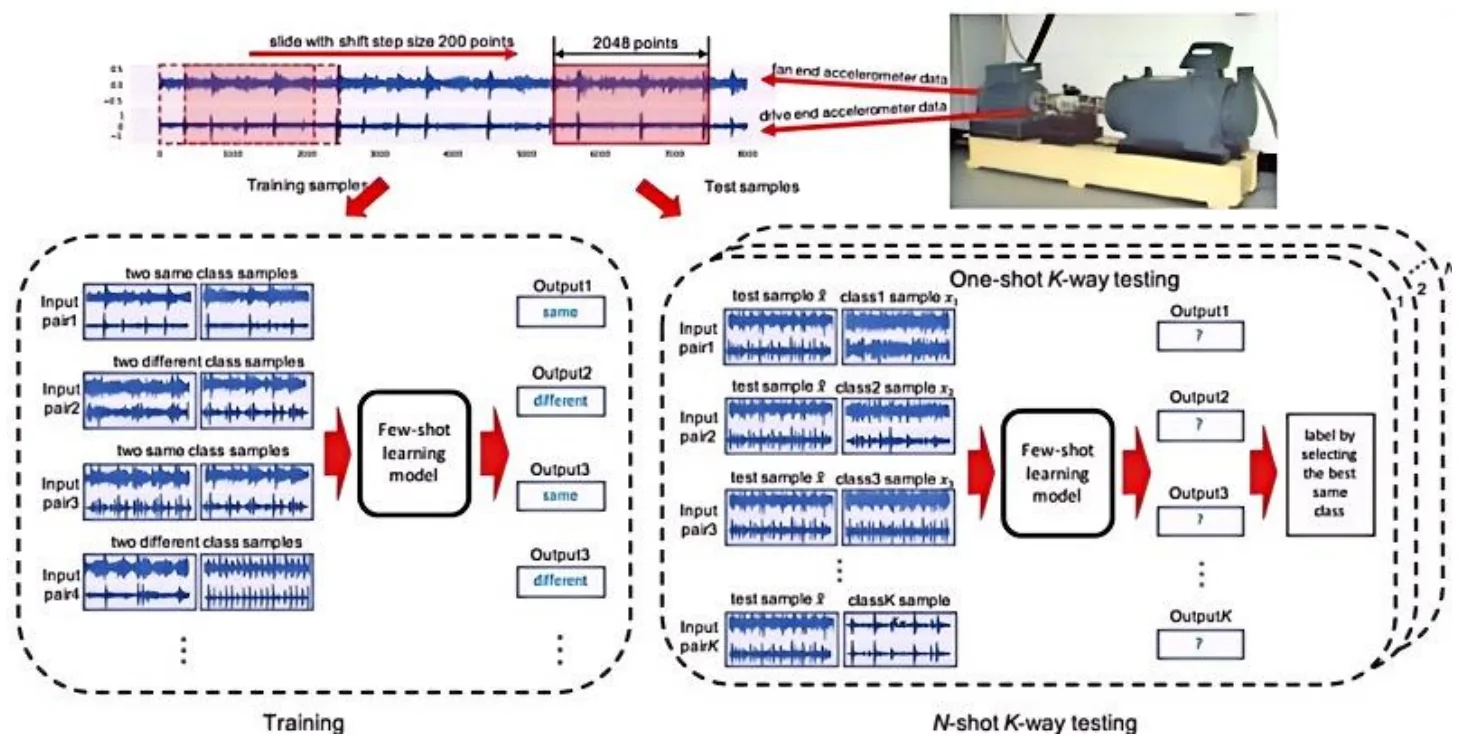
Bearing fault diagnosis process based on few-shot learning. Source: Reproduced without modification from Zhang et al. [127] under CC BY 4.0.

Field-fault scarcity reflects early intervention, long degradation, delayed labels, ambiguity, and novel faults [126]. Zhang et al.’s paired-sample method tested new fault types, conditions, and noise [127], but sliced windows are not independent events. Zheng et al. showed that machine, load, speed, and environment produce domain shift [128]. Validation should therefore separate machine, time, field, and season and reject random splits as evidence of cross-domain prognosis. The process is shown in Figure 11.

Domain shift further weakens prognostic evidence. Zheng et al. noted that the assumption of identically distributed training and application data fails when machine structure, load, speed, or environment changes. Transfer learning can use a related source domain, but differences in label sets, feature distributions, and task definitions must be explicit [128]. Validation should therefore be separated by machine, time, field, and season. New machine testing examines structural variation, temporal separation prevents leakage from adjacent slices, new-field testing examines soil and crop effects, and new-season testing examines environmental and duty-cycle variation. Random splitting alone cannot support cross-domain claims.

Agricultural machinery model migration requires distinguishing machine models, sensors, fields, seasons, loads, and fault distribution changes. Model shift changes the structure and fault boundaries, sensor shift changes the range, installation, and sampling methods, field and season shift changes the environment and work objects, load shift changes the normal signal and degradation rate, and fault distribution shift may introduce unknown categories. Diagnostic domain adaptation research can provide a methodological basis for the representation transfer of predictive models, but without verification of future time windows and degradation trajectories, predictive generalization cannot be directly proven.

Transfer is defensible only when mechanism, structure, labels, and task remain comparable. Li et al. aligned multilayer bearing features across domains [129]; parameter adaptation suits similar structures with new load ranges [130]; and Lin et al. used heterogeneous acceleration and acoustic tasks for few-sample adaptation [131]. Forced alignment should be rejected when structure, sensor location, or fault meaning changes, and target evidence should count independent machines and runs rather than adjacent slices.

After adaptation, probabilities require recalibration, unseen machines and tasks remain final tests, and unknown or low-confidence inputs trigger abstention or a minimum safe fallback. The prognostic evidence ledger should record independent machines, complete trajectories, censoring, label delay or conflict, domain differences, slice adjacency, interval coverage, warning lead time, and failed predictions. Only prospective sequences after model fixation directly test lead time and coverage.

After few-shot adaptation, output probabilities must be recalibrated, and genuinely unseen machine types, implements, and tasks should be retained as the final test domains. This review proposes five technical steps: generalizability assessment, unknown state detection, confidence calibration, safe abstention, and fallback modeling. The target domain is first assessed against the structural and operating condition ranges; unknown classes and substantial distribution shifts are then identified; and a small set of trusted labels is used to calibrate probabilities. Low-confidence or out-of-domain samples trigger abstention, and if the primary model is unavailable, the system switches to a minimum safe model based on critical physical variables. This process still requires validation against degradation sequences and warning lead times and cannot be established from fault-classification accuracy alone.

The prediction research follows the four-level evidence system in Section 2.5 and establishes a prognostic evidence ledger to record the number of independent devices, the number of complete degradation trajectories, category imbalance, label delay, label conflict, unknown samples, inter-domain differences, slice neighbor relationships, and prediction interval coverage. Controlled fault data mainly support the basic correlation between features and fault tags; single-machine field data are used to test the interpretability of natural operating conditions; across-machine, across-field, or across-season data are used to evaluate extrapolation stability; only forward-looking field sequences after the model is fixed can directly test the prediction lead time and interval coverage. Upgrading evidence requires not only increasing the number of samples, but also improving the independence of equipment, operating conditions, and fault formation methods. Failed predictions, false positives, false negatives, abstentions, and unconfirmed samples should be retained along with successful records.

### 5.4. Edge Warning, Risk Thresholds, and the Closed Loop for Proactive Risk Control

Edge warning is constrained by computation, memory, communication, and energy [132]. In the motor platform studied by de las Morenas et al., an Arduino Uno used a 16 MHz microcontroller; the decision tree occupied 6924 B of program space and 4341 B of dynamic memory, whereas the support vector machine occupied 40,294 B of program space [133]. The result supports jointly reporting latency, memory, and energy, but it does not establish whole-machine agricultural deployment performance.

In a cloud-edge closed loop, the cloud can perform multi-machine training, analyze domain shift, and distribute audited model updates. Edge devices provide continuous inference, unknown state detection, low-latency warning, and operation during network outages, while field systems return false alarms, abstentions, operating conditions, and subsequent confirmation outcomes [134,135,136]. Each model update should record its version, target machine model, and calibration data, and should remain reversible [137]. This architecture defines the operating conditions for prognostics and early warning; it does not itself authorize preventive action. Intervention remains subject to the risk-based decision process described below.

Preventive control requires a decision context that combines health, future risk, evidence quality, remaining field work, weather, personnel, parts, and downtime [138]. Pinciroli, Baraldi, and Zio synthesized maintenance optimization across asset health, operating profiles, production needs, inventory, and uncertainty and reported maintenance expenditure of about 15% of production cost in food-related industries [139]. This figure motivates timing decisions but is not an agricultural cost parameter.

Risk authorization is subordinate to the standards and law applicable to the product and jurisdiction. ISO 12100 provides general machinery risk-assessment and risk-reduction principles, while ISO 25119-1 addresses the design and development of safety-related parts of control systems for agricultural and forestry machinery [140,141]. Regulation (EU) No 167/2013, Regulation (EU) 2023/1230, and 29 CFR 1928.57 impose distinct approval, machinery safety, guarding, and servicing duties where applicable [142,143,144]. Standards provide normative engineering requirements, whereas statutes and regulations create legally mandatory duties within their scope; these instruments are neither globally interchangeable nor replaceable by model confidence, economic loss, or farming urgency.

This review recommends adapting warning thresholds to the task, consequence severity, evidence credibility, and remaining work [145,146,147]. Continued operation, derating, inspection, and planned maintenance can be compared within a rolling window, but a threshold change cannot override an applicable safety-function limit or legal safeguard. Thus, dynamic thresholds govern risk-informed scheduling, whereas mandatory safety boundaries remain fixed within their applicable scope.

When several machines share technicians and spare parts, machine-level optima may conflict with fleet-level performance. Ruiz Rodríguez et al. used multiagent deep reinforcement learning to coordinate parallel machines and technicians with different skills and reported about 75% improvement over conventional corrective and preventive strategies in their test environment [148]. Because this evidence concerns industrial parallel equipment, it should not be used directly as an agricultural fleet benefit estimate. An agricultural fleet model could instead treat each machine as a decision node and coordinate inspection windows, spare parts placement, and technician arrival using failure probability, remaining workload, and field-task priority, while preserving human authorization.

**Figure 12:**
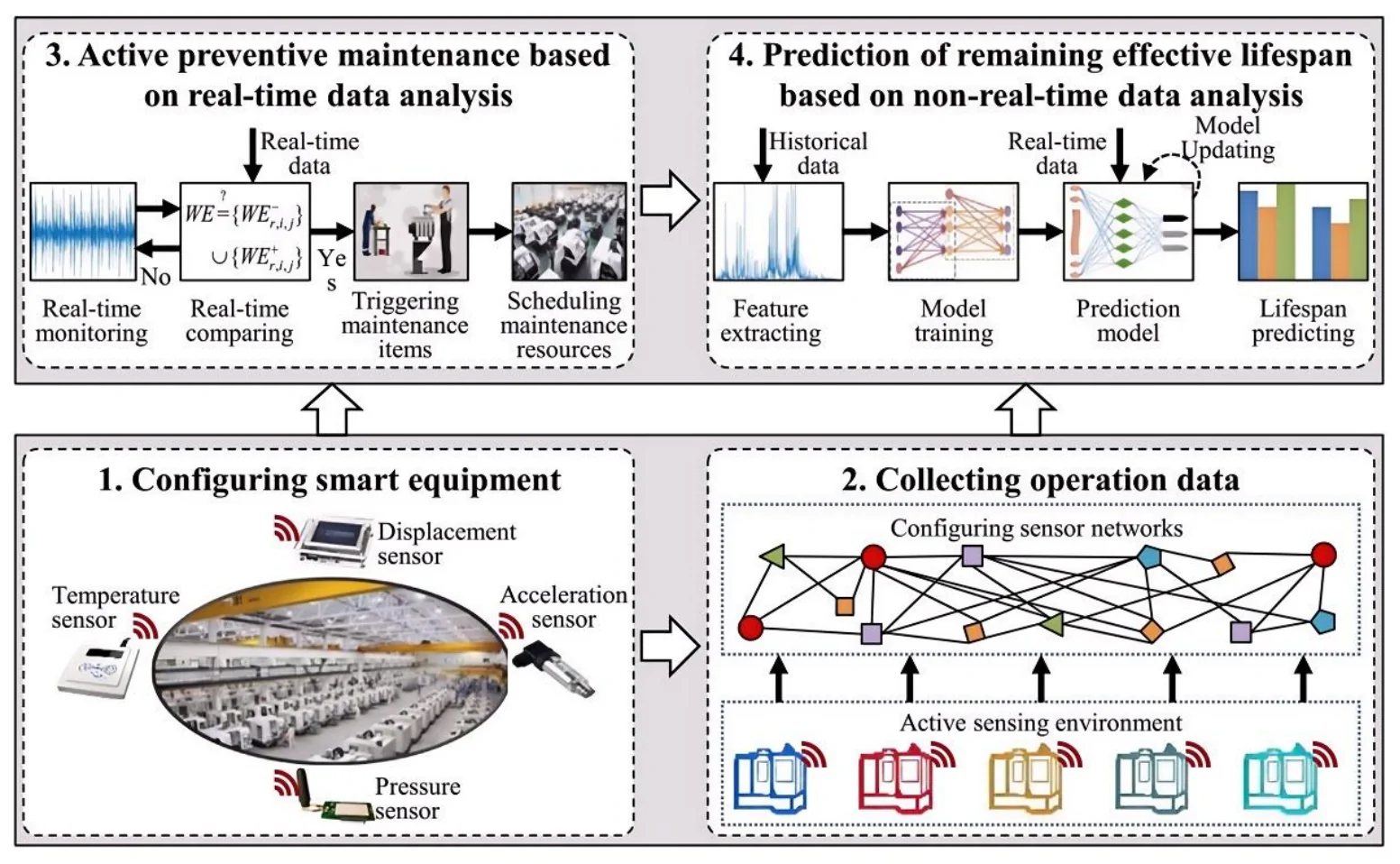
Active preventive maintenance architecture based on real-time monitoring and life prediction. Source: Reproduced without modification from Wang et al. [147] under CC BY 4.0.

Wang et al. reported a product–service–system model that collects operating data, estimates remaining capacity, and stages service resources [149]. For agricultural machinery, this review adapts the logic to pre-position parts, schedule qualified personnel, and insert evidence, risk, and human-authorization checks between a warning and action. Figure 12 illustrates the source architecture; it does not validate automatic derating, shutdown, or parameter changes for agricultural fleets. Zhu et al. reviewed artificial intelligence applications in agricultural equipment across perception, decision, control, and operations and identified data quality, robustness, and deployment constraints [150].

Digital twins can provide a continuous asset identity linking physical equipment, models, data, warnings, resources, execution, and feedback [151,152,153]. Zhong et al. emphasized cyclic state-maintenance mapping, while Fu et al. emphasized lifecycle continuity. Elahi et al.’s review of 42 AI studies found potential benefits and persistent legacy and data-integration barriers [153]. For this review, the relevant function is traceable state, model version, threshold, and action outcome—not visualization or a claim of validated agricultural closure.

Elahi et al. reviewed 42 artificial intelligence studies across design, manufacturing, maintenance, and recycling and concluded that lifecycle knowledge integration can improve decisions, quality, and cost, while legacy system compatibility and data integration remain barriers [153]. A digital twin should therefore do more than visualize status: it should retain state changes, prediction evidence, threshold versions, and action outcomes so that each warning remains traceable and revisable.

Four loops remain distinct: plant control, diagnostic evidence generation, risk-authorized action, and maintenance recovery. Control-aware residual diagnosis and threshold states provide evidence for the first interface [105], while the proposed risk evolution trajectory records health, exposure, future load, consequence, uncertainty, threshold state, and candidate action. These links inherit methods from separate studies and are resynthesized for agricultural mission profiles; no included study prospectively validated the four loops on the same machine. Low-confidence, out-of-domain, or conflicting predictions are therefore routed to review rather than treated as evidence for automatic action.

## 6. Maintenance Decisions, Repair, and Post-Fault Recovery

### 6.1. Fault Confirmation, Isolation, Safe Shutdown, and Maintenance Decisions

Within the proposed framework, entry into maintenance begins when confirmed evidence and risk justify isolation, recovery, or manual intervention. The diagnostic-passport version should be frozen as the review record. Model output is treated as decision input, not as a directly executable repair order; low confidence, compound faults, or contradictory upstream and downstream evidence prompt broader review. These are review-derived workflow requirements, not findings from an end-to-end field trial.

**Figure 13:**
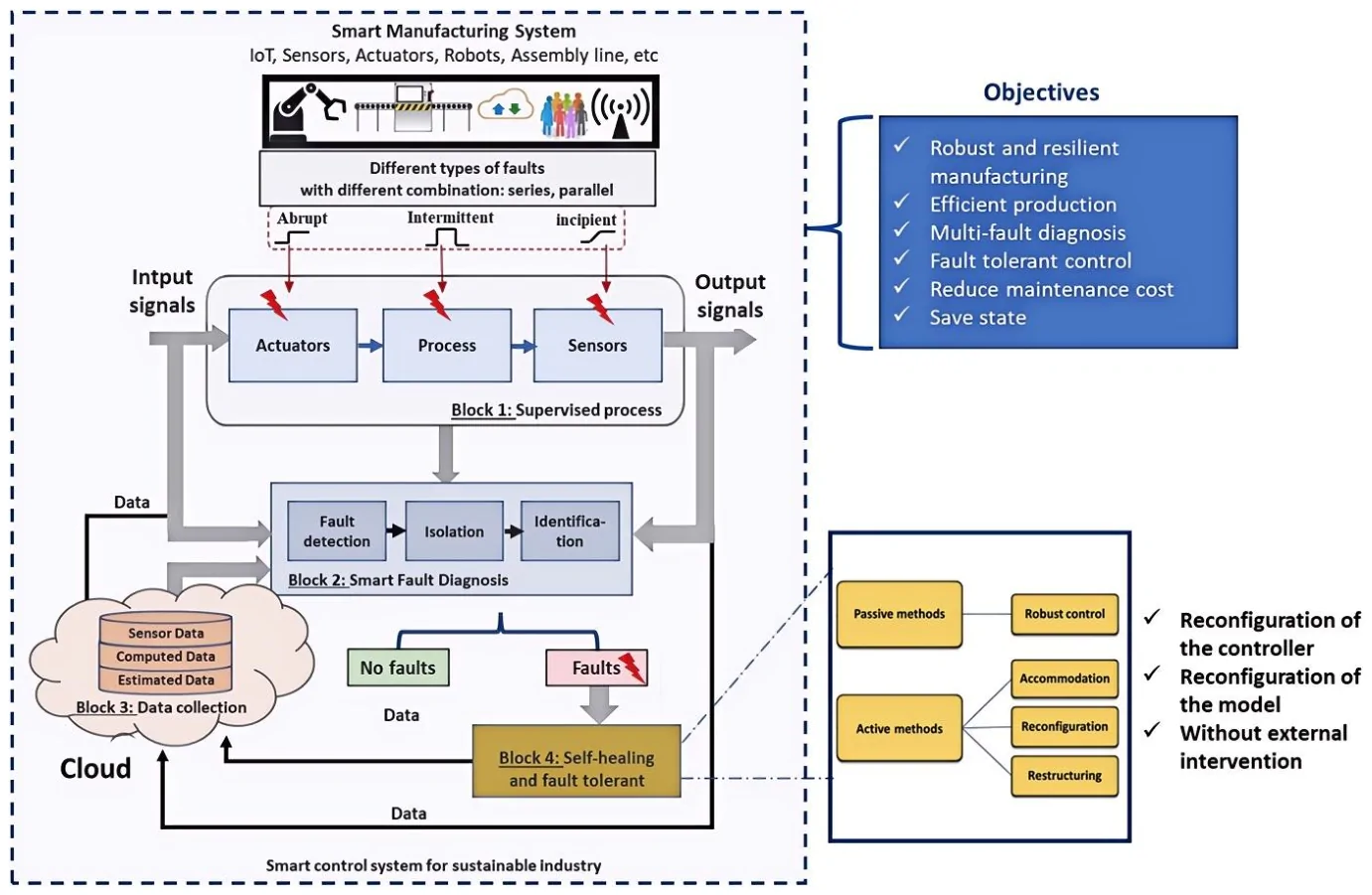
Fault classification and treatment process based on risk priority number. Source: Reproduced without modification from Aldrini et al. [155] under CC BY 4.0.

Sader, Husti, and Daróczi used 1532 faults recorded over one year to construct failure-mode-and-effects data and risk-priority numbers [154]. The finding supports structured ranking, but identical products of severity, occurrence, and detectability can conceal different safety consequences. This review therefore recommends a highest-consequence override for braking, steering, load-bearing, high-voltage, and autonomous control faults. Figure 13 shows the enterprise routing structure reported by Aldrini et al. [155].

Shutske, Sandner, and Jamieson identified people, animals, property, infrastructure, downtime, networks, and the environment as relevant exposure domains for autonomous agricultural machinery and found gaps in existing engineering coverage [156]. Building on that finding, this review recommends documenting controllability, approach safety, unexpected implement motion, remote-command status, third-party exposure, and the responsible authorizer. Mandatory responsibility and qualification rules remain those of the applicable standard, law, and organization; remote information does not itself verify the physical state.

This review recommends limited monitored operation only when the fault is minor, functional and safety boundaries remain satisfied, and explicit exit triggers are recorded. Where an applicable standard, regulation, or authorized safety procedure requires shutdown, guarding, isolation, or refusal of access—or where an unacceptable safety risk cannot be excluded—the machine must be brought to a safe state, and access must be prohibited until isolation is verified [140,141,142,143,144]. Temporary action restores control only and is not evidence of permanent repair.

Isolation covers mechanical motion, hydraulic pressure, electrical energy, and autonomous commands. Applicable machinery and agricultural control requirements make physical risk reduction and verified energy control prerequisites for hazardous access [140,141,142,143,144]. Guards, power, stored energy, and remote commands are handled as the applicable procedure requires; this review additionally recommends recording the asset, jurisdiction, verifier, time, state, and release authority. Closing an interface is not physical isolation, and model confidence cannot replace verification.

Applicable machinery safety and agricultural control requirements make physical risk reduction and verified energy control prior conditions for hazardous access [140,141,142,143,144]. Required guards and shields must remain effective; relevant power and stored mechanical, hydraulic, and electrical energy must be controlled; and autonomous or remote commands must be inhibited where the applicable procedure requires it. This review additionally recommends recording the jurisdiction, equipment category, checked safeguard, verifier, time, and release criterion. Diagnostic confidence cannot replace physical verification.

Response may involve verified automatic recovery, dependable, degraded operation, or manual inspection. Aldrini, Chihi, and Sidhom synthesized detection, diagnosis, self-healing, and fault tolerance across 256 smart-manufacturing studies [155]. Atamuradov et al. emphasized uncertainty and field conditions in PHM implementation [157]. This review consequently checks credible evidence, effective isolation, and clear responsibility before work; alarm reset alone is not evidence of recovery. Atamuradov et al. organized PHM implementation around critical component analysis, condition monitoring selection, prognostic feature evaluation, and method and tool assessment, emphasizing uncertainty and field conditions [157]. On that basis, this review proposes three maintenance-entry checks: credible evidence, effective isolation, and clear responsibility. Insufficient evidence prompts review; ineffective isolation prohibits hazardous access under the applicable safety procedure; and unclear authority prevents work authorization. The resulting record should retain evidence, consequence, isolation status, authorizer, scope, and reversibility.

### 6.2. Disassembly, Repair, Remanufacturing, and Replacement Strategies

Repair selection begins after confirmation and isolation by redefining the target as damage location, material and geometry, load-bearing role, and functional consequence [158,159]. External status, operating evidence, part orientation, contamination control, nondestructive testing, dimensions, tolerances, and internal-damage uncertainty should be preserved before deciding repair, remanufacture, replacement, or scrapping. Cost enters only after safety, quality, and regulatory boundaries.

The disassembly and inspection start from the external status record, retaining the diagnostic evidence, operating conditions, and positional relationship before disassembly when the fault occurred, and then cleaning and removing oil stains and attached particles to avoid contaminants being misjudged as surface damage. During the disassembly process, the direction, matching relationship, and wear location of the parts should be marked to prevent the loss of cause-and-effect information during reassembly. After the initial inspection, carry out non-destructive testing and dimensional review to confirm cracks, pitting, spalling, deformation, wear depth, roundness, coaxiality, gaps, and sealing surface conditions, and match the test results with the original design tolerances, load-bearing levels, and failure modes in Section 3. Visibility on the surface does not mean that there is no internal damage. When the detection capability is insufficient, the disposal level should be increased, and “no defect found” cannot be used instead of “defect does not exist”.

Material removal, welding, laser cladding, additive deposition, replacement, and scrapping have different evidence needs [160]. Lyubenov et al. used off-zone temperature and heating rate to control arc surfacing heat and reported that remanufacturing may cost about half a new part, conditional on safety and quality [161]. Liu et al. emphasized repeatability and visual, thermal, acoustic, and spectral monitoring in laser cladding [162]. Records should retain material batch, energy input, speed, temperature, defects, and rework rather than final dimensions alone.

Wahab and Azman identified geometry, tolerance, material compatibility, and preprocessing as constraints for additive repair [163]. Agricultural shafts and structural interfaces require bond, residual stress, machining, and fatigue assessment. Bearings, seals, safety controllers, power modules, and high-voltage connectors generally favor traceable replacement when field repair cannot restore accuracy or insulation [46,164].

Wahab and Azman grouped metal additive repair into directed energy deposition, powder bed fusion, and cold spraying and identified geometry, tolerance, material compatibility, and preprocessing as principal constraints [163]. For agricultural shafts, gear seats, and structural connectors, this review recommends assessing bond strength, residual stress, machining allowance, and fatigue weakness before selecting repair. Replacement or scrapping is required only where the applicable design requirement, manufacturer instruction, or safety assessment does not permit an adequate margin to be demonstrated.

Regulation (EU) 2023/1230 links substantial modification to compliance responsibility [143]. Beneduce et al. discussed remanufacturing boundaries for structure, function, and safety and noted application from 20 January 2027 [165]. Accordingly, applicable load-bearing, safeguard, control, and approval requirements must be reassessed; the source’s undefined residual performance value is not adopted as a universal criterion. The process route remains traceable from pretreatment through deposition, machining, assembly, and independent acceptance.

The official text of Regulation (EU) 2023/1230 places machinery safety within essential health and safety requirements and makes a substantial modification relevant to the allocation of manufacturer responsibilities [143]. Beneduce et al. noted that the Regulation will apply from 20 January 2027 and examined acceptable boundaries for remanufactured products in terms of structural performance, functional requirements, and safety requirements [165]. After remanufacturing, it is therefore insufficient to show that a machine can operate. Load-bearing capacity, safeguards, control functions, and operational functions must be reconfirmed, and the repair must be assessed to determine whether it constitutes a substantial modification affecting compliance responsibilities. A residual performance value reported in the source was not adopted here as a general repair criterion because its variables and conditions of applicability were not defined.

The process route comprises pretreatment, repair deposition, any required heat treatment or stress relief, machining, cleaning, assembly, and review. Each step should be linked to personnel, equipment, material, parameters, and defect records. Section 6.2 determines repairability, process selection, and recoverable capability; Section 6.3 organizes people and parts; and Section 6.4 independently verifies effectiveness.

### 6.3. Maintenance Resources, Spare Parts Scheduling, and Field Execution

Executable work orders should link the asset, evidence, isolation, solution, time window, authorizer, people, parts, tools, qualifications, and stop criteria [166,167,168]. Hu, Boylan, Chen, and Labib reported intermittent demand, shortage-driven downtime, and annual parts consumption up to 2.5% of purchase price in mechanical equipment [169]. This supports risk- and season-aware stocking while recording substitutions and deviations; it is not a universal agricultural cost rule.

Spare parts policies should reflect safety criticality, demand uncertainty, and lead time. Safety-critical bearings, seals, controllers, and hydraulic components with severe downtime consequences require minimum stock or regional placement. High-value, long-lead items with intermittent demand require targeted reserves based on failure history, fleet size, and predicted risk; low-value common parts may be centralized. Hu, Boylan, Chen, and Labib noted that spare parts demand is intermittent, inventories are large, shortages increase downtime, and annual parts consumption for mechanical equipment may reach 2.5% of purchase price [169]. This evidence supports considering seasonal failure concentration and supply delay, not average demand alone. Work orders should record shortages, substitutes, temporary measures, and expected completion; substitutions require approval when material, interface, or safety level changes.

Yao et al. modeled agricultural service locations under coverage, distance, and uncertain demand [170], while Li, Ren, and Wang proposed a three-level parts center, service station, and service unit network [171]. The synthesis supports positioning mobile capability near seasonal work and routing complex repair to specialist sites. Mandatory qualifications follow applicable rules; this review recommends version-controlled documents and records of identity, orientation, defects, fastening, clearances, seals, and connections [172].

Work-order execution covers preparation, field confirmation, isolation review, disassembly, repair or replacement, reassembly, commissioning, and handover [172]. Applicable standards, regulations, and organizational authorization determine mandatory qualifications for high-voltage, hydraulic, welding, lifting, and autonomous control hazards. This review additionally recommends version-controlled drawings and process sheets and records of identity, orientation, mating relationships, defects, fastening, clearances, seals, and connections.

**Figure 14:**
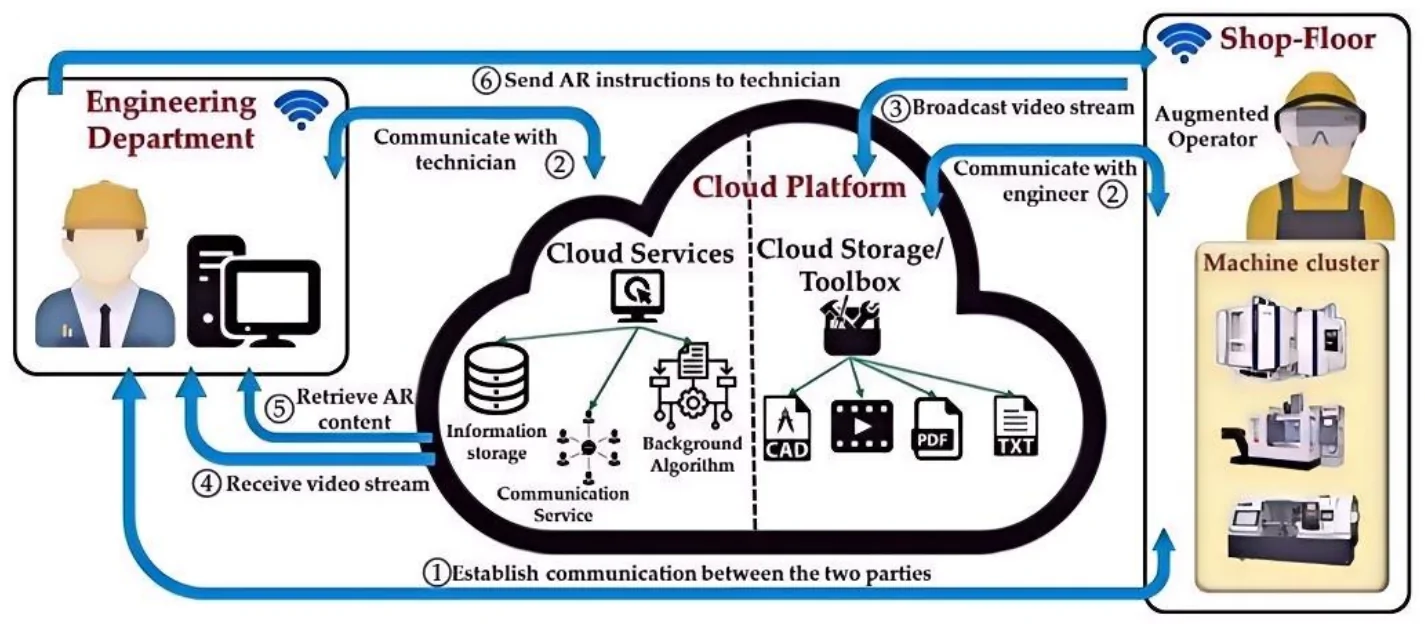
Remote maintenance collaboration architecture based on cloud platform and augmented reality. Source: Reproduced without modification from Mourtzis et al. [173] under CC BY 4.0.

Mourtzis, Siatras, and Angelopoulos demonstrated laboratory and industrial feasibility of augmented-reality remote support [173]. It can share views, marks, procedures, and findings but cannot replace on-site isolation. Their 60–70% maintenance cost and up-to 70% replacement cost figures are industrial context, not agricultural parameters. Execution closes only with a traceable record of personnel, parts, deviations, stops, and unfinished work; new damage or failed isolation returns the case for review.The architecture is shown in Figure 14.

Field execution ends with a complete process record, not merely a closed work order. The record should identify personnel, time, machine, parts, substitutions, tools, abnormalities, stoppages, and unfinished work, while retaining evidence of shortages or process deviations. Work should stop and return to Section 6.2 if damage exceeds the order scope, isolation fails, parts do not match, or reassembly reveals new abnormalities. Independent performance and safety acceptance begins only after the execution record is traceable.

### 6.4. Maintenance Quality Acceptance, Effectiveness Feedback, and Knowledge Updating

Reset, work-order closure, or start-up does not establish recovery [174]. Applicable standards and regulations require restoration of safeguards and safety-related functions before service [140,141,142,143,144]. This review then recommends staged static, no-load, representative load, field, and short-term checks against the pre-repair baseline. The regulatory gate is mandatory where applicable; agricultural function, quality, and persistence are additional review-level acceptance criteria.

Acceptance should progress through static inspection, no-load testing, rated or representative load testing, field verification, and short-term follow-up. Static inspection covers assembly, fastening, clearances, seals, fluids, electrical connections, controller versions, and safety devices; misassembly, leakage, looseness, or incomplete records preclude dynamic testing. No-load testing checks starting, steering, braking, shifting, hydraulics, communication, vibration, temperature, noise, and pressure. Passing it confirms only basic function, not restored load-bearing capacity.

Before representative load or field testing, the applicable regulatory safety check must confirm restored guards, warnings, interlocks, emergency functions, and safety-related control states and assess whether repair or software change alters the approval or compliance basis [140,141,142,143,144]. This mandatory check is necessary but not sufficient for the review’s broader acceptance proposal, which also examines agricultural function, operational quality, and persistence under the intended mission profile.

Representative load and field tests should compare output, pressure flow, temperature, energy, control response, traction, depth, uniformity, trajectory, and sustained operation [175,176,177,178,179,180,181]. Reduced vibration or downtime cannot substitute for task quality or safety. For laser-clad or additive parts, Chen et al. identified optical, acoustic, laser line, and in situ X-ray evidence for process consistency and defect correction [182]; missing process evidence lowers acceptance strength even when dimensions pass.

Acceptance testing of laser-clad or additively repaired parts should incorporate evidence of process quality. Chen et al. identified consistency and repeatability as continuing challenges in laser additive manufacturing; optical, acoustic, laser line scanning, and in situ X-ray methods can detect melt-pool and deposition defects, while multisensor fusion and closed-loop control can support defect correction [182]. Acceptance should therefore assess more than final dimensions. It should also trace temperature, geometry, acoustic anomalies, parameter adjustments, and defect rework throughout cladding or deposition. When process evidence is unavailable, the strength of the acceptance evidence should be downgraded even if the final surface dimensions satisfy specification.

Short-term follow-up tests recurrence and persistence under comparable load, environment, implementation, and operator conditions. Zio identified practical gaps among monitoring, diagnosis, prognosis, and maintenance [183]; this review recommends one asset identity and time base across pre-repair evidence, execution, and post-repair verification [184]. Traceability connects the interface but does not prove an end-to-end validated chain.

Zio reported persistent gaps between condition monitoring, diagnosis, prognostics, and maintenance decisions in practical PHM [183]. To address record fragmentation, this review recommends carrying the same asset identity, variable definitions, and time base from pre-repair evidence through repair and post-repair verification [184]. This produces traceability at the interface; it is not evidence that a complete chain has already been validated.

Acceptance outcomes are labeled true repair, temporary relief, misdiagnosis or misrepair, restoration failure, or repair-induced fault, with negative and uncertain outcomes retained. Li et al.’s closed-loop maintenance framework supplies outcome feedback and pre–post comparison [185]. The proposed repair-effectiveness label adds agricultural quality, safety margin, task conditions, recovery duration, evidence strength, and confounders; it remains an unvalidated organizational construct.

Li et al. proposed a closed-loop framework that updates maintenance strategies with changing machine conditions and uncertainty and revises reliability, availability, and maintainability data [185]. Building on that foundation, this review proposes a repair-effectiveness label. It directly inherits outcome feedback and pre-/post-maintenance comparison, adapts them to agricultural operational quality, safety margins, task conditions, and the duration of recovery, and resynthesizes five outcome categories—true repair, temporary relief, misdiagnosis or misrepair, restoration failure, and repair-induced fault—with the repair target, recovery magnitude, evidence level, acceptance conditions, and confounders. The label taxonomy and minimum fields are original to this review as an organizational proposal and remain to be validated prospectively. Only accepted facts should be written back; contradictory findings should correct the causal record while preserving the earlier diagnosis and its error.

## 7. Cases of Agricultural Machinery Fault Diagnosis, Prognostics, and Maintenance

This chapter groups cases into four links: defining fault entities; detecting condition and confirming diagnoses; forecasting degradation and making maintenance decisions; and executing maintenance and accepting recovery. The sources involve different machines, studies, and repair processes. They are therefore treated as illustrative composite mappings, not as validation of a complete closed loop on the same machine. Table 8 separates directly observed results from this review’s reconstruction, records the reported follow-up, and identifies the unsupported links before the detailed cases are discussed.

### 7.1. Cases for Defining Fault Formation, Failure Boundaries, and Repair Targets

#### 7.1.1. Defining the Failure Object in Tractor PTO Shaft Fracture

Mandal et al. reconstructed a natural PTO shaft fracture on a Krisishakti 125DI tractor. A violent noise preceded cultivator stoppage; at 300 N·m, equivalent stress was 632.08 MPa and deformation was 0.1855 mm. Fractography located initiation at a section transition, and the authors estimated the failure load as about 40% torsion and 60% bending [186]. The supported chain is sudden tillage resistance, stress concentration, torsional cracking, loss of axial restraint, bending, and power interruption; inspection should target the fracture, transition, splines, and interfaces.

#### 7.1.2. Defining the Failure Object in Tractor Front-Axle Fatigue Fracture

**Figure 15:**
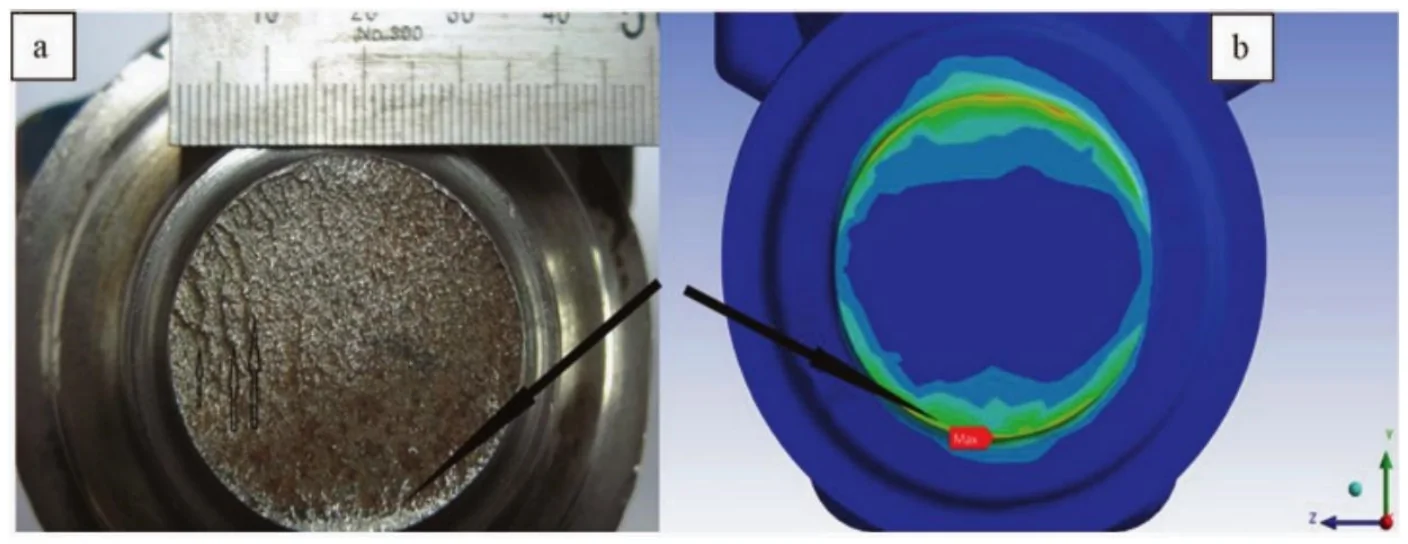
Comparison between (**a**) the actual damage area of the tractor front axle and (**b**) the finite element stress concentration area. The color scale in (**b**) indicates stress magnitude from low (blue) to high (red). Source: Reproduced without modification from Yavuz [187] under CC BY 4.0.

Yavuz reconstructed an in-service front-axle fracture in 50CrMo4 steel. Surface and core hardness were 900 and 323 HV, the single-wheel static load was 5.9 kN, and the critical static stress was about 115 MPa [187]. Mechanical damage at the shaft end and a section transition supported a chain of surface embrittlement, impact and cyclic loading, fatigue growth, and sudden fracture [188,189]. Figure 15 links physical damage with stress concentration; the evidence crosses load-bearing, steering, and safety boundaries.

#### 7.1.3. Defining Failure Objects in PTO-Driven Rotary-Rake Gears and Working Parts

Ghatge et al. followed five nine-arm rotary rakes for 1500 h. A total of 48 of 212 components failed (22.6%); gear failures clustered around 137–164 h, and residue blockage raised torque from about 90 to 200 N·m [190]. The supported chain runs from blocked rake teeth through arms to pinion and internal gears, producing tooth root overload, rotor stoppage, or reduced raking quality. Inspection should distinguish working part, gear pair, bearing, and interface damage.

#### 7.1.4. Repair Targets and Fault Consequences

All three cases concern natural failures in service or field operation. The supported fault hypotheses are impact-and-torsion fracture of a PTO shaft, embrittlement-associated fatigue fracture of a front axle, and obstacle-induced overload and gear tooth fracture in a rotary rake. Consequences include interrupted implement power, loss of load-bearing and steering safety, rotor stoppage, and reduced raking quality. Subsequent inspection should focus on the identified shaft transition, front-axle end and hub-support region, gear–pinion pair, and upstream working parts.

### 7.2. Multisource Condition Monitoring, Fault Localization, and Diagnostic Confirmation

#### 7.2.1. Data Contracts for a Combine Harvester and a Powershift Tractor

Qiu et al. instrumented a combine power chain with speed, travel, temperature, and header-height sensors and adopted dual-channel sensing after detecting zeros and anomalies [191]. Hao et al. paired triaxial bearing vibration with speed, current, and torque on a threshing drum rig [192]. Silva et al. linked clutch-pressure switches to tractor identity, operating hours, and gearbox specifications [193]. Together, the cases support a compact machine–component–task–condition–time data contract, but not identical validation settings.

On a multistage threshing drum test rig, Hao et al. magnetically mounted a triaxial accelerometer on the drum bearing housing, measured speed photoelectrically, and recorded loading current and torque so that vibration could be interpreted against speed and load [192]. Silva et al. used four pressure switches to monitor clutch pressure in a New Holland T8.270 18 × 4 full-powershift transmission and linked the data to chassis number, operating hours, and gearbox and engine specifications [193]. Together, these fields create a data contract for tracing the machine, component, task, operating condition, time, unit, and tag.

#### 7.2.2. Data Quality Control and Removal of Operating Condition Effects

Qiu et al. reported 5000 processed samples and 2600 normal samples; the eight fault categories sum to 2400, not the separately stated 2500. Faults were manual, travel speed was 6 ± 0.5 km/h, and validation was simulated or indoor [191]. Hao et al. found load affected vibration more than speed [192], while Silva et al.’s raised-wheel clutch test was controlled rather than tractive field operation [193]. None used an independent machine or time-separated validation, so the cases do not establish cross-machine generalization.

The threshing drum study separated four speed settings from four material load settings and found that load affected vibration more strongly than speed [192]. In the tractor clutch experiment, gears were shifted sequentially with one rear wheel raised and front-wheel drive disengaged; the resulting pressure signals were controlled simulations rather than evidence under real tractive load [193]. None of the three studies separated training and testing by independent machine or time period, so they do not demonstrate cross-machine diagnostic generalization.

#### 7.2.3. Fault Detection, Localization, Type Identification, and Severity Assessment

The combined system reported 97.46% overall recognition, including 100% for severe blockage and 90.15% for slight blockage; overlap with low-speed normal operation limits the average metric [191]. Hao et al. associated blockage with about 25 Hz and 0.55 mm displacement and slip with about 50 Hz and 0.184 mm, but both were manually produced rig states [192]. These results support anomaly and operating state discrimination, not natural-damage or field-consequence claims.

Hao et al. localized drum abnormalities to vertical vibration at the shaft end. Blockage produced a low-frequency peak near 25 Hz and maximum displacement near 0.55 mm, whereas slip concentrated near 50 Hz with maximum displacement near 0.184 mm [192]. Both faults, however, were produced by manual loading on a test rig. Without evidence from natural damage, disassembly, threshing loss, or cleaning quality, the study confirms abnormal operating states but not material damage or field consequences.

Normal clutch pressure for the T8.270 was 21–25 bar. C3 pressure fluctuated between 15.5 and 24.5 bar, indicating internal leakage, while the high-gear clutch reached only 7.679 bar, preventing engagement of gears 13–18. The fault was localized to the main-clutch-shaft Teflon sealing ring and associated electrohydraulic valves rather than the complete gearbox [193].

#### 7.2.4. Diagnostic Passport and Maintenance Triggers

The cases support different passport fields: combine blockage needs operating condition and abstention records; threshing rig signals identify blockage or slip but not natural damage; and abnormal clutch pressure plus failed gear engagement provides direct functional evidence. Table 9 therefore compares provenance, real fault authenticity, equipment independence, uncertainty, and deployment rather than ranking methods by accuracy alone. Severe persistent blockage, slip, or inadequate engagement pressure can trigger review, whereas brief load transitions or low confidence cannot alone justify repair.

Table 9 compares the evidential requirements of the principal diagnostic and prognostic method families reviewed in Section 4 and Section 5. It emphasizes dataset provenance, real fault authenticity, equipment independence, validation design, uncertainty, and deployment rather than ranking methods by a single accuracy value.

Severe blockage, persistent excessive slip, or clutch pressure too low to engage a gear are functional triggers for prognostic and maintenance decisions. A brief speed reduction, load transition, or low-confidence classification should not by itself initiate repair.

### 7.3. Degradation Prognostics, Risk Warning, and Maintenance Decision-Making Cases

#### 7.3.1. Tractor Engine Oil Condition and Degradation Stages

Hönig et al. sampled oil every 100 h to 600 h in a heavy-duty Case QuadTrac 600, lighter-duty Magnum RowTrac 340, and transport-duty New Holland T7.250, with three measurements per sample [194]. In the QuadTrac, 8 ppm tin at 100 h, silicon increasing from 18 to 30 ppm, a loose intake clamp, and sulfation approaching 0.2% by 300 h supported a reconstructed progression from early to accelerated degradation. The staging is this review’s mapping, and absolute thresholds should not transfer across the three duty profiles. Zhong et al.’s nonparametric health index and statistical threshold provide a separate methodological basis for condition-stage interpretation [195].

For this review, the sequence from new-oil baseline through persistent multivariable deterioration and reduced functional margin is mapped to normal, early-degradation, accelerated-degradation, and critical stages. This mapping is a reconstruction, not the periodization used by the source. Absolute thresholds from the heavily loaded QuadTrac should not be transferred to the transport-duty T7.250, because normal operating condition differences would be misclassified as degradation.

#### 7.3.2. Trend Prognostics and Remaining Operational Capacity

The study used ARIMA to extrapolate the sequence observed through 600 h to 900 h. With unchanged load and trend and continued monitoring every 100 h, Mogul oil was considered serviceable to 900 h; Shell oil retained a 600 h interval because of occasional heavy loads; and Akcela oil was assigned a 300 h interval [194]. These are oil change intervals, not remaining engine life. Because the study reports no confidence intervals, coverage, remaining workload, or natural failure endpoint, its evidence is limited to in-service sampling with statistical extrapolation; the recommendation should not be transferred when load conditions change.

#### 7.3.3. Preventive-Replacement Thresholds and Harvest-Season Resource Constraints

Borowski et al. modeled tractor-trailer availability, minimal repair, station repair, and age-based replacement with transition probabilities 0.9080 and 0.0920 and Weibull parameters; stronger preventive replacement moved the modeled optimum earlier [196]. The case compares maintenance timing but lacks machine-specific diagnosis, warning lead time, and error rates. Warnings should therefore escalate only with persistence and concordant contamination, wear, and oil function evidence; the case does not demonstrate field warning performance.

**Figure 16:**
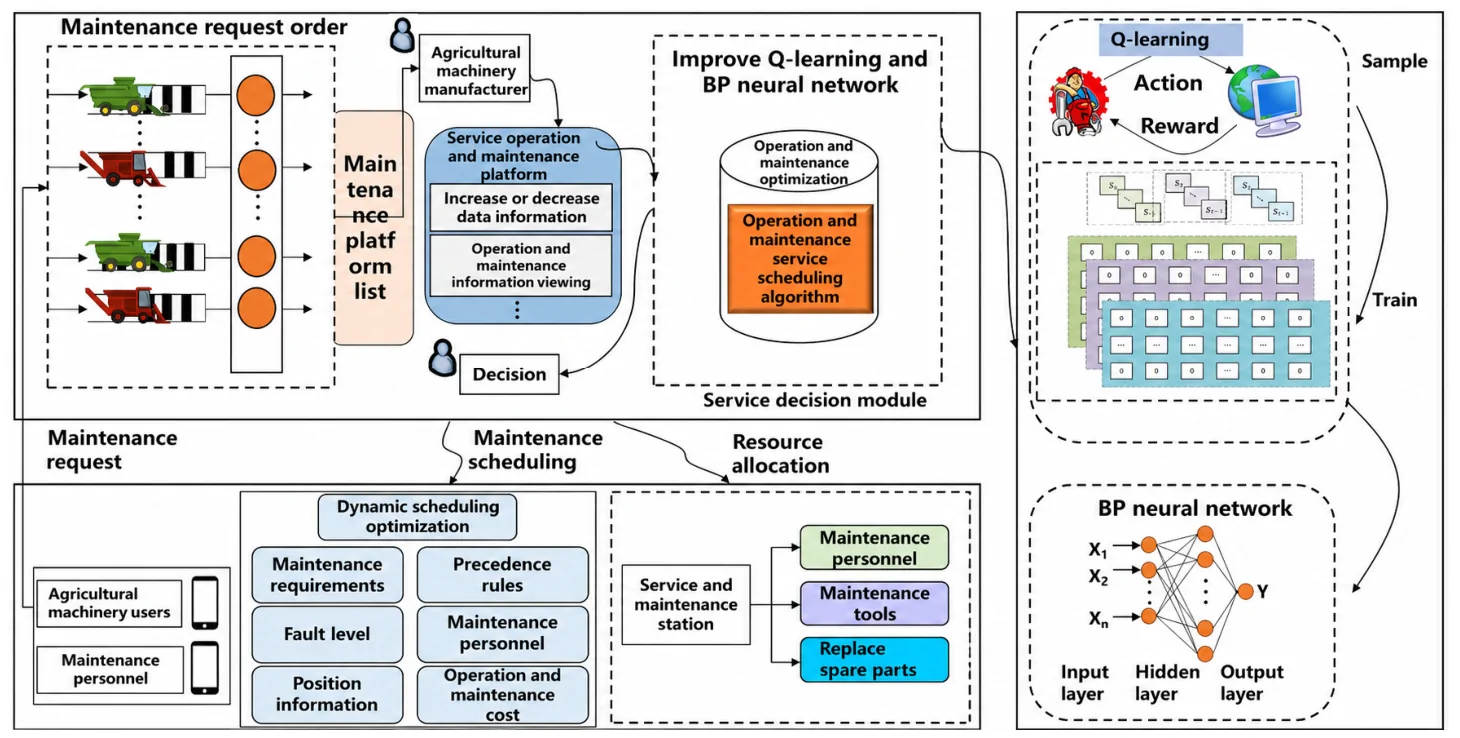
Overall framework of dynamic scheduling of maintenance tasks. Source: Reproduced without modification from Zhang et al. [197] under CC BY 4.0.

Zhang et al. scheduled combined service by fault level, time window, technician skills, tools, parts, distance, and cost, using weights 1, 2, and 5 for general, serious, and fatal faults [197]. Figure 16 supports resource feasibility after demand appears, not the validity of the preceding prognosis.

Warnings should progress from a single threshold crossing to hierarchical confirmation. The first crossing produces a candidate warning; persistence and concordant changes in contamination, wear, and oil function justify escalation. Conflicting indicators, missing data, or a changed task should trigger abstention and manual review. Because the cited cases do not report warning lead time, response delay, false positives, or false negatives, they do not demonstrate field early-warning performance.

#### 7.3.4. Maintenance Timing and Work-Order Decisions

A cautious case mapping plans QuadTrac oil service near 300 h only when multiple indicators agree and inspects the intake and friction pairs when contamination or wear metals rise [194]. The other tractors retain a 600 h baseline unless continuing evidence supports change. Missing data, load shifts, conflicting indices, or out-of-domain inputs trigger inspection rather than automatic action; work orders add weather, farming window, people, parts, downtime, and authority.

### 7.4. Field Maintenance, Recovery Acceptance, and Knowledge-Feedback Cases

#### 7.4.1. Two-Wheel-Tractor Fault Review and Safe-Boundary Entry

Nwakaire et al. found a seized carburetor, damaged ignition coil, corroded controls, seized wheels, worn tires, and a missing implement mount on a two-wheel tractor [198]. The study supports decomposition into fuel, ignition, running gear, and coupling faults but omitted shutdown, energy isolation, responsibility, verification, and release records. Under the proposed workflow, diagnosis is frozen, and fuel, ignition, and mechanical energy are verified before disassembly; without those records, maintenance entry is not traceable.

Under Section 6, the original diagnosis and decision record should be frozen, and fuel, ignition, and mechanical energy should be isolated and verified by the responsible person before disassembly. Without those records, maintenance safety is not traceable.

#### 7.4.2. Plowshare-Damage Review and Repair-Strategy Selection

Singh and Chatha observed abrasion, oxidation, and cracks in ordinary and hardfaced plowshares; the Terrahard-85 layer showed shrinkage cracks, brittle fracture, and edge damage [199]. Hardness alone therefore does not establish acceptance, because the reinforcement can become a new failure origin.

Kuznetsov et al. cleaned and inspected used plowshares and required at least 8 mm of base material around the bolt hole and 100 mm effective rear width before repair [200]. These source-specific criteria illustrate geometry- and connection-based repairability; they do not remove the need to inspect the actual damaged part.

#### 7.4.3. Repair Process, Field Execution, and Process Reinspection

In the two-wheel-tractor case, the ignition coil, carburetor, tires, implement mount, and flange were replaced or redesigned, but omitting fastening, fit, version, and abnormal-stop records limits traceability [198]. Singh and Chatha applied a 100 mm by 2 mm hardfacing layer and tested about 200 acres at 15 cm depth; HF-5M wore at 0.89 g/ha versus 1.53 g/ha for cracked Terrahard-85 [199].

Singh and Chatha applied a 100 mm long, 2 mm thick hardfacing layer to the plowshare cutting edge by shielded metal arc welding and tested it over about 200 acres of sandy loam at a 15 cm plowing depth. The chromium-carbide HF-5M layer wore at 0.89 g/ha, compared with 1.53 g/ha for the cracked Terrahard-85 layer [197].

Kuznetsov et al.’s cleaning, inspection, machining, brazing, and testing process produced about 119–123 MPa joint strength at 285–324 mm^2^ overlap [200]. Materials, parameters, cracking, and joint strength should therefore remain linked to stop or rework criteria.

#### 7.4.4. Return-to-Service Acceptance, Short-Term Follow-Up, and Knowledge Feedback

The refurbished two-wheel tractor restored field operation at 1.38 km/h, 0.032 ha/h, 61.50% efficiency, 0.43 L/h fuel use, 1.13 kN resistance, and 0.43 kW draft power, but lacked a comparable baseline and follow-up [198]. After 10 ha, Kuznetsov et al. reported 0.2 kg loss for reinforced versus 0.41–0.86 kg for ordinary plowshares and joint capacity 3.8–4.9 times design load [200]; Singh and Chatha support separate “HF-5M effective” and “Terrahard-85 crack damage” labels [199]. None demonstrated systematic knowledge write-back, so the cases support repair-effectiveness labeling, not a validated digital closed loop.

After 10 ha in loam, Kuznetsov et al. measured mass losses of approximately 0.41–0.86 kg for ordinary plowshares and 0.2 kg for cermet-reinforced plowshares; the repaired joints also withstood 3.8–4.9 times the design load. These bench and field results support an effective-repair label [200]. Singh and Chatha’s results require two simultaneous labels [199]: “HF-5M effective” and “Terrahard-85 crack damage.”

None of the three studies demonstrated knowledge base updating or systematic write-back of post-repair data. Records of actual damage, process parameters, recovery magnitude, verification conditions, and unsuccessful outcomes should therefore be added to diagnostic and maintenance knowledge bases as a recommendation, not as an empirically validated digital closed loop.

## 8. Conclusions and Outlook

### 8.1. Main Conclusions

First, the reviewed evidence shows that fault interpretation depends on the mission profile, implement, environment, and performance, operational quality, and safety boundaries. This review therefore recommends defining the fault entity and applicable boundary before comparing signals or algorithms.

Second, studies consistently separate initiating conditions, damage mechanisms, physical damage, component faults, symptoms, and system consequences. The proposed ontology preserves causal, propagation, control, interface, and consequence relations instead of treating correlation as causation.

Third, the literature supports multisource diagnosis and mechanism-informed checks but does not support accuracy alone as proof of trustworthiness. This review recommends a diagnostic passport containing identity, conditions, data quality, probability or interval, applicability, latency, conflicts, and abstention reasons; the passport is an organizational proposal requiring field validation.

Fourth, prognostic evidence should specify prediction origin, failure definition, future load assumption, horizon, and uncertainty. Controlled faults and random signal slices establish feasibility, whereas claims across machines, fields, and seasons require independent trajectories. This review recommends linking warnings to persistence, concordance, remaining work, consequence, and authorization.

Finally, the proposed closed-loop repair workflow links evidence review, safe isolation, repair-or-replacement decisions, resources, execution, tiered acceptance, and outcome feedback. No included study followed the same machine or fleet through all of these stages. Evidence supports individual links; their connection, together with the risk trajectory and repair-effectiveness label, is a theoretical synthesis rather than an end-to-end validation claim.

Overall, evidence is strongest for controlled-fault identification, selected field monitoring, and localized compensation and remains limited for cross-condition prognostics, safety response, post-repair verification, and sustained fleet deployment. Mandatory statements in this review apply only where supported by the cited standard, law, regulation, or authorized safety procedure; other workflow fields are recommendations or conceptual requirements.

### 8.2. Areas of Consensus and Continuing Disagreement

Across the literature, multisource evidence, operating context, transfer limits, and uncertainty are recurring deployment concerns. The review-derived recommendation is that diagnostic and prognostic outputs report probability, explanation, horizon, uncertainty, resource use, and abstention, while maintenance decisions also record farming windows, personnel, parts, safety, and operational quality. These fields are proposed for traceability and are not themselves regulatory mandates.

Continuing disagreements concern whether artificially introduced faults represent natural degradation, how health and failure thresholds should be harmonized, the domains in which knowledge constraints remain valid, whether unknown samples should trigger abstention or continued classification, and where responsibility lies among automatic derating, self-healing, and manual takeover. Differences in the machines and validation conditions prevent these questions from being resolved through a single accuracy metric.

### 8.3. Limitations of This Review

This article is a review rather than a systematic review. Literature identification, selection, and interpretation therefore involved author judgment. Although database searches and citation tracing were used to improve coverage, some relevant studies may have been omitted. No registered review protocol, formal risk-of-bias assessment, or quantitative meta-analysis was undertaken.

The main limitation is the scarcity of direct agricultural evidence spanning the complete operations-and-maintenance chain. Differences in machine structure, operating conditions, sensor configuration, and safety boundaries preclude aggregation of many diagnostic and prognostic metrics. Some evidence on knowledge graphs, remaining useful life prediction, digital twins, edge inference, and remanufacturing comes from other industries and can support methods but not agricultural performance claims. Because the selected studies differ substantially in fault definitions, units of analysis, data partitions, and validation scenarios, this review uses qualitative synthesis rather than a pooled effect size. The diagnostic passport, risk trajectory, and repair-effectiveness label are theoretical organizational tools and have not been jointly validated on a single machine.

### 8.4. Research Priorities and Overall Assessment

Future research should first build longitudinal datasets organized by machine. These datasets should retain continuous signals, operational events, environmental loads, maintenance versions, real-world faults, failed predictions, abstentions, and repeat repairs, with validation partitions separated by machine, time, field, and season. Diagnostic accuracy, probability calibration, abstention, warning lead time, interval coverage, operational quality recovery, safety margin, and the persistence of repair outcomes should then be evaluated separately. Field trials should report the number of independent machines and events, rather than treating signal windows as independent fault samples.

Methodologically, research should develop mechanism–data fusion models with out-of-domain detection and safe abstention, while specifying the conditions for transfer across machine types, edge resource budgets, offline fallback, and model rollback. At the decision level, future risk should be integrated with farming windows, remaining workload, personnel, and spare parts availability; automated actions should be subject to explicit authorization and safety boundaries. At the repair level, the field workflow should connect isolation, disassembly findings, process parameters, acceptance results, and short-term follow-up, and should feed negative outcomes and confounding factors back into the knowledge base.

Overall, fault diagnosis for agricultural machinery has accumulated substantial evidence from controlled experiments and selected field-monitoring studies, whereas prognostics, repair, and fleet-scale implementation remain at an earlier stage of evidence development. The next stage should not focus solely on improving the accuracy of a single model. Each conclusion should state its applicable conditions, evidence strength, uncertainty, action authority, and verification outcome, thereby turning diagnosis, prognostics, prevention, repair, and recovery into a traceable engineering chain.

## Figures and Tables

**Table 1:** Comparison of the proposed constructs with existing frameworks and their validation boundaries.

Proposed Construct	Closest Existing Foundations	Directly Inherited	Agricultural Adaptation/Resynthesis	Original Element and Present Validation Boundary
Diagnostic passport	PHM; condition-based maintenance; digital thread	Asset state, provenance, uncertainty, and applicability	Binds machine, implement, task, operating condition, abstention, and auditable evidence	Minimum field-and-abstention interface proposed here; not an industry standard; complete schema unvalidated
Risk evolution trajectory	Prognostics; predictive maintenance; dynamic risk	Health/RUL updating, thresholds, and consequence	Adds remaining field work, farming window, weather, implement, and authorization gates	Time-indexed health–exposure–consequence–action record proposed here; no prospective validation
Repair-effectiveness label	Maintenance feedback; repair acceptance; digital twin	Pre–post comparison and outcome feedback	Adds operational quality, safety margin, task condition, persistence, and confounders	Five-category label and minimum fields proposed here; prospective reliability unknown
Case-evidence matrix	Evidence mapping and review appraisal	Study design, observation, and follow-up fields	Maps evidence to agricultural closed-loop stages and separates observation from reconstruction	Review-level audit tool proposed here; does not validate a machine or intervention
Closed-loop repair workflow	PHM/PdM/CBM; digital twin/thread; self-healing; maintenance decisions	Monitoring, prediction, intervention, resources, and feedback	Adds safe isolation, human authority, farming constraints, and tiered field acceptance	Integrated interface and responsibility model proposed here; not yet shown end-to-end on one machine

**Table 2:** Cross-industry evidence admission and transfer-boundary criteria.

Admission Dimension	Question	Minimum Acceptable Basis	Mismatch Treatment	Permitted Inference
Physical mechanism	Is degradation governed by comparable physics?	Same dominant damage or control mechanism	Reject if the mechanism is different	Mechanism representation or constraint
Load characteristics	Are amplitude, cycle, impact, and transient patterns comparable?	Overlapping normalized load regime or justified scaling	Record range and prohibit numerical extrapolation	Feature, stressor, or test design transfer
Equipment structure/interface	Are energy, material, information, or control paths comparable?	Equivalent functional path and sensor/actuator interface	Restrict to the shared subsystem	Architecture or interface transfer
Operating environment	Are dust, moisture, temperature, vibration, and connectivity comparable?	Relevant exposure overlaps or is explicitly stress-tested	Require agricultural revalidation	Robustness requirement only
Fault mode-signal relation	Does the fault produce comparable observables?	Same causal direction between fault and signal	Treat as hypothesis if confounded	Signal representation or diagnostic logic
Validation conditions	Are fault authenticity, independence, and temporal design comparable?	Design supports the intended claim	Downgrade to feasibility and state the gap	Validation principle; never direct performance transfer

**Table 3:** Distribution of direct agricultural and cross-industry evidence by primary framework link (one primary assignment per publication).

Primary Framework Link	Direct Agricultural Evidence	Cross-Industry Methodological Evidence	Total
Fault mechanisms and system boundaries	17	7	24
Current state diagnosis and trustworthy delivery	48	40	88
Prognostics	4	20	24
Risk control	2	12	14
Post-repair verification	5	5	10
Maintenance and repair	8	12	20
Illustrative prognostics/repair cases	7	1	8
Total	97	98	195

**Table 4:** Agricultural machinery fault ontology, propagation relationship and failure boundary mapping.

Ontology Level	Typical Objects	Fault/Status Mode	Formation Conditions and Communication Relationship	Key Observable Evidence	Failure Boundaries and Task Consequences	Boundary Decision Rules
System and task layer	Complete machine, attached implement, work objects, task profile	Normal; degradation; malfunction; failure	Change the basis, load direction, and allowable range for tasks such as transportation, light load, heavy load plowing, traction impact, etc.	Speed, stability, braking, traction, energy consumption, operational quality, and their health benchmarks	The boundaries of power, safety, or operational quality have been breached; merely continuing to operate does not prove that there is no fault	Degradation: deviation from the baseline but still meeting the function; failure: abnormal component/interface weakening the function or increasing the risk of failure; failure: the specified function or boundary has been lost
Power–transmission–walking layer	Engine, fuel and intake and exhaust, gears/shafts/bearings, clutches, tires/tracks	Abnormal combustion/fuel supply; wear, pitting, broken teeth, increased gaps; slipping, deviation, and tensioning imbalance	Overload, shock, insufficient lubrication, contamination, assembly eccentricity, and thermal effects; propagated by torque and mechanical shock	Speed, torque, temperature, vibration, meshing impact, traction force, and upstream and downstream loads	Power reduction or interruption; unstable shifting/traction; structural load bearing, braking, or driving stability out of bounds	Decrease in speed, vibration, and temperature rise are consequential symptoms, and common causes should be examined by combining upstream power and downstream load
Hydraulic layer	Pumps, valves, cylinders, motors, filters, and pipelines	Stagnation, impact, cavitation, stuck, internal/external leakage, and composite failures	Oil contamination, seal degradation, pump valve wear, insufficient oil suction, temperature changes; propagated through the pressure-flow chain	Pump and valve inlet and outlet pressure/flow, oil temperature, pollution degree, execution displacement, and response delay	Insufficient effective output force or execution ability; pressure/flow out of bounds; slow movement leading to unqualified working depth or application amount	Pressure fluctuations or noise cannot be determined alone; at least inlet–outlet and command-response comparisons are required
Electrical and Perceptual Layer	Energy storage, power converters, motors, connectors; pressure/temperature/position/inertial/vision sensors	Power supply interruption, insulation degradation, thermal failure; fixed value, offset, drift, mutation, noise, loss of connection, missing data	Aging, abnormal power supply, loose installation, environmental interference; control judgment affected by power supply, magnetic field, or information chain	Bus voltage, current, temperature, insulation; multisensor consistency, timestamps, data freshness, and physical residuals	Abnormal torque or energy utilization; inconsistent data–physical state causing misjudgment of load, attitude, or object	Perceived anomalies should be distinguished from real operating condition changes; this review recommends recording desynchronization, stale values, and missing measurements as separate states
Control and communication layer	Embedded controller, task logic, parameter table, CAN/remote link	Reset, I/O failure, mode/parameter error, limiting failure; loss of connection, delay, out of sync	Hardware protection, software logic errors, complex terrain/weather and link abnormalities; propagated through instructions and status information	Controller event log, mode/parameter version, communication status, command–feedback difference, control cycle, and delay	Oscillation, overshoot, action lag, trajectory deviation; loss of emergency control or manual takeover capabilities	Current evidence supports loss of connection, latency, and desynchronization; this should not be used to extend inferences about bus congestion or network attack mechanisms
Execution and task execution layer	Motor, proportional valve, clutch, steering mechanism, seeding/fertilizing/spraying device	Coil burned out, drive overheated, valve core stuck, clogged, connecting rod loose, feedback failure	The control deviation is converted into displacement, rotational speed or flow deviation through the execution interface, and then transmitted to the operational output	Control instructions, execution feedback, machine position, trajectory, sowing depth, plant spacing, application amount, and coverage rate	Instructions cannot be implemented; operational quality is too poor; trajectory/steering is out of control, causing safety risks	The fault ontology simultaneously records fault levels, action interfaces, time consistency, physical responses, and task consequences.
Interfaces and multiple fault layers	Power source, transmission, and hydraulic load; perception—communication, decision-making, execution, and feedback interface	Common cause failures; statistical correlation; functional dependencies; true cascading; amplification or redundant suppression	Dust, muddy water, overload, low temperature, or power supply fluctuations can form a common cause; a claimed cascade should identify the initiating event, medium, trigger condition, and temporal sequence	Upstream and downstream timing, load/state changes, propagation paths, counterevidence; safety, quality, shutdown, and repair accessibility	Multiple node functions are lost or consequences are amplified; key nodes are comprehensively identified by propagation possibility, consequence severity, and detection difficulty	At the same time, anomalies are not equal to cascades; statistical correlation does not prove physical transmission; when using observability, take its inverse

**Table 5:** Agricultural machinery diagnostic tasks and multisource data contract mapping.

Diagnostic Tasks	Output and Label Granularity	Core Variables and Measuring Point Configuration	Data Sources and Synchronization Requirements	Identity and Operating Condition Fields	Quality and Traceability Fields	Minimum Evidence and Use Boundaries
Anomaly detection	Whether it deviates from the normal operating domain; abnormal start and end time, direction, and amplitude	Cover health benchmarks and load variables; prioritize the placement of highly sensitive measurement points and retain environment/load references	Mainly onboard continuous data; unified clock; normal low-frequency monitoring, abnormal triggering high-frequency sampling, and saving the cache before and after the event	Equipment/machine model, implement, field, task, operating condition, operator, maintenance version	Number of valid samples, missing data marks, synchronization deviation, signal-to-noise ratio, drift, outlier ratio, trigger status	A single temperature rise or vibration only supports abnormal detection; when it exceeds the reference domain or the data quality is insufficient, the confidence level is reduced or the judgment is withheld
Fault location	The subsystem, component, or interface where the exception is located; a collection of candidate locations	Arrange upstream and downstream comparisons along the energy/material/information chain: power source–gearbox–output; pump valve inlet–outlet; command–feedback	Alignment of physical quantities, controller logs, and discrete events; records channels, controller sources, communication status, and link delays	Components, interfaces, installation locations, channel numbers, controller sources, and current mounting relationships	Timestamp source, synchronization error, range, units, calibration status, link, and storage location	Each positioning hypothesis has at least positive evidence, upstream and downstream comparisons and possible counterevidence; when key measurement points are missing, a candidate set is output
Type recognition	Use fault-sensitive variables and operating condition variables in combination; mechanical, electrical, hydraulic, and operating signals should be interpreted through their interfaces	Use fault-sensitive variables and operating condition variables in combination; mechanical, electrical, hydraulic, and operating signals must not be interpreted apart from the interface relationship	Airborne data, controller logs, remote sensing/UAV background, environmental loads, and maintenance records are associated with a unified identity and time	Faulty body version, machine model/component, task, load, soil/crop/slope, software, and maintenance version	Label basis, confirmation method, fault formation method, data source, processing version, and original record link	Accompanying symptoms do not establish the root cause; this review recommends verifying the applicability domain across machine types or unknown operating conditions, and multiple candidates may be retained or withheld when the evidence conflicts
Severity estimate	Degree and range of functional loss; impact on safety, quality, downtime, and maintenance	Joint measurement of state variables and task output: torque/pressure/flow/temperature, execution ability, seeding depth/application amount/trajectory, etc.	Improve key channel sampling bandwidth and synchronization accuracy; continuous trends and event windows are retained at the same time	Rated boundaries, mission objectives, load levels, operating objects, operating phases, and repair accessibility conditions	Baseline versions, measurement uncertainty, probabilities/intervals, boundary sources, completeness, and drift records	Proposed assessment: estimate relative to the mission profile and allowable boundaries; an uncalibrated single composite score may not be used as a substitute for the four-dimensional consequence assessment
Universal contract for all tasks	Diagnosis object, output type, time range, confidence/abstention status	Fault-sensitive variables, sensing source, installation location, sampling, and synchronization; data field item-by-item mapping	Airborne, remote sensing/UAV, controller logs, environmental loads, maintenance records; unified identity and clock	Equipment number, model, components, machines, fields, tasks, operating conditions, operators, maintenance versions	Collection time, frequency, unit, range, source, link, quality mark, label basis, and traceability chain	Record trigger variables, thresholds, front and rear windows and trigger failure fallback; this review recommends that manual records include location, confirmation method, replacement parts, and time

**Table 6:** Agricultural machinery fault detection tasks, system links, methods, and fault representation mapping.

Detection Tasks	System Links and Main Data	Testing or Diagnostic Methods	Data Processing Methods	Fault Characterization and Output
Anomaly detection	Sensor collection, controller logs, and operating condition monitoring; vibration, temperature, pressure, flow, speed, current, and environmental load	Threshold judgment, health benchmark comparison, model residuals, single-class learning, and anomaly scoring	Integrity check, denoising, time alignment, missing markers, operating condition normalization, and sliding window segmentation	Deviation degree from normal operating domain, abnormal direction, amplitude, start and end time, abnormal probability, and abstention status
Fault location	Upstream and downstream measuring points arranged along energy, material, information, and control chains; command, response, and event logs	Causal path reasoning, model residual analysis, expert rules, knowledge graph, and multisource evidence fusion	Multi-channel synchronization, upstream and downstream comparison, residual calculation, event correlation, and conflict evidence screening	Faulty subsystem, component or interface location, set of candidate locations, support paths, and disproof conditions
Type recognition	Airborne signals, spectrum, and frequency diagram; equipment identity, mission conditions, maintenance records, and fault labels	Statistical classification, machine learning, deep learning, case reasoning, and mechanism–data fusion	Filtering, resampling, order tracking, time-frequency transformation, feature selection, data enhancement, and domain adaptation	Fault category, candidate root cause, identification probability, applicable operating conditions, mechanism consistency, and unknown category abstention
Severity estimate	State variables and task output; torque, pressure, temperature, execution capacity, operational quality, and safety indicators	Regression analysis, probability model, degradation classification, boundary judgment and multi-index fusion	Benchmark correction, trend extraction, interval estimation, task boundary comparison, and uncertainty quantification	Failure level, loss of function, probability or confidence interval, and safety, quality, downtime, and repair impacts
Trusted diagnostic delivery	Comprehensive evidence of acquisition, communication, computing, diagnosis, and human–computer interaction	Probability calibration, uncertainty assessment, decision fusion, out-of-area detection and security denial	Data quality scoring, operating condition similarity calculation, model divergence analysis, and version tracing	Diagnostic passport: device identity, fault type, location, severity, evidence, confidence, applicability domain, and reason for abstention

**Table 7:** Minimum delivery fields and usage rules for agricultural machinery diagnostic passport.

Field Group	Minimum Delivery Field	Required Level	Suggested Expressions	Usage Rules	Denial/Audit Request
Object identity	Equipment number, model, components/interfaces; currently connected equipment	Proposed minimum field	Unique identifier + name + version	The conclusion is only valid for the marked equipment, structure, and connection relationship; re-confirm the applicability after replacing the implements or parts	Missing, conflicting, or unknown identity triggers review rather than automatic action
Current operating conditions	Task, field/environment, speed, load, job object, and operation phase	Proposed minimum field	Time window + classification field + key continuous quantity	Used to distinguish real-world faults, load mutations, and environmental effects, and define the conditions for establishing diagnostic conclusions	When the training/validation domain is exceeded or the key operating condition field is missing, the confidence level is lowered and marked as outside the domain
Diagnostic conclusion	Failure category, location/interface, status, and candidate root cause	Proposed minimum field	Controlled vocabulary encoding + human-readable names; candidate collections allowed	Anomaly detection, location, type identification, and severity tasks are output separately, and one task result is not substituted for another	Conflicting evidence or missing key measurements triggers a candidate set or abstention rather than forced classification
Severity	Degree of functional loss and impact on safety, quality, shutdown, and maintenance	Proposed task-condition field	Rank/continuous value + interval + boundary source	Used only when the task contains severity estimation or risk treatment; interpreted relative to the task profile	Unbounded sources, no intervals, or a simple average of the four consequence dimensions are not used for automated decision-making
Probability and intervals	Diagnostic probability, confidence interval, or uncertainty; calibration status	Proposed minimum field (at least one type)	probability/interval + calibration set + calibration date	Probability is used to express accuracy expectations rather than physical severity; also reports off-device/out-of-field/out-of-season validation ranges	Confidence is updated when input missing data, drift, out-of-domain, or model divergence increases; high-confidence errors are audited with focus
Source of evidence	Key variables, sensing sources, time windows, event logs, mechanism-based constraints, and counterevidence	Proposed minimum field	Evidence list + source identification + timestamp + inference path	The proposed explanation states what kind of evidence is used, what kind of physical object it corresponds to, and under what conditions it is established	Only showing “important characteristics” or directly writing accompanying symptoms such as temperature rise as root causes are considered insufficient evidence
Strength of evidence	Data quality, operating condition similarity, mechanism residuals, model differences	Recommended field	Itemized value + original amount + calculated version	Each sub-item is retained separately, and the comprehensive score that has not been calibrated by the target device is not used as a universal scale	Review/abstention is triggered when integrity, synchronization, drift, or physical consistency falls below the verified range
Applicable domain	Applicable models, machines, tasks, operating conditions, environment, and data conditions	Recommended field	Whitelist scope + verified boundary + out-of-domain flag	Conclusions are only reusable within explicitly applicability domains; application across devices, fields, and seasons requires independent verification	Out-of-domain samples trigger recalibration, review, or abstention before thresholds and probabilities are reused
Abstention conditions	Trigger conditions, reasons for abstention, follow-up actions, and responsible persons	Proposed minimum field	Rule encoding + readable reason + upgrade path	Priority will be given to abstention when there is insufficient evidence; manual review marks will be retained when there is strong physical evidence for safety-critical faults	Each abstention records the input status, triggering rules, time, and person responsible for receiving it. A bare “unknown” output is insufficient for audit
Latency and resources	Collection/computation/transmission delay, computing power, energy consumption, bandwidth, and storage budget	Proposed deployment-condition field	Measured distribution + hardware/software version	Used to determine whether diagnosis can meet the control, shutdown, or manual intervention time limit	When only average inference time is reported without collection, communication, and queuing overhead, it is not used as evidence of real-time deployment
Failure modes and versions	Known dead zones, sensor/link failure modes, model, and rule versions	Proposed deployment-condition field	Failure mode list + version number + update time	Generate a new passport version after changes to the model, thresholds, mechanism-based constraints, and data processing chain	Keep old versions, negative results, false positives/negatives, and revision reasons to ensure traceability of conclusions

**Table 8:** Case-evidence matrix for the illustrative composite mappings in Section 7.

Illustrative Composite Mapping	Closed-Loop Stages Covered	Directly Observed	Review Reconstruction	Reported Follow-Up	Missing Evidence	Interpretation
PTO shaft, front axle, and rotary-rake failures (Section 7.1)	Fault formation; boundary; repair target	Natural fractures/damage, loads, inspection, and consequences	Unified causal chain and boundary mapping	Rake: five machines/1500 cumulative h; no post-repair follow-up	Same-machine diagnosis, action, acceptance, and feedback	Mechanism/target evidence only
Combine, threshing rig, and powershift tractor (Section 7.2)	Sensing; quality control; detection/localization	Signals, injected states, pressure/vibration findings	Common data contract, diagnostic passport, and trigger	No longitudinal post-diagnosis follow-up	Natural faults, independent machine test, repair and recovery	Composite diagnostic illustration
Tractor oil, replacement model, and service scheduling (Section 7.3)	Degradation; forecasting; risk/resource decision	Oil series to 600 h, inspection, model, and scheduling outputs	Risk trajectory and work-order linkage	Sampling every 100 h to 600 h; no action-outcome follow-up	Prediction intervals, warning errors, and same-machine intervention	Composite prognostic decision illustration
Two-wheel tractor and plowshare repair (Section 7.4)	Review; repair; execution; acceptance	Disassembly, replacement/repair, and field/bench outcomes	Effectiveness labels and proposed knowledge write-back	Field test; 10 ha or ~200 acres in component studies; extended recurrence follow-up absent	Comparable baseline, long-term persistence and verified digital feedback	Composite repair–recovery illustration

**Table 9:** Evidence-oriented comparison of diagnostic and prognostic method families.

Method Family	Dataset and Fault Authenticity	Equipment Independence and Validation	Metrics	Uncertainty Handling	Deployment Evidence/Gap
Mechanism/expert/knowledge reasoning	Rules, topology, physics, and sparse observations; can encode natural mechanisms, though labels may be expert-derived	Test unseen machines/configurations; use contradiction and causal-consistency tests	Coverage, localization and rule conflicts	Rule confidence and unknown state handling	Useful for traceability; update burden and field validation should be reported
Engineered features + classical ML	Synchronized vibration/current/pressure/speed; faults are often injected or controlled	Split by machine/run rather than adjacent windows; use independent-condition and temporal holdout	Balanced class metrics, calibration and latency	Calibrated probability and abstention	Many bench/field prototypes; sustained deployment often absent
Deep representation learning	Larger raw or time-frequency datasets; natural faults are usually scarce	Require external machine/domain test with a locked model and independent test set	Class metrics, calibration, compute, and robustness	OOD detection, intervals, or ensembles	Edge cost and failure cases should accompany accuracy
Mechanism–data/control-aware fusion	Signals plus physical/control variables; authenticity depends on the underlying fault source	Test across dynamics, loads and controller versions; include ablation and physical-consistency checks	Detection, residual quality, false alarms, and latency	Residual/threshold history and applicability	Promising auditable interface; agricultural performance requires direct validation
Transfer/few-shot/domain adaptation	Source domains plus a small target set; target faults may remain sparse or artificial	Use an unseen target machine; isolate target labels used for tuning from the final test	Adaptation gain, calibration, and negative transfer	Domain distance, OOD, and abstention	Method transfer only until prospective target machine evidence exists
Prognostics/RUL	Independent degradation trajectories with future loads; natural degradation and an endpoint are preferred	Separate machines, fields, seasons, and time; use rolling-origin or prospective validation	MAE/RMSE, interval coverage and warning lead time	Prediction interval and threshold uncertainty	Direct agricultural evidence remains limited, especially after action

## Data Availability

The information analyzed in this review was obtained from the published literature cited in the article. As databases are continuously updated, the same search strategy may produce different results over time. The search strategy, literature selection process, and evidence synthesis methods are described in Section 2. No original experimental datasets were generated. The reported results reflect the database records available on the stated search dates.

## Supplementary Materials

The following supporting information can be downloaded at: https://www.mdpi.com/article/10.3390/s26175679/s1, S1: Search Strategy and Search Log; S2: Reconciled Selection Flow; S3: Proposed Study-Level Evidence Ledger; S4: Evidence Distribution Summary.
