# Supplementary material for: A Review of Fault Diagnosis and Intelligent Operations and Maintenance for Agricultural Machinery: Fault Mechanisms, Key Technologies, and Practical Recommendations
Source: Sensors (Basel). 2026 Sep 7;26(17):5679. doi: 10.3390/s26175679 (PMC13568347; doi:10.3390/s26175679)
Supplement: Supplementary file 1 [file sensors-26-05679-s001.zip › sensors-4531183-supplementary.pdf]

Supplementary Materials

Fault Diagnosis and Intelligent Operations and Maintenance for Agricultural Machinery: A Review

S1. Search Strategy and Search Log

Source	Coverage	Search date	Search approach	Records entered
Web of Science Core Collection	June 2016-June 2026	8 July 2026	Comprehensive and four task-specific Boolean queries	16,732
CNKI	June 2016-June 2026	8 July 2026	Chinese subject-field combinations for mechanism, diagnosis, prognosis, maintenance, and repair	1,635
Google Scholar	June 2016-June 2026	8 July 2026	Nine English short phrases plus Chinese equivalents; initial search displayed 18,508 results; first 100 pages reviewed; stopped at platform page-access limit	2,000 actually saved via Zotero Connector

Complete Web of Science queries:

Comprehensive: TS=(("agricultural machinery" OR tractor* OR "combine harvester*" OR seeder* OR planter* OR sprayer* OR "agricultural robot*" OR "agricultural UAV*") AND TS=(fault* OR failure* OR degradation OR wear OR fatigue OR corrosion) AND TS=(diagnos* OR detect* OR "condition monitoring" OR prognos* OR "remaining useful life" OR maintenance OR repair OR remanufactur*))

Fault mechanisms: TS=(("agricultural machinery" OR tractor* OR "combine harvester*") AND TS=(wear OR fatigue OR corrosion OR crack* OR "failure mechanism*" OR "fault propagation" OR "cascading failure*"))

Current-state diagnosis: TS=((tractor* OR harvester* OR seeder* OR sprayer* OR "agricultural robot*") AND TS=("fault detection" OR "fault diagnosis" OR "condition monitoring" OR "fault localization" OR ontology OR "knowledge graph" OR "physics informed"))

Prognostics: TS=(("agricultural machinery" OR tractor* OR harvester*) AND TS=(prognos* OR "health indicator" OR degradation OR "remaining useful life" OR "early warning" OR "predictive maintenance" OR uncertainty))

Repair and recovery: TS=(("agricultural machinery" OR tractor* OR harvester*) AND TS=(maintenance OR repair OR remanufactur* OR "spare part*" OR "maintenance service" OR "repair validation" OR "maintenance feedback"))

Complete CNKI Subject-field queries (SU):

Comprehensive: SU=("农业机械" OR 拖拉机 OR 联合收获机 OR 播种机 OR 喷雾机 OR 农业机器人 OR 农业无人机) AND SU=(故障 OR 失效 OR 退化 OR 磨损 OR 疲劳 OR 腐蚀) AND SU=(检测 OR 诊断 OR 状态监测 OR 预测 OR 剩余使用寿命 OR 维护 OR 维修 OR 再制造)

Fault mechanisms: SU=("农业机械" OR 拖拉机 OR 联合收获机) AND SU=(磨损 OR 疲劳 OR 腐蚀 OR 裂纹 OR 故障机理 OR 失效机理 OR 故障传播 OR 级联故障)

Current-state diagnosis: SU=(拖拉机 OR 联合收获机 OR 播种机 OR 喷雾机 OR 农业机器人) AND SU=(故障检测 OR 故障诊断 OR 状态监测 OR 故障定位 OR 知识图谱 OR 机理模型)

Prognostics and early warning: SU=("农业机械" OR 拖拉机 OR 联合收获机) AND SU=(故障预测 OR 趋势预测 OR 健康指标 OR 性能退化 OR 剩余使用寿命 OR 预警 OR 预测性维护 OR 不确定性)

Maintenance and recovery: SU=("农业机械" OR 拖拉机 OR 联合收获机) AND SU=(维护 OR 维修 OR 修复 OR 再制造 OR 备件 OR 维修服务 OR 维修质量 OR 修复验证 OR 维修反馈)

Google Scholar phrases: agricultural machinery fault diagnosis; tractor gearbox condition monitoring; combine harvester fault prediction; agricultural machinery remaining useful life; agricultural machinery predictive maintenance; agricultural machinery repair remanufacturing; seeder missed-seeding detection; agricultural UAV flow fault; agricultural robot fault-tolerant control; plus Chinese equivalents.

S2. Reconciled Selection Flow

Stage	Count	Notes
Records entered into Zotero	20,367	WoS 16,732; CNKI 1,635; Google Scholar initially displayed 18,508 results and 2,000 were actually saved
Duplicates removed	2,815	Final flow count
Title/abstract records screened	17,552	Combined library; downstream counts not retained by database
Topically irrelevant records excluded	16,833	Title/abstract stage
Full texts assessed	719	17,552 − 16,833 = 719
Full texts excluded	524	128 insufficient technical information; 39 unavailable full texts; 32 duplicate study results; 325 outside the core research tasks
Included after full-text eligibility	195	719 − 524 = 195
Final qualitative synthesis	195	Final total

S3. Proposed Study-Level Evidence Ledger

This ledger presents a conservative full-text classification of all 195 corresponding PDFs. Level 1 denotes simulation, laboratory, bench, injected-fault, fixed-condition, or public-benchmark evidence; Level 2 denotes single-machine or single-site field/in-service evidence; Level 3 denotes evidence across independent machines, plots, sites, regions, companies, or seasons; and Level 4 denotes end-to-end operational evidence in which a defined diagnostic, maintenance, or refurbishment method was applied in a real operating or maintenance context, followed by an actual inspection, repair, replacement, or other intervention and verification through post-intervention performance, repair time, cost, availability, or operational outcomes. All 195 included publications were classified using the same Level 1-4 framework. 'Not reported' means that the field remained unverifiable in the PDF full text; it does not prove that the original study did not record it. Reference numbers follow the manuscript's 200-item reference list. References 140-144 are two ISO standards and three regulatory sources outside the 195-publication research-evidence ledger; therefore, study records 1-139 retain their manuscript numbers, while study records 140-195 correspond to manuscript references 145-200. Primary-stage labels follow the manuscript's separated prognostics, risk-control, maintenance-and-repair, and post-repair-verification stages. The item-level distribution is 24 fault-mechanism, 88 current-state-diagnosis, 24 prognostics, 14 risk-control, 10 post-repair-verification, 20 maintenance-and-repair, 8 illustrative mechanism/diagnosis, and 7 illustrative prognostics/repair publications, totaling 195.

Manuscript ref.	Source	Primary stage	Level	Setting	Fault authenticity	Independent machines	Intervention	Follow-up	Confidence
1	Direct agricultural evidence	Fault mechanisms and system boundaries	Level 1	Not reported	Not reported	Not reported	Not reported	Not reported	High
2	Direct agricultural evidence	Fault mechanisms and system boundaries	Level 1	Not reported	Not reported	Not reported	Not reported	Not reported	High
3	Direct agricultural evidence	Fault mechanisms and system boundaries	Level 1	Not reported	Not reported	Not reported	Not reported	Not reported	High
4	Direct agricultural evidence	Fault mechanisms and system boundaries	Level 1	Not reported	Not reported	Not reported	Not reported	Not reported	High
5	Cross-industry methodological evidence	Fault mechanisms and system boundaries	Level 1	Not reported	Not reported	Not reported	Not reported	Not reported	Medium

Manuscript ref.	Source	Primary stage	Level	Setting	Fault authenticity	Independent machines	Intervention	Follow-up	Confidence
6	Direct agricultural evidence	Fault mechanisms and system boundaries	Level 1	Not reported	Not reported	Not reported	Not reported	Not reported	Medium
7	Direct agricultural evidence	Fault mechanisms and system boundaries	Level 1	Not reported	Not reported	Not reported	Intervention/repair discussed or implemented	Not reported	High
8	Cross-industry methodological evidence	Fault mechanisms and system boundaries	Level 1	Not reported	Not reported	Not reported	Not reported	Not reported	High
9	Direct agricultural evidence	Fault mechanisms and system boundaries	Level 2	Simulation	Not reported	Not reported	Intervention/repair discussed or implemented	Not reported	High
10	Direct agricultural evidence	Fault mechanisms and system boundaries	Level 1	Not reported	Not reported	Not reported	Not reported	Not reported	High
11	Cross-industry methodological evidence	Fault mechanisms and system boundaries	Level 1	Laboratory / controlled experiment	Not reported	1 physical prototype	Not reported	Not reported	High
12	Direct agricultural evidence	Fault mechanisms and system boundaries	Level 1	Not reported	Not reported	Not reported	Not reported	Not reported	High
13	Direct agricultural evidence	Fault mechanisms and system boundaries	Level 2	Field / in-service	Not reported	1 or not independently replicated	Not reported	Not reported	High
14	Direct agricultural evidence	Fault mechanisms and system boundaries	Level 2	Simulation	Not reported	Not reported	Not reported	Not reported	High
15	Cross-industry methodological evidence	Fault mechanisms and system boundaries	Level 2	Simulation	Not reported	Not reported	Not reported	Not reported	High
16	Direct agricultural evidence	Fault mechanisms and system boundaries	Level 1	Simulation	Not reported	Not reported	Not reported	Not reported	High
17	Direct agricultural evidence	Fault mechanisms and system boundaries	Level 1	Not reported	Not reported	Not reported	Not reported	Not reported	High
18	Direct agricultural evidence	Fault mechanisms and system boundaries	Level 1	Simulation	Not reported	Not reported	Not reported	Not reported	Medium
19	Direct agricultural evidence	Fault mechanisms and system boundaries	Level 2	Field / in-service	Not reported	1 or not independently replicated	Not reported	Not reported	High
20	Direct agricultural evidence	Fault mechanisms and system boundaries	Level 2	Field / in-service	Not reported	1 tractor-rotary-cultivator combination	Not reported	Not reported	High

Manuscript ref.	Source	Primary stage	Level	Setting	Fault authenticity	Independent machines	Intervention	Follow-up	Confidence
21	Cross-industry methodological evidence	Fault mechanisms and system boundaries	Level 1	Not reported	Not reported	Not reported	Not reported	Not reported	High
22	Cross-industry methodological evidence	Fault mechanisms and system boundaries	Level 1	Not reported	Not reported	Not reported	Not reported	Not reported	High
23	Cross-industry methodological evidence	Fault mechanisms and system boundaries	Level 1	Not reported	Not reported	Not reported	Not reported	Not reported	High
24	Direct agricultural evidence	Fault mechanisms and system boundaries	Level 1	Not reported	Not reported	Not reported	Not reported	Not reported	High
25	Direct agricultural evidence	Current state diagnosis and trustworthy delivery	Level 1	Laboratory / controlled experiment	Natural/in-service failure or degradation	Not reported	Not reported	Not reported	High
26	Cross-industry methodological evidence	Current state diagnosis and trustworthy delivery	Level 1	Not reported	Not reported	Not reported	Not reported	Not reported	High
27	Direct agricultural evidence	Current state diagnosis and trustworthy delivery	Level 1	Not reported	Not reported	Not reported	Not reported	Not reported	Medium
28	Direct agricultural evidence	Current state diagnosis and trustworthy delivery	Level 1	Bench / test rig	Not reported	Not reported	Not reported	Not reported	High
29	Direct agricultural evidence	Current state diagnosis and trustworthy delivery	Level 1	Bench / test rig	Not reported	Not reported	Not reported	Not reported	Medium
30	Direct agricultural evidence	Current state diagnosis and trustworthy delivery	Level 1	Not reported	Not reported	Not reported	Not reported	Not reported	Medium
31	Direct agricultural evidence	Current state diagnosis and trustworthy delivery	Level 1	Not reported	Not reported	Not reported	Not reported	Not reported	High
32	Cross-industry methodological evidence	Current state diagnosis and trustworthy delivery	Level 1	Not reported	Not reported	Not reported	Not reported	Not reported	High
33	Direct agricultural evidence	Current state diagnosis and trustworthy delivery	Level 1	Field / in-service	Not reported	Not reported	Not reported	Not reported	High
34	Direct agricultural evidence	Current state diagnosis and trustworthy delivery	Level 1	Not reported	Not reported	Not reported	Not reported	Not reported	High
35	Direct agricultural evidence	Current state diagnosis and trustworthy delivery	Level 1	Laboratory / controlled experiment	Not reported	Not reported	Intervention/repair discussed or implemented	Not reported	Medium

Manuscript ref.	Source	Primary stage	Level	Setting	Fault authenticity	Independent machines	Intervention	Follow-up	Confidence
36	Direct agricultural evidence	Current state diagnosis and trustworthy delivery	Level 1	Laboratory / controlled experiment	Not reported	Not reported	Not reported	Not reported	High
37	Cross-industry methodological evidence	Current state diagnosis and trustworthy delivery	Level 1	Not reported	Not reported	Not reported	Not reported	Not reported	High
38	Direct agricultural evidence	Current state diagnosis and trustworthy delivery	Level 2	Simulation	Not reported	Not reported	Not reported	Not reported	Medium
39	Cross-industry methodological evidence	Current state diagnosis and trustworthy delivery	Level 1	Bench / test rig	Not reported	Not reported	Not reported	Not reported	Medium
40	Cross-industry methodological evidence	Current state diagnosis and trustworthy delivery	Level 2	Field / in-service	Not reported	1 greenhouse/site	Not reported	Not reported	High
41	Direct agricultural evidence	Current state diagnosis and trustworthy delivery	Level 1	Not reported	Not reported	Not reported	Not reported	Not reported	High
42	Direct agricultural evidence	Current state diagnosis and trustworthy delivery	Level 1	Not reported	Not reported	Not reported	Not reported	Not reported	Medium
43	Direct agricultural evidence	Current state diagnosis and trustworthy delivery	Level 1	Not reported	Not reported	Not reported	Not reported	Not reported	High
44	Direct agricultural evidence	Current state diagnosis and trustworthy delivery	Level 1	Not reported	Not reported	Not reported	Not reported	Not reported	Medium
45	Direct agricultural evidence	Current state diagnosis and trustworthy delivery	Level 1	Not reported	Not reported	Not reported	Not reported	Not reported	High
46	Direct agricultural evidence	Current state diagnosis and trustworthy delivery	Level 1	Not reported	Not reported	Not reported	Not reported	Not reported	High
47	Cross-industry methodological evidence	Current state diagnosis and trustworthy delivery	Level 1	Not reported	Not reported	Not reported	Not reported	Not reported	High
48	Direct agricultural evidence	Current state diagnosis and trustworthy delivery	Level 1	Simulation	Not reported	Not reported	Not reported	Not reported	Medium
49	Direct agricultural evidence	Current state diagnosis and trustworthy delivery	Level 1	Not reported	Not reported	Not reported	Not reported	Not reported	High
50	Direct agricultural evidence	Current state diagnosis and trustworthy delivery	Level 2	Simulation	Not reported	Not reported	Not reported	Not reported	Medium

Manuscript ref.	Source	Primary stage	Level	Setting	Fault authenticity	Independent machines	Intervention	Follow-up	Confidence
51	Direct agricultural evidence	Current state diagnosis and trustworthy delivery	Level 1	Not reported	Not reported	Not reported	Not reported	Not reported	Medium
52	Direct agricultural evidence	Current state diagnosis and trustworthy delivery	Level 1	Laboratory / controlled experiment	Not reported	Not reported	Not reported	Not reported	Medium
53	Direct agricultural evidence	Current state diagnosis and trustworthy delivery	Level 2	Simulation	Not reported	Not reported	Not reported	Not reported	Medium
54	Direct agricultural evidence	Current state diagnosis and trustworthy delivery	Level 1	Simulation	Not reported	Not reported	Not reported	Not reported	Medium
55	Direct agricultural evidence	Current state diagnosis and trustworthy delivery	Level 2	Simulation	Not reported	Not reported	Not reported	Not reported	Medium
56	Cross-industry methodological evidence	Current state diagnosis and trustworthy delivery	Level 2	Simulation	Not reported	Not reported	Not reported	Not reported	Medium
57	Direct agricultural evidence	Current state diagnosis and trustworthy delivery	Level 2	Bench / test rig	Not reported	Not reported	Not reported	Not reported	Medium
58	Direct agricultural evidence	Current state diagnosis and trustworthy delivery	Level 2	Laboratory / controlled experiment	Not reported	Not reported	Not reported	Not reported	Medium
59	Cross-industry methodological evidence	Current state diagnosis and trustworthy delivery	Level 2	Simulation	Not reported	Not reported	Not reported	Not reported	Medium
60	Direct agricultural evidence	Current state diagnosis and trustworthy delivery	Level 2	Field / in-service	Not reported	1 prototype at 1 field site	Intervention/repair discussed or implemented	Not reported	High
61	Direct agricultural evidence	Current state diagnosis and trustworthy delivery	Level 1	Not reported	Not reported	Not reported	Not reported	Not reported	High
62	Cross-industry methodological evidence	Current state diagnosis and trustworthy delivery	Level 1	Not reported	Not reported	Not reported	Not reported	Not reported	Medium
63	Direct agricultural evidence	Current state diagnosis and trustworthy delivery	Level 1	Not reported	Not reported	Not reported	Not reported	Not reported	High
64	Cross-industry methodological evidence	Current state diagnosis and trustworthy delivery	Level 1	Not reported	Not reported	Not reported	Not reported	Not reported	High
65	Cross-industry methodological evidence	Current state diagnosis and trustworthy delivery	Level 1	Laboratory / controlled experiment	Natural/in-service failure or degradation	Not reported	Not reported	Not reported	High

Manuscript ref.	Source	Primary stage	Level	Setting	Fault authenticity	Independent machines	Intervention	Follow-up	Confidence
66	Cross-industry methodological evidence	Current state diagnosis and trustworthy delivery	Level 1	Simulation	Not reported	Not reported	Not reported	Not reported	High
67	Cross-industry methodological evidence	Current state diagnosis and trustworthy delivery	Level 1	Simulation	Not reported	Not reported	Not reported	Not reported	High
68	Cross-industry methodological evidence	Current state diagnosis and trustworthy delivery	Level 1	Not reported	Natural/in-service failure or degradation	Not reported	Not reported	Not reported	High
69	Cross-industry methodological evidence	Current state diagnosis and trustworthy delivery	Level 1	Simulation	Not reported	Not reported	Not reported	Not reported	High
70	Cross-industry methodological evidence	Current state diagnosis and trustworthy delivery	Level 1	Not reported	Not reported	Not reported	Not reported	Not reported	Medium
71	Cross-industry methodological evidence	Current state diagnosis and trustworthy delivery	Level 1	Bench / test rig	Not reported	Not reported	Not reported	Not reported	High
72	Cross-industry methodological evidence	Current state diagnosis and trustworthy delivery	Level 1	Not reported	Not reported	Not reported	Not reported	Not reported	High
73	Direct agricultural evidence	Current state diagnosis and trustworthy delivery	Level 3	Multi-machine / multi-site field or operational evidence	Not reported	5 test plots	Not reported	Not reported	High
74	Direct agricultural evidence	Current state diagnosis and trustworthy delivery	Level 2	Field / in-service	Not reported	1 or not independently replicated	Not reported	Not reported	High
75	Cross-industry methodological evidence	Current state diagnosis and trustworthy delivery	Level 1	Not reported	Not reported	Not reported	Not reported	Not reported	Medium
76	Direct agricultural evidence	Current state diagnosis and trustworthy delivery	Level 2	Field / in-service	Not reported	1 or not independently replicated	Not reported	Not reported	High
77	Direct agricultural evidence	Current state diagnosis and trustworthy delivery	Level 1	Not reported	Not reported	Not reported	Not reported	Not reported	High
78	Direct agricultural evidence	Current state diagnosis and trustworthy delivery	Level 1	Not reported	Not reported	Not reported	Not reported	Not reported	Medium
79	Direct agricultural evidence	Current state diagnosis and trustworthy delivery	Level 1	Not reported	Not reported	Not reported	Not reported	Not reported	Medium
80	Direct agricultural evidence	Current state diagnosis and trustworthy delivery	Level 1	Not reported	Not reported	Not reported	Not reported	Not reported	Medium

Manuscript ref.	Source	Primary stage	Level	Setting	Fault authenticity	Independent machines	Intervention	Follow-up	Confidence
81	Cross-industry methodological evidence	Current state diagnosis and trustworthy delivery	Level 3	Multi-site field-origin evidence	Not reported	3 orchards in 3 regions; 276 apple samples	Not reported	Not reported	High
82	Direct agricultural evidence	Current state diagnosis and trustworthy delivery	Level 2	Field / in-service	Not reported	1 or not independently replicated	Not reported	Not reported	High
83	Direct agricultural evidence	Current state diagnosis and trustworthy delivery	Level 2	Field / in-service	Not reported	1 tea-farm study site	Not reported	Not reported	High
84	Direct agricultural evidence	Current state diagnosis and trustworthy delivery	Level 3	Multi-plot / multi-context field evidence	Not reported	125 plots; separate replication groups for training and validation	Not reported	Not reported	High
85	Direct agricultural evidence	Current state diagnosis and trustworthy delivery	Level 2	Field / in-service	Not reported	1 navigation platform/site	Not reported	Not reported	High
86	Direct agricultural evidence	Current state diagnosis and trustworthy delivery	Level 1	Not reported	Not reported	Not reported	Not reported	Not reported	High
87	Direct agricultural evidence	Current state diagnosis and trustworthy delivery	Level 2	Field / in-service	Not reported	Not reported	Not reported	Not reported	Medium
88	Cross-industry methodological evidence	Current state diagnosis and trustworthy delivery	Level 3	Multi-site / multi-season operational evidence	Not reported	2 cities and seasons: Wuxi-spring and Yancheng-autumn	Intervention/repair discussed or implemented	Not reported	High
89	Cross-industry methodological evidence	Current state diagnosis and trustworthy delivery	Level 1	Not reported	Not reported	Not reported	Not reported	Not reported	High
90	Cross-industry methodological evidence	Current state diagnosis and trustworthy delivery	Level 2	Field / in-service	Not reported	1 fertilization system/site	Not reported	Not reported	High
91	Direct agricultural evidence	Current state diagnosis and trustworthy delivery	Level 2	Field / in-service	Not reported	Not reported	Not reported	Not reported	Medium
92	Cross-industry methodological evidence	Current state diagnosis and trustworthy delivery	Level 1	Not reported	Not reported	Not reported	Not reported	Not reported	High
93	Cross-industry methodological evidence	Current state diagnosis and trustworthy delivery	Level 1	Not reported	Not reported	Not reported	Not reported	Not reported	High
94	Cross-industry methodological evidence	Current state diagnosis and trustworthy delivery	Level 1	Not reported	Not reported	Not reported	Not reported	Not reported	High
95	Cross-industry methodological evidence	Current state diagnosis and trustworthy delivery	Level 1	Not reported	Not reported	Not reported	Not reported	Not reported	High

Manuscript ref.	Source	Primary stage	Level	Setting	Fault authenticity	Independent machines	Intervention	Follow-up	Confidence
96	Cross-industry methodological evidence	Current state diagnosis and trustworthy delivery	Level 1	Not reported	Not reported	Not reported	Intervention/repair discussed or implemented	Not reported	High
97	Cross-industry methodological evidence	Current state diagnosis and trustworthy delivery	Level 1	Not reported	Not reported	Not reported	Intervention/repair discussed or implemented	Not reported	High
98	Cross-industry methodological evidence	Current state diagnosis and trustworthy delivery	Level 1	Not reported	Not reported	Not reported	Not reported	Not reported	High
99	Cross-industry methodological evidence	Current state diagnosis and trustworthy delivery	Level 1	Laboratory / controlled experiment	Not reported	Not reported	Not reported	Not reported	High
100	Cross-industry methodological evidence	Current state diagnosis and trustworthy delivery	Level 1	Not reported	Not reported	Not reported	Not reported	Not reported	Medium
101	Cross-industry methodological evidence	Current state diagnosis and trustworthy delivery	Level 1	Not reported	Not reported	Not reported	Not reported	Not reported	High
102	Cross-industry methodological evidence	Current state diagnosis and trustworthy delivery	Level 1	Not reported	Not reported	Not reported	Not reported	Not reported	High
103	Cross-industry methodological evidence	Current state diagnosis and trustworthy delivery	Level 1	Not reported	Not reported	Not reported	Not reported	Not reported	Medium
104	Cross-industry methodological evidence	Current state diagnosis and trustworthy delivery	Level 1	Not reported	Not reported	Not reported	Not reported	Not reported	High
105	Cross-industry methodological evidence	Current state diagnosis and trustworthy delivery	Level 1	Simulation	Injected actuator faults	Single nonlinear UAV simulation model	Adaptive threshold detection	Post-detection threshold state retained	High
106	Cross-industry methodological evidence	Current state diagnosis and trustworthy delivery	Level 1	Not reported	Not reported	Not reported	Not reported	Not reported	High
107	Direct agricultural evidence	Current state diagnosis and trustworthy delivery	Level 2	Field / in-service	Not reported	1 agricultural orchard; ancillary campus/external datasets	Not reported	Not reported	High
108	Direct agricultural evidence	Current state diagnosis and trustworthy delivery	Level 2	Simulation	Not reported	Not reported	Not reported	Not reported	Medium
109	Direct agricultural evidence	Current state diagnosis and trustworthy delivery	Level 2	Field / in-service	Not reported	Not reported	Not reported	Not reported	Medium
110	Direct agricultural evidence	Current state diagnosis and trustworthy delivery	Level 2	Field / in-service	Not reported	1 wheat-field site	Not reported	Not reported	High

Manuscript ref.	Source	Primary stage	Level	Setting	Fault authenticity	Independent machines	Intervention	Follow-up	Confidence
111	Cross-industry methodological evidence	Current state diagnosis and trustworthy delivery	Level 1	Not reported	Not reported	Not reported	Not reported	Not reported	Medium
112	Cross-industry methodological evidence	Current state diagnosis and trustworthy delivery	Level 1	Not reported	Not reported	Not reported	Not reported	Not reported	High
113	Cross-industry methodological evidence	Risk control	Level 1	Not reported	Not reported	Not reported	Intervention/repair discussed or implemented	Not reported	High
114	Cross-industry methodological evidence	Prognostics	Level 1	Not reported	Not reported	Not reported	Not reported	Not reported	High
115	Cross-industry methodological evidence	Prognostics	Level 1	Not reported	Not reported	Not reported	Not reported	Not reported	High
116	Cross-industry methodological evidence	Prognostics	Level 1	Not reported	Not reported	Not reported	Not reported	Not reported	High
117	Cross-industry methodological evidence	Prognostics	Level 1	Not reported	Not reported	Not reported	Not reported	Not reported	Medium
118	Cross-industry methodological evidence	Prognostics	Level 1	Not reported	Not reported	Not reported	Not reported	Not reported	High
119	Cross-industry methodological evidence	Prognostics	Level 1	Not reported	Not reported	Not reported	Not reported	Not reported	High
120	Cross-industry methodological evidence	Prognostics	Level 1	Not reported	Not reported	Not reported	Not reported	Not reported	High
121	Cross-industry methodological evidence	Prognostics	Level 1	Not reported	Not reported	Not reported	Not reported	Not reported	High
122	Cross-industry methodological evidence	Prognostics	Level 1	Not reported	Not reported	Not reported	Not reported	Not reported	High
123	Cross-industry methodological evidence	Prognostics	Level 1	Not reported	Not reported	Not reported	Not reported	Not reported	High
124	Cross-industry methodological evidence	Prognostics	Level 1	Not reported	Not reported	Not reported	Not reported	Not reported	High
125	Cross-industry methodological evidence	Prognostics	Level 1	Not reported	Not reported	Not reported	Not reported	Not reported	High

Manuscript ref.	Source	Primary stage	Level	Setting	Fault authenticity	Independent machines	Intervention	Follow-up	Confidence
126	Cross-industry methodological evidence	Prognostics	Level 1	Not reported	Not reported	Not reported	Not reported	Not reported	High
127	Cross-industry methodological evidence	Prognostics	Level 1	Bench / test rig	Not reported	Not reported	Not reported	different noise environments with minimal amount o	High
128	Cross-industry methodological evidence	Prognostics	Level 1	Not reported	Not reported	Not reported	Not reported	Not reported	High
129	Cross-industry methodological evidence	Prognostics	Level 1	Not reported	Not reported	Not reported	Not reported	Not reported	Medium
130	Cross-industry methodological evidence	Prognostics	Level 1	Not reported	Not reported	Not reported	Not reported	Not reported	High
131	Cross-industry methodological evidence	Prognostics	Level 1	Not reported	Not reported	Not reported	Not reported	Not reported	Medium
132	Cross-industry methodological evidence	Prognostics	Level 1	Laboratory / controlled experiment	Not reported	Not reported	Not reported	Not reported	High
133	Cross-industry methodological evidence	Prognostics	Level 1	Not reported	Not reported	Not reported	Intervention/repair discussed or implemented	Not reported	High
134	Direct agricultural evidence	Prognostics	Level 1	Not reported	Not reported	Not reported	Not reported	Not reported	High
135	Direct agricultural evidence	Prognostics	Level 1	Not reported	Not reported	Not reported	Not reported	Not reported	High
136	Direct agricultural evidence	Prognostics	Level 1	Not reported	Not reported	Not reported	Not reported	Not reported	High
137	Cross-industry methodological evidence	Risk control	Level 1	Not reported	Not reported	Not reported	Not reported	Not reported	High
138	Cross-industry methodological evidence	Risk control	Level 3	Multi-machine / multi-site field or operational evidence	Not reported	7 industrial case studies (2017–2024)	Not reported	Not reported	High
139	Cross-industry methodological evidence	Risk control	Level 1	Not reported	Not reported	Not reported	Not reported	Not reported	High
145	Cross-industry methodological evidence	Risk control	Level 1	Laboratory / controlled experiment	Not reported	Not reported	Intervention/repair discussed or implemented	Not reported	High

Manuscript ref.	Source	Primary stage	Level	Setting	Fault authenticity	Independent machines	Intervention	Follow-up	Confidence
146	Cross-industry methodological evidence	Risk control	Level 2	Simulation	Not reported	Not reported	Not reported	Not reported	Medium
147	Direct agricultural evidence	Risk control	Level 2	Simulation	Not reported	Not reported	Not reported	Not reported	High
148	Cross-industry methodological evidence	Risk control	Level 1	Not reported	Not reported	Not reported	Not reported	Not reported	Medium
149	Cross-industry methodological evidence	Risk control	Level 2	Field / in-service	Not reported	1 company; 5 cutting tools; 3 operating conditions	Not reported	Not reported	High
150	Direct agricultural evidence	Prognostics	Level 1	Not reported	Not reported	Not reported	Not reported	Not reported	High
151	Cross-industry methodological evidence	Risk control	Level 1	Not reported	Not reported	Not reported	Not reported	Not reported	High
152	Cross-industry methodological evidence	Risk control	Level 1	Not reported	Not reported	Not reported	Not reported	Not reported	High
153	Cross-industry methodological evidence	Risk control	Level 1	Not reported	Not reported	Not reported	Not reported	Not reported	High
154	Direct agricultural evidence	Risk control	Level 2	Field / in-service	Not reported	1 or not independently replicated	Not reported	Not reported	High
155	Cross-industry methodological evidence	Risk control	Level 1	Not reported	Not reported	Not reported	Not reported	Not reported	High
156	Direct agricultural evidence	Maintenance and repair	Level 1	Not reported	Not reported	Not reported	Not reported	Not reported	High
157	Cross-industry methodological evidence	Maintenance and repair	Level 1	Not reported	Not reported	Not reported	Not reported	Not reported	High
158	Cross-industry methodological evidence	Maintenance and repair	Level 1	Not reported	Natural/in-service failure or degradation	Not reported	Not reported	Not reported	High
159	Cross-industry methodological evidence	Maintenance and repair	Level 1	Not reported	Not reported	Not reported	Intervention/repair discussed or implemented	Not reported	High
160	Cross-industry methodological evidence	Maintenance and repair	Level 2	Field / in-service	Natural/in-service failure or degradation	2 manufacturing companies; qualitative application	Intervention/repair discussed or implemented	Not reported	High

Manuscript ref.	Source	Primary stage	Level	Setting	Fault authenticity	Independent machines	Intervention	Follow-up	Confidence
161	Direct agricultural evidence	Maintenance and repair	Level 1	Not reported	Natural/in-service failure or degradation	Not reported	Intervention/repair discussed or implemented	Not reported	Medium
162	Cross-industry methodological evidence	Maintenance and repair	Level 1	Not reported	Not reported	Not reported	Not reported	Not reported	High
163	Cross-industry methodological evidence	Maintenance and repair	Level 1	Not reported	Not reported	Not reported	Not reported	Not reported	High
164	Cross-industry methodological evidence	Post-repair verification	Level 1	Not reported	Not reported	Not reported	Not reported	Not reported	High
165	Cross-industry methodological evidence	Maintenance and repair	Level 1	Not reported	Not reported	Not reported	Not reported	Not reported	High
166	Cross-industry methodological evidence	Maintenance and repair	Level 2	Field / in-service	Not reported	1 company; 10,843 spare parts; 3 sequential projects	Intervention/repair discussed or implemented	Not reported	High
167	Cross-industry methodological evidence	Maintenance and repair	Level 1	Not reported	Not reported	Not reported	Not reported	Not reported	High
168	Cross-industry methodological evidence	Maintenance and repair	Level 1	Not reported	Not reported	Not reported	Intervention/repair discussed or implemented	Not reported	High
169	Cross-industry methodological evidence	Maintenance and repair	Level 1	Not reported	Not reported	Not reported	Not reported	Not reported	High
170	Direct agricultural evidence	Maintenance and repair	Level 2	Field / in-service	Not reported	1 or not independently replicated	Not reported	Not reported	High
171	Direct agricultural evidence	Maintenance and repair	Level 3	Multi-machine / multi-site field or operational evidence	Not reported	3 provinces; 251 service units	Not reported	Not reported	High
172	Direct agricultural evidence	Maintenance and repair	Level 3	Multi-region operational evidence	Not reported	4,905 farmers in 10 provinces	Intervention/repair discussed or implemented	Not reported	High
173	Cross-industry methodological evidence	Maintenance and repair	Level 2	Field / in-service	Not reported	1 or not independently replicated	Intervention/repair discussed or implemented	Not reported	High
174	Cross-industry methodological evidence	Post-repair verification	Level 4	Multi-company operational evidence	Not reported	projects verified in several independent companies	Maintenance-strategy changes were implemented through company projects	Post-implementation operational indicators reported; individual project durations not reported	High

Manuscript ref.	Source	Primary stage	Level	Setting	Fault authenticity	Independent machines	Intervention	Follow-up	Confidence
175	Direct agricultural evidence	Post-repair verification	Level 1	Not reported	Not reported	Not reported	Not reported	Not reported	Medium
176	Direct agricultural evidence	Post-repair verification	Level 2	Field / in-service	Not reported	1 tractor-mounted sprayer/site	Not reported	Not reported	High
177	Direct agricultural evidence	Post-repair verification	Level 1	Simulation	Not reported	Not reported	Not reported	Not reported	Medium
178	Direct agricultural evidence	Post-repair verification	Level 1	Not reported	Not reported	Not reported	Not reported	Not reported	Medium
179	Direct agricultural evidence	Post-repair verification	Level 2	Field / in-service	Not reported	Not reported	Not reported	Not reported	Medium
180	Cross-industry methodological evidence	Post-repair verification	Level 1	Not reported	Not reported	Not reported	Not reported	Not reported	Medium
181	Direct agricultural evidence	Maintenance and repair	Level 3	Multi-season field/greenhouse evidence	Not reported	2 planting seasons; 4 replications per season	Not reported	Not reported	High
182	Cross-industry methodological evidence	Post-repair verification	Level 1	Not reported	Not reported	Not reported	Not reported	Not reported	High
183	Cross-industry methodological evidence	Post-repair verification	Level 1	Not reported	Not reported	Not reported	Not reported	Not reported	High
184	Direct agricultural evidence	Maintenance and repair	Level 1	Not reported	Not reported	Not reported	Not reported	Not reported	High
185	Direct agricultural evidence	Maintenance and repair	Level 1	Not reported	Not reported	Not reported	Not reported	Not reported	Medium
186	Direct agricultural evidence	Illustrative mechanism/diagnosis cases	Level 2	Field / in-service	Natural/in-service failure or degradation	1 or not independently replicated	Not reported	Not reported	High
187	Direct agricultural evidence	Illustrative mechanism/diagnosis cases	Level 2	Field / in-service	Natural/in-service failure or degradation	1 or not independently replicated	Not reported	Not reported	High
188	Direct agricultural evidence	Illustrative mechanism/diagnosis cases	Level 1	Not reported	Not reported	Not reported	Not reported	Not reported	Medium
189	Cross-industry methodological evidence	Illustrative mechanism/diagnosis cases	Level 1	Bench / test rig	Not reported	Not reported	Intervention/repair discussed or implemented	Not reported	High

Manuscript ref.	Source	Primary stage	Level	Setting	Fault authenticity	Independent machines	Intervention	Follow-up	Confidence
190	Direct agricultural evidence	Illustrative mechanism/diagnosis cases	Level 4	Field / in-service	Not reported	1 T8 agricultural tractor	Five software-identified transmission/clutch-system parts were actually replaced by the maintenance team	Immediate post-repair time, cost, and availability evaluation; extended follow-up not reported	High
191	Direct agricultural evidence	Illustrative prognostics/repair cases	Level 4	Multi-machine longitudinal operational evidence	Not reported	3 tractors; sampling every 100 h through 600 h	Physical inspection after 600 operating hours verified the source of the detected anomaly	600 operating hours with samples every 100 hours and terminal physical inspection	High
192	Direct agricultural evidence	Illustrative mechanism/diagnosis cases	Level 1	Not reported	Not reported	Not reported	Not reported	Not reported	Medium
193	Direct agricultural evidence	Illustrative mechanism/diagnosis cases	Level 2	Field / in-service	Not reported	1 or not independently replicated	Not reported	Not reported	High
194	Direct agricultural evidence	Illustrative mechanism/diagnosis cases	Level 3	Multi-machine / multi-site field or operational evidence	Natural/in-service failure or degradation	5 tractor-raker combinations; 1,500 cumulative field hours	Not reported	Not reported	High
195	Cross-industry methodological evidence	Illustrative prognostics/repair cases	Level 1	Not reported	Not reported	Not reported	Not reported	Not reported	Medium
196	Direct agricultural evidence	Illustrative prognostics/repair cases	Level 1	Not reported	Not reported	Not reported	Intervention/repair discussed or implemented	Not reported	High
197	Direct agricultural evidence	Illustrative prognostics/repair cases	Level 1	Laboratory / controlled experiment	Not reported	Not reported	Not reported	Not reported	High
198	Direct agricultural evidence	Illustrative prognostics/repair cases	Level 4	Field / in-service	Natural/in-service failure or degradation	1 refurbished two-wheeled tractor	Carburetor, ignition coil, tyres, and other worn parts were replaced or repaired, and an implement-coupling flange was fabricated	Post-refurbishment field performance test; extended follow-up not reported	High
199	Direct agricultural evidence	Illustrative prognostics/repair cases	Level 1	Not reported	Not reported	Not reported	Not reported	Not reported	Medium
200	Direct agricultural evidence	Illustrative prognostics/repair cases	Level 1	Bench / test rig	Not reported	Not reported	Intervention/repair discussed or implemented	Not reported	High

S4. Evidence Distribution Summary

Category	Count
Level 1	141
Level 2	41
Level 3	9
Level 4	4
Cross-industry methodological evidence	98
Direct agricultural evidence	97

After full-text review, the classification is 141 Level 1, 41 Level 2, 9 Level 3, and 4 Level 4 publications. Level 3 comprises references 73, 81, 84, 88, 138, 169, 170, 179, and 192; Level 4 comprises references 172, 188, 189, and 196.
